# Ultramicroporous Lonsdaleite Topology MOF with High Propane Uptake and Propane/Methane Selectivity for Propane Capture from Simulated Natural Gas

**DOI:** 10.1021/acsmaterialslett.3c01157

**Published:** 2023-12-01

**Authors:** Chenghua Deng, Li Zhao, Mei-Yan Gao, Shaza Darwish, Bai-Qiao Song, Debobroto Sensharma, Matteo Lusi, Yun-Lei Peng, Soumya Mukherjee, Michael J. Zaworotko

**Affiliations:** † Department of Chemical Sciences, Bernal Institute, University of Limerick, Limerick V94 T9PX, Ireland; ‡ Department of Applied Chemistry, College of Science, China University of Petroleum-Beijing, Beijing 102249, China

## Abstract

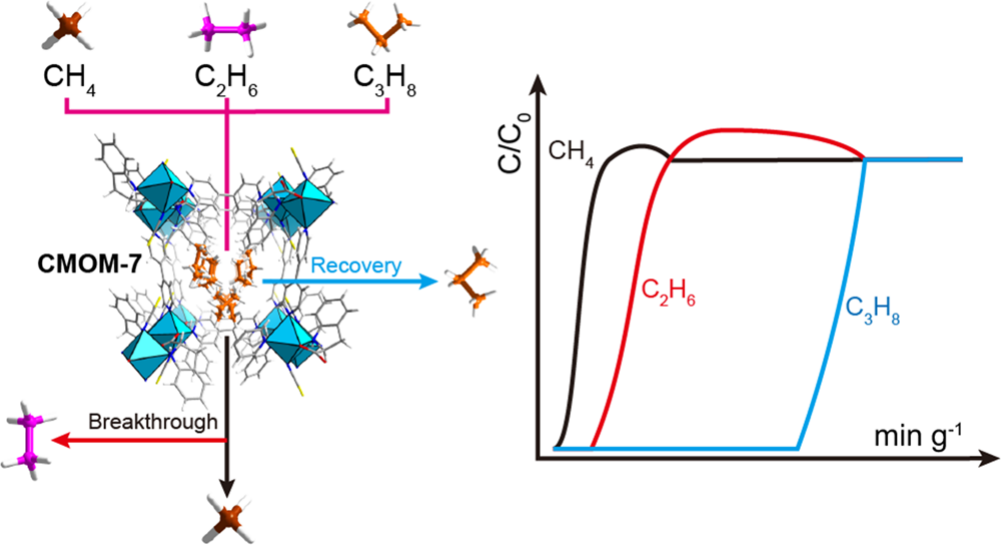

Propane (C_3_H_8_) is a widely used fuel gas. Metal–organic framework (MOF) physisorbents that are C_3_H_8_ selective offer the potential to significantly reduce the energy footprint for capturing C_3_H_8_ from natural gas, where C_3_H_8_ is typically present as a minor component. Here we report the C_3_H_8_ recovery performance of a previously unreported lonsdaleite, **lon**, topology MOF, a chiral metal–organic material, [Ni(*S*-IEDC)(bipy)(SCN)]_n_, **CMOM-7**. **CMOM-7** was prepared from three low-cost precursors: Ni(SCN)_2_, *S*-indoline-2-carboxylic acid (*S*-IDECH), and 4,4′-bipyridine (bipy), and its structure was determined by single crystal X-ray crystallography. Pure gas adsorption isotherms revealed that **CMOM-7** exhibited high C_3_H_8_ uptake (2.71 mmol g^–1^) at 0.05 bar, an indication of a higher affinity for C_3_H_8_ than both C_2_H_6_ and CH_4_. Dynamic column breakthrough experiments afforded high purity C_3_H_8_ capture from a gas mixture comprising C_3_H_8_/C_2_H_6_/CH_4_ (v/v/v = 5/10/85). Despite the dilute C_3_H_8_ stream, **CMOM-7** registered a high dynamic uptake of C_3_H_8_ and a breakthrough time difference between C_3_H_8_ and C_2_H_6_ of 79.5 min g^–1^, superior to those of previous MOF physisorbents studied under the same flow rate. Analysis of crystallographic data and Grand Canonical Monte Carlo simulations provides insight into the two C_3_H_8_ binding sites in **CMOM-7**, both of which are driven by C–H···π and hydrogen bonding interactions.

Propane (C_3_H_8_) is a widely utilized hydrocarbon that is a gas under ambient conditions. As a combustible and highly flammable fuel gas,^1−8^ compression of C_3_H_8_ is key to its storage and delivery as liquified petroleum gas.^1,2^ Thanks to its thermodynamic properties, C_3_H_8_ is also recognized as an eco-friendly refrigerant.^3,4^ Further, C_3_H_8_ is involved in production of high-value chemicals, polypropylene, and polyethylene.^5,6^ In addition, in semiconductor manufacturing, C_3_H_8_ is a precursor gas for silicon carbide deposition.^7,8^

C_3_H_8_ is generally produced by either of two routes: 1) extraction from petroleum refining; 2) extraction from natural gas (NG). NG is a mixture of hydrocarbons, primarily composed of methane (CH_4_) and smaller amounts of ethane (C_2_H_6_), C_3_H_8_, and other gases.^1,9−11^ Low C_3_H_8_ concentration in NG pipelines is desirable as it mitigates condensation reactions.^12^Scheme 1 illustrates the preparation of C_3_H_8_ and its utility as a commodity chemical. The global C_3_H_8_ market reached 174.3 million tons in 2022, with a 2028 market forecast as high as 223.1 million tons.^13^ Despite this surging demand, energy-intensive distillation remains state-of-the-art to obtain C_3_H_8_ from petroleum refining products and NG.^14,15^ Overall, distillation processes account for 45–55% of the energy footprint of the chemical industry, a sector that consumes ca. 15% of global energy consumption.^16^ To mitigate this energy footprint, adsorptive separation using physisorbents is emerging as an alternative to distillation and extractions.^16−18^ Specifically, C_3_H_8_-selective physisorbents that capture C_3_H_8_ from NG are of interest as they will save energy, potentially leading to process optimization for NG purification.^19−21^

**Scheme 1:**
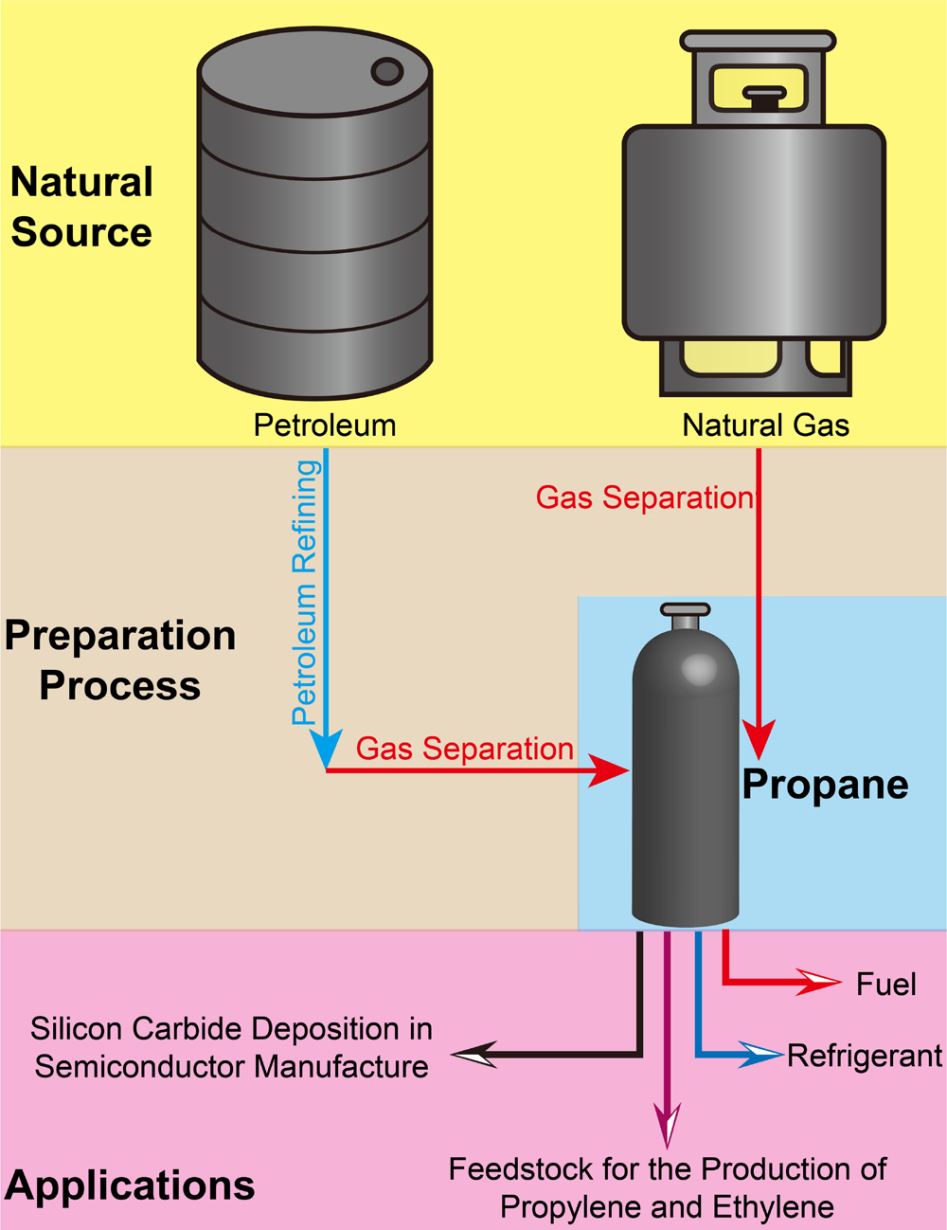
Preparation and Application of Propane

Metal–organic materials (MOMs), also known as porous coordination polymers (PCPs) and metal–organic frameworks (MOFs), are porous materials typically comprised of coordination bond-linked metal cations or metal clusters and organic ligands that serve as linkers.^22−25^ Compared to traditional porous materials like zeolites, porous carbons and silica, MOMs are amenable to crystal engineering,^26^ enabling chemists to control pore size, shape and chemistry.^18,19,26−30^ In the context of NG purification, molar mass and polarizability gradually increase from CH_4_ to C_2_H_6_ to C_3_H_8_ (Table S3).^31^ The kinetic diameters of C_2_H_6_ (4.44 Å) and C_3_H_8_ (4.3 Å) are near-identical, both higher than that of CH_4_ (3.76 Å). A CH_4_/C_2_H_6_/C_3_H_8_ (85:10:5, v/v) ternary mixture is generally regarded as a suitable composition for studying the NG purification potential of a sorbent. This separation remains understudied with physisorbents tending to coadsorb C_2_H_6_ and C_3_H_8_, thereby affording high-purity CH_4_.^32−38^ To the best of our knowledge, only three studies have demonstrated separation of C_3_H_8_ from C_2_H_6_ by MOMs from a relevant CH_4_/C_2_H_6_/C_3_H_8_ (85:10:5) gas mixture.^39−41^ Developing an MOM with both high selectivity and high dynamic uptake for C_3_H_8_ is a challenge that we address herein.

We have previously reported that rod building blocks (RBBs) and low-cost abundant linker ligands, e.g., mandelic acid and *S*-indoline-2-carboxylic acid (*S*-IDECH), can afford families of chiral MOMs, CMOMs.^42−46^ These CMOMs are modular as they are composed of divalent metal cations, e.g., Co^2+^ or Zn^2+^, that form RBBs involving mandelate or related anions cross-linked by 4,4′-bipyridine (bipy) linkers. These structures can form cationic **bnn** or **dia** porous coordination networks with extra-framework counteranions. In this study, we introduce a new CMOM variant which contains a coordinated thiocyanate, and report its gas sorption properties and separation performance.

## Experimental Section

Reagents (≥98% purity) and solvents were procured from commercial vendors and used without further purification, except for nickel(II) thiocyanate (Ni(SCN)_2_), which was prepared by the reaction of nickel(II) carbonate (NiCO_3_) and potassium thiocyanate (KSCN).^47^ Full details on synthesis procedures are given in the experimental section of the Supporting Information.

### Synthesis and Solvent Exchange

A methanolic solution of Ni(SCN)_2_ was added to an *N,N*-dimethylformamide (DMF) solution of bipy, and *S*-IDECH (molar ratios of Ni(SCN)_2_: bipy: *S*-IDECH = 1:1:1) in a 15 mL glass vial. Blue crystals of {[Ni(*S*-IDEC)(bipy)(SCN)](DMF)_1.5_}_n_, **CMOM-7-DMF**, were obtained after the vial was heated in an oven at 85 °C for 24 h. After soaking crystals of **CMOM-7-DMF** in methanol for 5 days with fresh solvent exchange every day, the crystals transformed into {[Ni(*S*-IDEC)(bipy)(SCN)](MeOH)_3_}_n_, **CMOM-7-MeOH**. Crystals of **CMOM-7-MeOH** were used for subsequent gas sorption and dynamic column breakthrough experiments.

### Pure Gas Sorption Isotherms

A sample of **CMOM-7-MeOH** was activated under high vacuum using a Micromeritics Smart VacPrep at 60 °C for 12 h before gas sorption experiments. The crystals were found to transform to [Ni(*S*-IDEC)(bipy)(SCN)]_n_, the activated form of **CMOM-7**. 77 K N_2_ and 195 K CO_2_ sorption isotherms were recorded by a Micromeritics TriStar II Plus surface area and porosity analyzer. CO_2_, CH_4_, acetylene (C_2_H_2_), ethylene (C_2_H_4_), C_2_H_6_, C_3_H_8_, propylene (C_3_H_6_) and C_3_H_4_ sorption isotherms were collected with a Micromeritics 3Flex adsorption analyzer.

### Dynamic Column Breakthrough (DCB) Experiment

About 0.88 g of activated **CMOM-7** was used as a packed fixed-bed in quartz tubing (Φ 6 × 400 mm, outer diameter = 8 mm) inside a dynamic column breakthrough instrument (Figures S22 and S23).^48^ The packed sample was purged under a 20 cm^3^ min^–1^ flow of helium at 80 °C for 1 h prior to conducting each breakthrough experiment. The composition of the outlet gas during the breakthrough experiments was monitored by a Shimadzu Nexis GC-2030 gas chromatograph (GC) with a flame ionization detector (FID) and a thermal conductivity detector (TCD).

**Figure 1:**
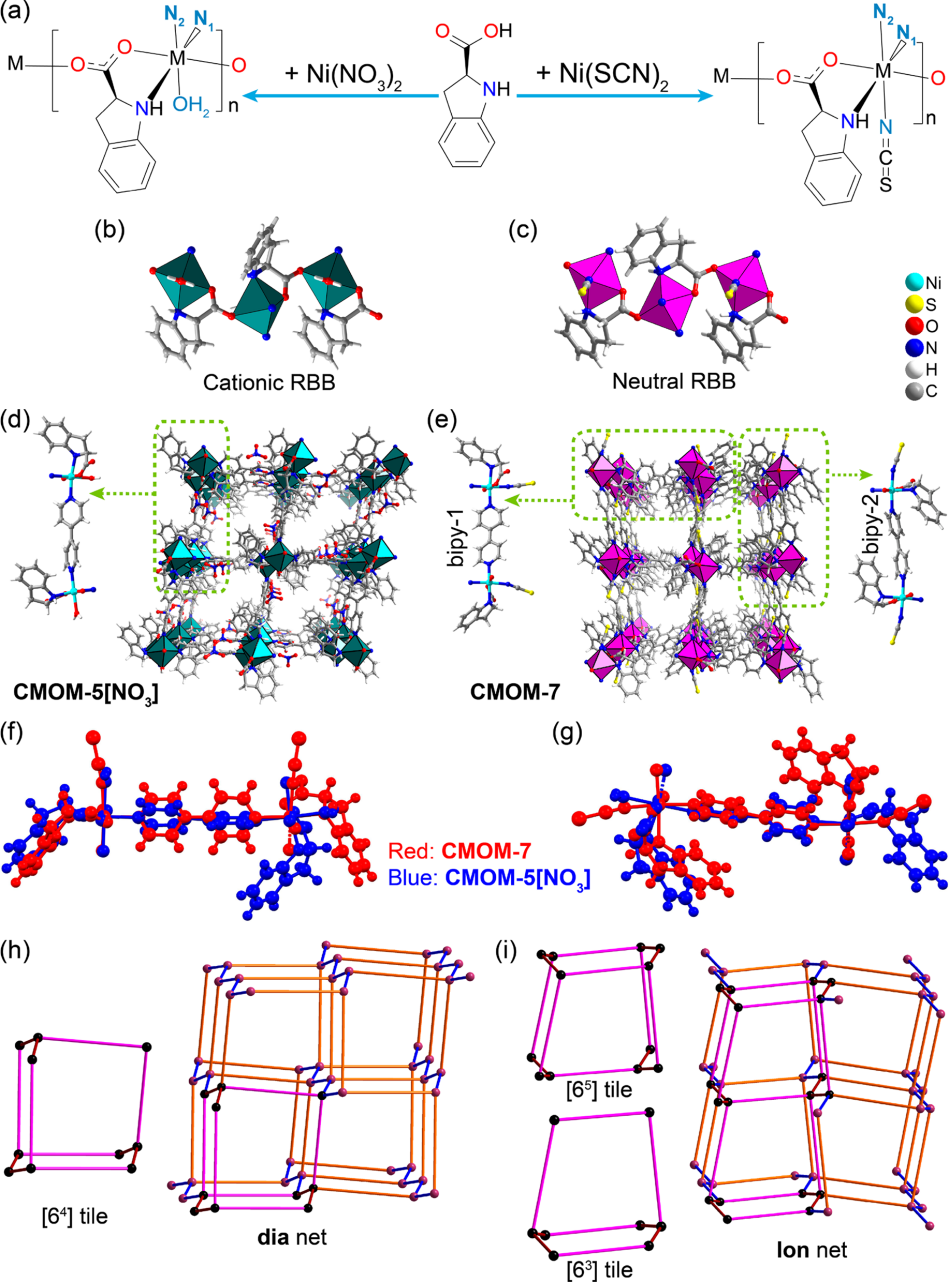
Structural differences between **CMOM-5** and **CMOM-7**. (a) Schematic illustration of the repeating units that form the RBBs of **CMOM-5** (left) and **CMOM-7** (right); **N**_**1**_ and **N**_**2**_ correspond to the bipy linkers opposite and perpendicular to the terminally coordinated ligands, respectively. (b) The cationic RBB of **CMOM-5**. (c) Neutral RBB of **CMOM-7**. (d) The crystal structure of **CMOM-5**, and the coordination geometry around bipy (left). (e) The crystal structure of **CMOM-7**, including the coordination geometries around bipy-1 (left) and bipy-2 (right). (f) Overlay plot of a bipy linker from **CMOM-5** and bipy-1 of **CMOM-7**. (g) Overlay plot of a bipy linker from **CMOM-5** and bipy-2 of **CMOM-7**. (h) The **dia** network topology of **CMOM-5** and the [6^4^] tile generated thereof. (i) The **lon** network topology of **CMOM-7**, alongside the frameworks of [6^5^] and [6^3^] tiles generated thereof. Solvent molecules were omitted for clarity.

We recently reported the structure of **CMOM-5[NO**_**3**_**]** ([Ni(*S*-IDEC)(bipy)(H_2_O)][NO_3_]) in which nickel cations are octahedrally coordinated to *S*-IDEC, bipy and an aqua ligand to afford a cationic RBB (Figure 1a,b) which, when connected by bipy linkers, afforded a cationic **dia** net.^46^**CMOM-7** was prepared using Ni(SCN)_2_ rather than Ni(NO_3_)_2_. Single-crystal X-ray diffraction (SCXRD) studies revealed that the as-synthesized phase, {[Ni(*S*-IDEC)(bipy)(SCN)](DMF)_1.5_}_n_, **CMOM-7-DMF**, had crystallized in the monoclinic space group *P2*_1_2_1_2_1_ (Table S1), the same as that of **CMOM-5[NO**_**3**_**]**. The asymmetric unit of **CMOM-7-DMF** was found to contain three DMF molecules, a pair of Ni^2+^ cations coordinated to two thiocyanate anions, two *S*-IDEC anions, and two bipy ligands. The Ni^2+^ cations in **CMOM-7** exhibit the same coordination environment as that in **CMOM-5** but with the aqua ligand of **CMOM-5** substituted by thiocyanate (Figure 1a,c). The ratio of Ni^2+^/S-IDEC/bipy in both **CMOM-5** and **CMOM-7** is 1:1:1 and, as the RBB in **CMOM-7** is neutral,^46,49^ there are no extra-framework anions.

In the *S*-IDEC RBBs, we define the coordination sites of bipy as **N**_**1**_ and **N**_**2**_, corresponding to the positions opposite the nitrogen atom from S-IDEC or opposite the terminal coordinated ligands (aqua ligand and SCN^–^), respectively (Figure 1a). The asymmetric unit of **CMOM-5[NO**_**3**_**]** is comprised of one bipy coordinated to two Ni^2+^ sites through **N**_**1**_ and **N**_**2**_ positions (Figure 1d). Conversely, the asymmetric unit of **CMOM-7-DMF** contains two bipy linkers, one linked to two nickel cations through the **N**_**1**_ sites, and another linked to two nickel cations through the **N**_**2**_ sites (Figure 1e); bipy-1 and bipy-2, respectively. Overlay plots of the frameworks of **CMOM-5[NO**_**3**_**]** and **CMOM-7** around the bipy linkers reveals that the RBBs are linked differently (Figure 1f,g). Specifically, RBBs in **CMOM-5** lie parallel to the crystallographic *a* axis (Figures 1d and S2), whereas in **CMOM-7** RBBs extend along the crystallographic *c* axis with adjacent RBBs being antiparallel (Figures 1e and S2). The topologies of **CMOM-5** and **CMOM-7** are diamondoid, **dia**, and Lonsdaleite, **lon**, respectively. Both 4-c topologies have the same point ({6^6^}) and vertex symbols ({6_2_.6_2_.6_2_.6_2_.6_2_.6_2_}). However, the **dia** net is based upon [6^4^] tiles (Figure 1h), whereas the **lon** net comprises from [6^3^] and [6^5^] tiles (Figure 1i).^50,51^

**CMOM-7-DMF** was activated by soaking in methanol, transforming it to **CMOM-7-MeOH**, and exposure to vacuum. **CMOM-7-MeOH** and activated **CMOM-7** retained space group *P2*_1_2_1_2_1_ (Tables S1 and S2) with unit cell parameters similar to **CMOM-7-DMF**. The unit cell volume of **CMOM-7** (5286.10(19) Å^3^) was found to be slightly smaller than those of **CMOM-7-DMF** (5365.1(3) Å^3^) and **CMOM-7-MeOH** (5382.4(2) Å^3^). Powder X-ray diffraction (PXRD) diffractograms demonstrated phase purity and retention of crystallinity (Figure S5). Variable temperature PXRD (VT-PXRD) studies conducted upon **CMOM-7-MeOH** revealed the retention of crystallinity below 225 °C (Figure S8). Thermogravimetric analysis (TGA) conducted upon **CMOM-7-DMF** revealed a weight loss of ca. 18 wt % at ca. 180 °C (Figure S9), consistent with loss of DMF guest molecules, and thermal decomposition starting at ca. 255 °C. TGA data for **CMOM-7-MeOH** revealed a weight loss of 14.11 wt % at 73 °C, corresponding to the loss of methanol. **CMOM-7** did not register any significant weight loss before thermal decomposition at ca. 255 °C.

**Figure 2:**
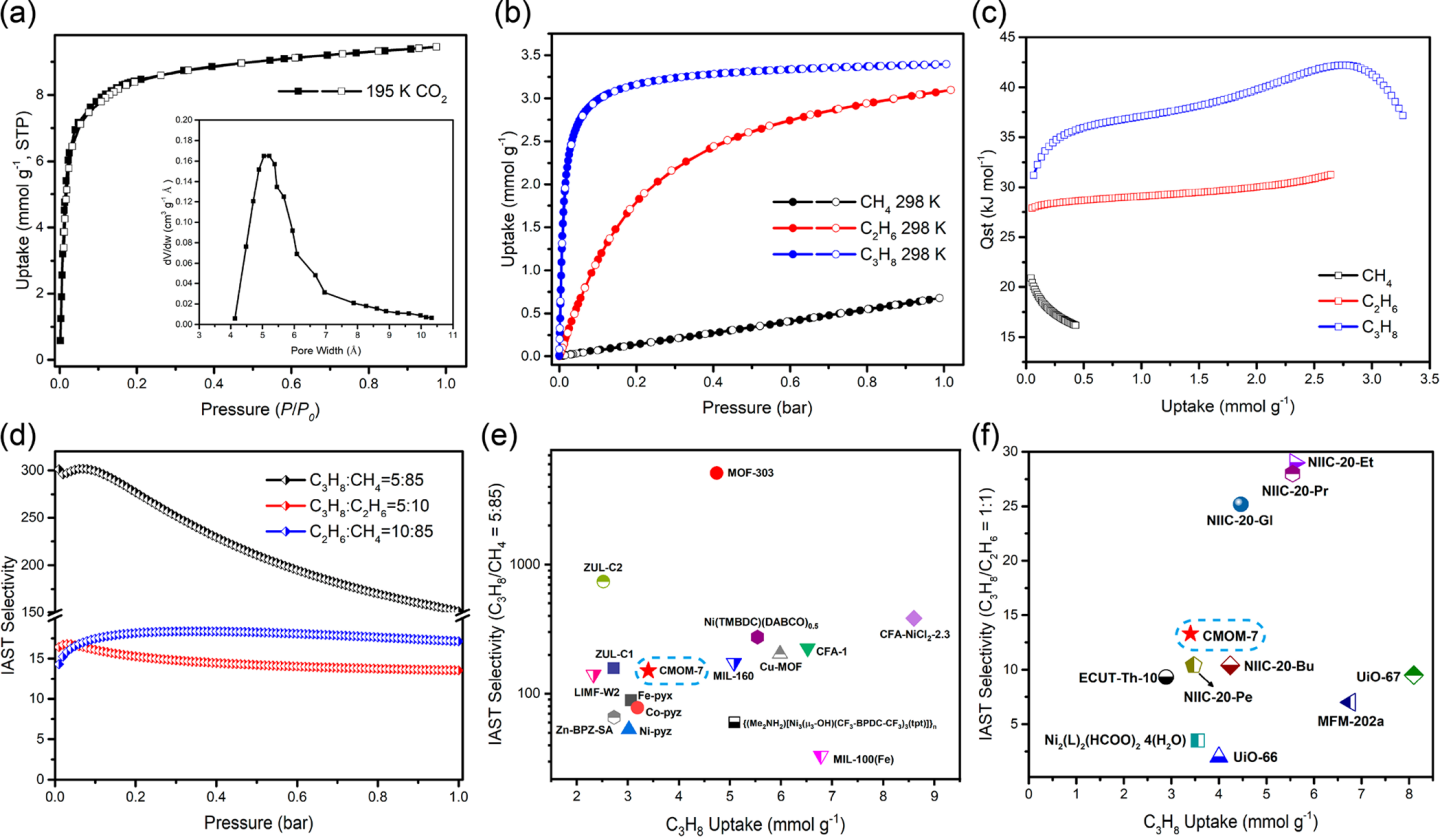
Single-component gas sorption studies of **CMOM-7**. (a) 195 K CO_2_ sorption isotherm of **CMOM-7** and the corresponding H–K pore size distribution profile (inset). (b) CH_4_, C_2_H_6_ and C_3_H_8_ adsorption isotherms of **CMOM-7** at 298 K. (c) Isosteric enthalpies of adsorption (*Q*_st_) of **CMOM-7** for CH_4_, C_2_H_6_ and C_3_H_8_. (d) IAST selectivity of **CMOM-7** for three binary mixtures of compositions: C_3_H_8_/CH_4_ (v/v = 5:85), C_3_H_8_/C_2_H_6_ (v/v = 5:10) and C_2_H_6_/CH_4_ (v/v = 10:85). (e) Comparison of C_3_H_8_ adsorption capacity and C_3_H_8_/CH_4_ (v/v = 5:85) IAST selectivities for a few select reported sorbents at 298 K and 1 bar. (f) Comparison of C_3_H_8_ adsorption capacity and C_3_H_8_/C_2_H_6_ (v/v = 1:1) IAST selectivities for a few select reported sorbents at 298 K and 1 bar.

To investigate porosity, 195 K CO_2_ and 77 K N_2_ gas sorption experiments were conducted on **CMOM-7**, both of which exhibited typical Type-I isotherms^52^ with saturation uptakes of 9.44 mmol g^–1^ (CO_2_, 195 K) and 9.48 mmol g^–1^ (N_2_, 77 K), respectively (Figures 2a and S11). High-pressure CO_2_ sorption also resulted in a Type-I isotherm, registering an uptake of 7.32 mmol g^–1^ at 298 K and 40 bar (Figure S12). Langmuir surface areas determined from the 195 K CO_2_ and 77 K N_2_ sorption isotherms were consistent, 971.04 m^2^ g^–1^ and 919.11 m^2^ g^–1^, respectively (Figure S14). The BET (Brunauer–Emmett–Teller) surface areas determined from 195 K CO_2_ and 77 K N_2_ data were 868.01 m^2^ g^–1^ and 861.38 m^2^ g^–1^, respectively (Figure S15). Based on Horvath–Kawazoe (H–K) pore size distribution analysis of the 195 K CO_2_ and 77 K N_2_ adsorption isotherms, the mean pore width of **CMOM-7** is calculated to be 5.3 and 8.9 Å, respectively (Figures 2a and S11).

The single-component gas sorption isotherms of C1 gases (CH_4_ and CO_2_), C2 gases (C_2_H_2_, C_2_H_4_ and C_2_H_6_) and C3 gases (C_3_H_8_, C_3_H_6_ and C_3_H_4_) were recorded at three temperatures, 273, 298, and 313 K (Figures 2b, S16 and S17), all of which are Type-I isotherms.^52^ At 298 K and 1 bar, **CMOM-7** exhibited 1 bar uptakes for CH_4_, C_2_H_6_ and C_3_H_8_ of 0.68 mmol g^–1^, 3.10 mmol g^–1^ and 3.40 mmol g^–1^, respectively. At 298 K and 0.05 bar, the uptake of C_3_H_8_, 2.71 mmol g^–1^, was higher than that of C_2_H_6_ (0.61 mmol g^–1^) and much higher than that of CH_4_ (0.04 mmol g^–1^). The half-loading (0.05 bar) C_3_H_8_ uptake is comparable to leading NG separation sorbents such as **MIL-160** (2.48 mmol g^–1^),^36^**MOF-303** (3.38 mmol g^–1^),^36^**BSF-2** (0.79 mmol g^–1^),^53^ and **0.3Gly@HKUST-1** (4.22 mmol g^–1^).^32^ That the C_3_H_8_ isotherm is much steeper than the C_2_H_6_ and CH_4_ isotherms suggests that sorbate–sorbent interactions increase from CH_4_ to C_2_H_6_ to C_3_H_8_, as expected from their sizes and polarizabilities. To further explore the affinity of **CMOM-7** toward pure gases, the single-component adsorption isotherms of CH_4_ and C_2_H_6_ were fitted using the single-site Langmuir Freundlich (SSLF) model (Figures S18, S19), and those of C_3_H_8_ were fitted to the dual-site Langmuir Freundlich (DSLF) model (Figure S20, Tables S4 and S5). From the pure gas isotherms recorded at 273, 298, and 313 K, the corresponding isosteric enthalpies of adsorption (*Q*_st_) values at low surface coverage were determined to be 20.9, 27.9, and 31.2 kJ mol^–1^, respectively (Figure 2c), once again indicating that **CMOM-7** exhibits stronger affinity for C_3_H_8_ than C_2_H_6_ and CH_4_.

Ideal adsorbed solution theory (IAST) selectivity values for binary mixtures C_3_H_8_/C_2_H_6_, C_2_H_6_/CH_4_, and C_3_H_8_/CH_4_ were calculated from the 298 K pure gas isotherms. The IAST selectivities for C_3_H_8_/CH_4_ (5:85 and 1:1), C_3_H_8_/C_2_H_6_ (5:10 and 1:1), and C_2_H_6_/CH_4_ (10:85 and 1:1) at 1 bar were found to be 151.4, 40.1 (C_3_H_8_/CH_4_), 13.6, 13.3 (C_3_H_8_/C_2_H_6_), 17.1 and 12.5 (C_2_H_6_/CH_4_) (Figures 2d and S21). The boiling points for CH_4_, C_2_H_6_, and C_3_H_8_, 111.6, 184.6, and 231 K, respectively, are consistent with the general expectation that IAST selectivities correlate with adsorbate boiling points. These IAST selectivities indicate potential for ternary mixture recovery of C_3_H_8_ from CH_4_ and C_2_H_6_, being competitive for both C_3_H_8_/CH_4_ and C_3_H_8_/C_2_H_6_ (Figure 2e,f and Table S8). Specifically, whereas the C_3_H_8_/CH_4_ (5:85) IAST selectivity for **CMOM-7** exceeds that of **MIL-100(Fe)** (33.3),^37^**MIL-101-Fe-NH**_**2**_ (42.5),^40^**Zn-BPZ-SA** (65.7),^38^**Fe-pyz** (89),^54^ it is lower than that of **MOF-303** (5114),^36^**Ni(TMBDC)(DABDC)**_**0.5**_ (274),^34^**CFA-1-NiCl**_**2**_**-2.3** (382.7),^55^ and **ZUL-C2** (741).^56^ The C_3_H_8_/C_2_H_6_ (1:1) IAST selectivity of **CMOM-7** exceeds **ECUT-Th-10** (9.31),^39^**UiO-67** (∼9.5),^39^**MFM-202a** (∼7),^57^**NIIC-20-Bu** (10.4),^41^ and **Ni**_**2**_**(L)**_**2**_**(HCOO)**_**2**_**(H**_**2**_**O)**_**4**_ (L = 3-hydroxy-4-(4H-1,2,4-triazol-4-yl)benzoate)^58^ but is lower than **NIIC-20-Et** (29.0),^41^**NIIC-20-Pr** (28.0),^41^ and **NIIC-20-Gl** (25.2).^41^

**Figure 3:**
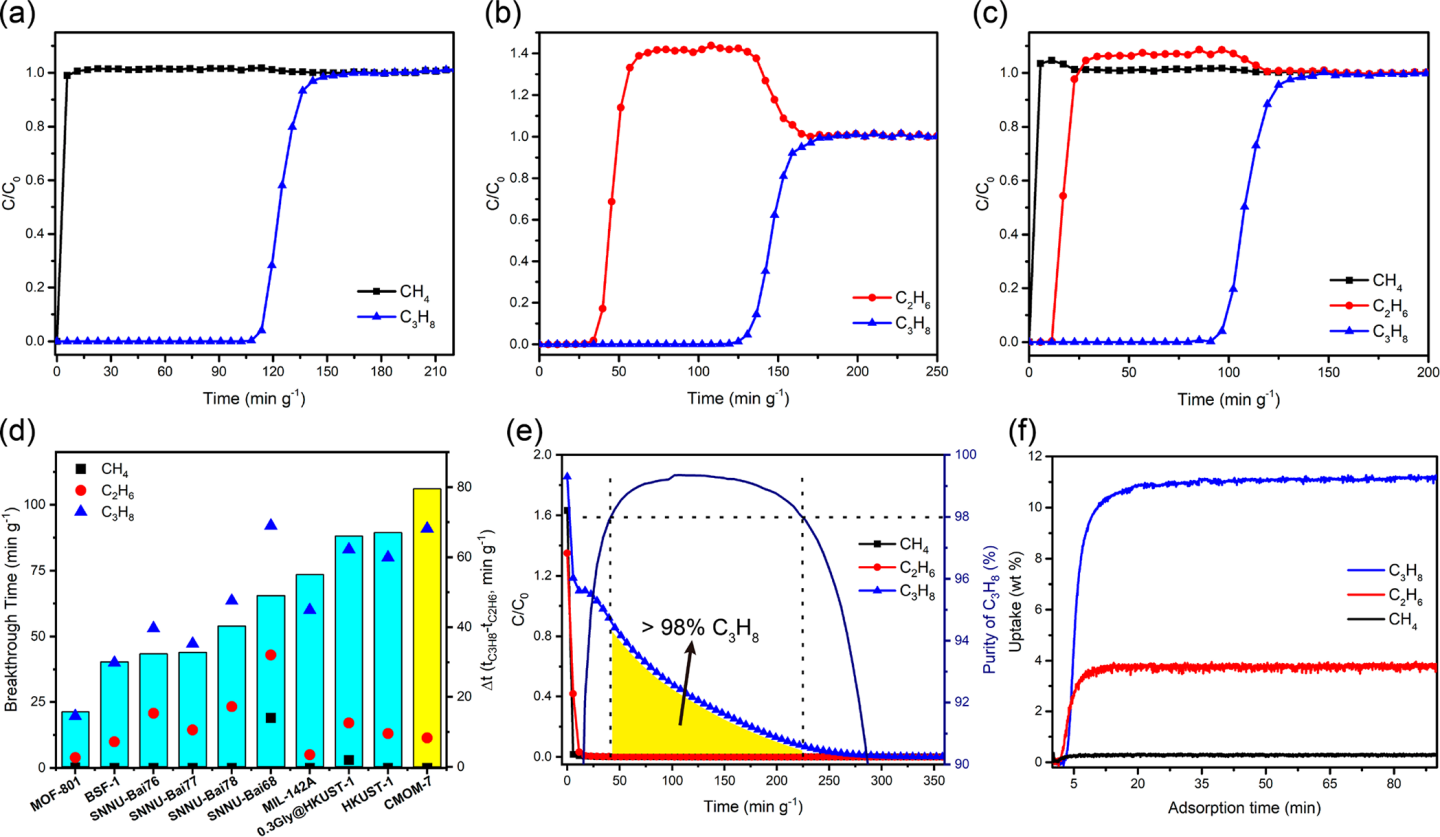
Breakthrough study results on **CMOM-7** for CH_4_, C_2_H_6_ and C_3_H_8_ gas mixtures at 298 K and 1 bar. (a) The breakthrough profiles for C_3_H_8_/CH_4_ (v/v = 5:85, 9 mL min^–1^) gas mixture. (b) The breakthrough profiles for C_3_H_8_/C_2_H_6_ (v/v = 5:10, 1.5 mL min^–1^) gas mixture. (c) The breakthrough profiles for C_3_H_8_/C_2_H_6_/CH_4_ (v/v/v = 5:10:85, 10 mL min^–1^) gas mixture. (d) For the C_3_H_8_/C_2_H_6_/CH_4_ (v/v/v = 5:10:85, 10 mL min^–1^) DCB experiments, performance comparison of **CMOM-7** with reported materials on their relative CH_4_, C_2_H_6_, and C_3_H_8_ breakthrough times; and their breakthrough time differences (Δ*t*) (between C_3_H_8_ and C_2_H_6_). (e) Temperature-programmed desorption profiles recorded under 50 mL min^–1^ He flow after the C_3_H_8_/C_2_H_6_/CH_4_ (v/v/v = 5:10:85, 10 mL min^–1^) ternary mixture breakthrough experiment-based bed saturation. (f) Gravimetric adsorption kinetics for CH_4_, C_2_H_6_ and C_3_H_8_ on **CMOM-7**, plotted as uptake (wt %) vs time (min).

To determine the separation performance, DCB experiments of binary mixtures were performed under ambient conditions. For C_3_H_8_/CH_4_ (v/v = 5:85, total flow = 9 mL min^–1^) and C_2_H_6_/CH_4_ (v/v = 10:85, total flow = 9.5 mL min^–1^), CH_4_ was found to elute immediately, whereas C_3_H_8_ and C_2_H_6_ eluted at 108 and 11.4 min g^–1^, respectively (Figures 3a and S24). For C_3_H_8_/C_2_H_6_ (v/v = 5:10, 1.5 mL min^–1^), C_2_H_6_ and C_3_H_8_ eluted at 34.1 and 119.3 min g^–1^, respectively (Figure 3b). **CMOM-7** adsorbed 72.7 mL g^–1^ (3.24 mmol g^–1^) C_3_H_8_ and 3.9 mL g^–1^ (0.17 mmol g^–1^) C_2_H_6_, resulting in C_3_H_8_/C_2_H_6_ selectivity of 37.3. We next conducted DCB experiments on a ternary C_3_H_8_/C_2_H_6_/CH_4_ (v/v/v = 5:10:85) mixture with a total flow of 10 mL min^–1^. CH_4_ eluted immediately and 3.4 mmol g^–1^ of high-purity CH_4_ (≥98%) was produced before C_2_H_6_ and C_3_H_8_ passed through the fixed bed at 11.4 min g^–1^ and 90.9 min g^–1^, respectively (Figure 3c). The breakthrough time difference (Δ*t*) between C_3_H_8_ and C_2_H_6_ was 79.5 min g^–1^. To the best of our knowledge, this Δ*t* value exceeds MOFs studied with the same ternary mixture and flow rate (Figure 3d and Table S9): **SNNU-Bai-68** (49 min g^–1^),^33^**SNNU-Bai78** (40.3 min g^–1^),^59^**0.3Gly@HKUST-1** (66 min g^–1^),^32^**HKUST-1** (67 min g^–1^),^32^**MOF-801** (15.72 min g^–1^),^60^**MIL-142A** (55 min g^–1^),^61^ and **BSF-1** (30.1 min g^–1^),^62^ inter alia. During the DCB experiment, 0.48 mmol g^–1^ C_2_H_6_ and 2.45 mmol g^–1^ C_3_H_8_ were found to be absorbed by **CMOM-7** (Table S6). The dynamic uptake of C_3_H_8_ exceeded several NG separating sorbents including **BSF-2** (∼0.76 mmol g^–1^),^53^**C-PVDC-800** (3.02 mmol g^–1^),^63^**ZUL-C1** (1.90 mmol g^–1^),^56^**ZUL-C2** (1.92 mmol g^–1^),^56^ and **MIL-101-Cr** (0.60 mmol g^–1^).^40^

The **CMOM-7** packed column was regenerated by using a helium flow (50 mL min^–1^). As revealed by Figure 3e, CH_4_ and C_2_H_6_ were removed within 11.4 min g^–1^, while C_3_H_8_ took 360 min g^–1^ (Figure 3e). 1.6 mmol g^–1^ high-purity (≥98%) C_3_H_8_ was obtained during the interim period (39.8 to 221.6 min g^–1^). The stability of the packed column was investigated by DCB cycling under identical conditions (Figure S25). Breakthrough times and dynamic uptakes remained almost unchanged over three cycles (Table S6). We also studied DCB performance with total flow rates of 9, 11, and 12 mL min^–1^ (Figure S26). CH_4_ always eluted immediately; breakthrough times for C_2_H_6_ were 11.4 min g^–1^ at 9 mL min^–1^ and 5.7 min g^–1^ at 11 and 12 mL min^–1^. Under flow rates of 9, 10, and 12 mL min^–1^, breakthrough times for C_3_H_8_ were 102.3, 85.2, and 79.5 min g^–1^, respectively.

Adsorption kinetics for CH_4_, C_2_H_6_ and C_3_H_8_ were assessed by exposing **CMOM-7** to 10 mL min^–1^ flows of CH_4_, C_2_H_6_ and C_3_H_8_ at 303 K and 1 bar, separately. **CMOM-7** adsorbed 0.29 wt % (0.18 mmol g^–1^) of CH_4_ in 5 min, and 3.67 wt % (1.22 mmol g^–1^) of C_2_H_6_ in 10 min. 10.88 wt % (2.48 mmol g^–1^) of C_3_H_8_ was adsorbed in 20 min (Figure 3f). Consistent kinetic results over three consecutive sorption cycles were found (Figure S10). Adsorption kinetics for C_3_H_8_ and C_2_H_6_ exhibited similar slopes at low loading, suggesting that the separation performance is not driven by kinetics alone. Nevertheless, under an identical flow rate and other conditions, **CMOM-7** adsorbed a higher amount of C_3_H_8_ than C_2_H_6_. This suggests that the C_3_H_8_ selectivity is driven by a combination of optimal thermodynamics and kinetics, a phenomenon we have previously observed in ultramicroporous sorbents that exhibit benchmark CO_2_ and C_2_ trace capture.^28,64,65^

To assess hydrolytic stability, crystals of **CMOM-7-MeOH** were soaked in water for a day (Figure S1). Water molecules exchanged with methanol molecules, affording the water-loaded structure {[Ni(*S*-IDEC)(bipy)(SCN)](H_2_O)_6.5_}_n_, **CMOM-7-H**_**2**_**O**, as determined by SCXRD. **CMOM-7-H**_**2**_**O** crystallized in space group *C222*_1_ (Table S2). PXRD patterns (Figure S7) verified the stability in water and at 75% relative humidity (RH). **CMOM-7** did not change structure after solvent exchange (Figure S3). TGA data revealed weight loss of 17.62 wt % at 63 °C (Figure S9).

Seven water binding sites were identified in **CMOM-7-H**_**2**_**O**, interacting with the pore walls through O–H···O and O–H···S hydrogen bonds (H-bonds) involving the O atoms of *S*-IDEC and S atoms of SCN^–^ ligands (Figure S4). The dynamic vapor sorption (DVS) H_2_O isotherm for **CMOM-7** exhibited a sigmoidal *S* shape (Figure S28)^44^ with low uptake of water (1.56 wt %) at 30% RH followed by hysteresis and uptake of 20.19 and 23.94 wt % at 55% RH and 95% RH, respectively. The water adsorption kinetics revealed that **CMOM-7** had uptake of 22.5 wt % within 40 min at 60% RH (Figure S29). Water vapor sorption cycling was also conducted. In the first cycle, **CMOM-7** exhibited an uptake of 19.29 wt % at 60% RH in 25 min and uptake of 18.35 wt % at the tenth cycle (Figure S30a). After 100 cycles, uptake had dropped to 14.69 wt % (Figure S30b), indicating partial collapse of **CMOM-7**. A water-soaked sample of **CMOM-7** exhibited an uptake of 3.31 mmol g^–1^, similar to pristine **CMOM-7** (3.4 mmol, Figures 2b and S13).

**Figure 4:**
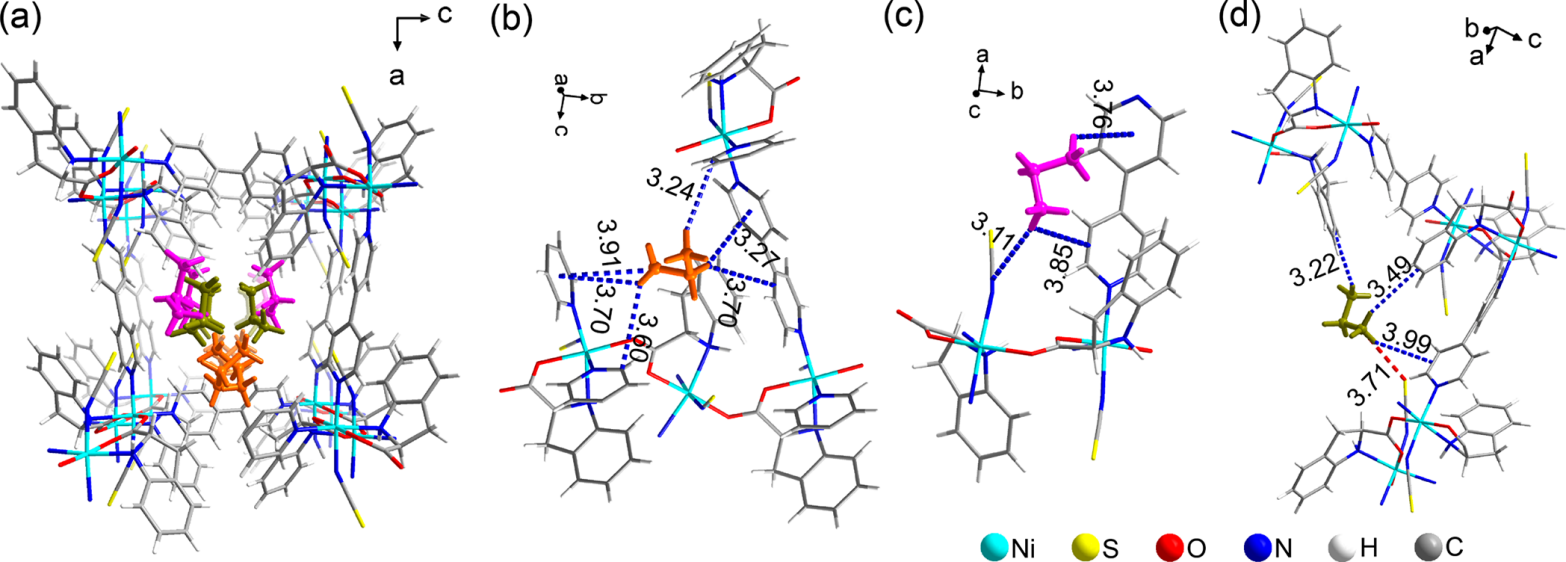
Crystallographically determined binding site of C_3_H_8_ molecules along the channel of **CMOM-7** (a) and the intermolecular interactions between C_3_H_8_ molecules and the host framework of **CMOM-7** around site-*I* (b), site-*II* part A (c) and site-*II* part B (c). Atoms of C_3_H_8_ in binding site-*I* are colored in orange, whereas atoms of C_3_H_8_ in binding site-*II* part A and site-*II* part B are colored pink and dark yellow, respectively. The C–H···π interactions are represented by blue dashed lines, and the H-bond is represented by a red dashed line. All distances are given in Angstrom units.

To gain insight into the C_3_H_8_-selective nature of **CMOM-7**, we obtained its C_3_H_8_ loaded structure using a C_3_H_8_ loaded Schlenk bottle at 195 K for 12 h (bath of dry ice) which enabled **CMOM-7** crystals to equilibrate. SCXRD study of the resulting phase allowed us to determine the crystal structure of the corresponding C_3_H_8_ loaded phase, {[Ni(*S*-IDEC)(bipy)(SCN)](C_3_H_8_)_1.5_}_n_, **CMOM-7-C**_**3**_**H**_**8**_, which had crystallized in the orthorhombic spaced group *C222*_1_ (Table S2). PXRD diffractograms (Figure S6) confirmed the phase purity and crystallinity. The asymmetric unit comprised 1.5 molecules of C_3_H_8_, with C_3_H_8_ uptake at 273 K (3.44 mmol g^–1^) matching the composition of **CMOM-7-C**_**3**_**H**_**8**_ (3.45 mmol g^–1^). Two C_3_H_8_ molecules were observed in the asymmetric unit at sites *I* and *II* (Figure 4). Cambridge Structural Database (CSD) mining revealed that this is only the tenth example of ordered C_3_H_8_ molecules determined by SCXRD (Figure S27 and Table S7). As shown in Figure 4a, C_3_H_8_ molecules in binding site *I* are 2-fold disordered in a ratio of 1:1 around an inversion center.^66^ Site *I* molecules forms C–H···π interactions with the pyridine rings of bipy linkers (3.24–3.91 Å, Figure 4b). The molecule in binding site *II* is also 2-fold disordered, with occupancies of 0.68 and 0.32 for A and B, respectively. A interacts with the host framework through C–H···π interactions on the pyridine ring of bipy (3.76–3.85 Å) and the C=N bond of the SCN^–^ ligands (3.11 Å, Figure 4c). B interacts with a) the S atoms of SCN^–^ through H-bond interactions (3.71 Å); b) the edges of the pyridine rings of bipy; and c) phenyl rings of *S*-IDEC through C–H···π interactions (3.22–3.99 Å, Figure 4d).

**Figure 5:**
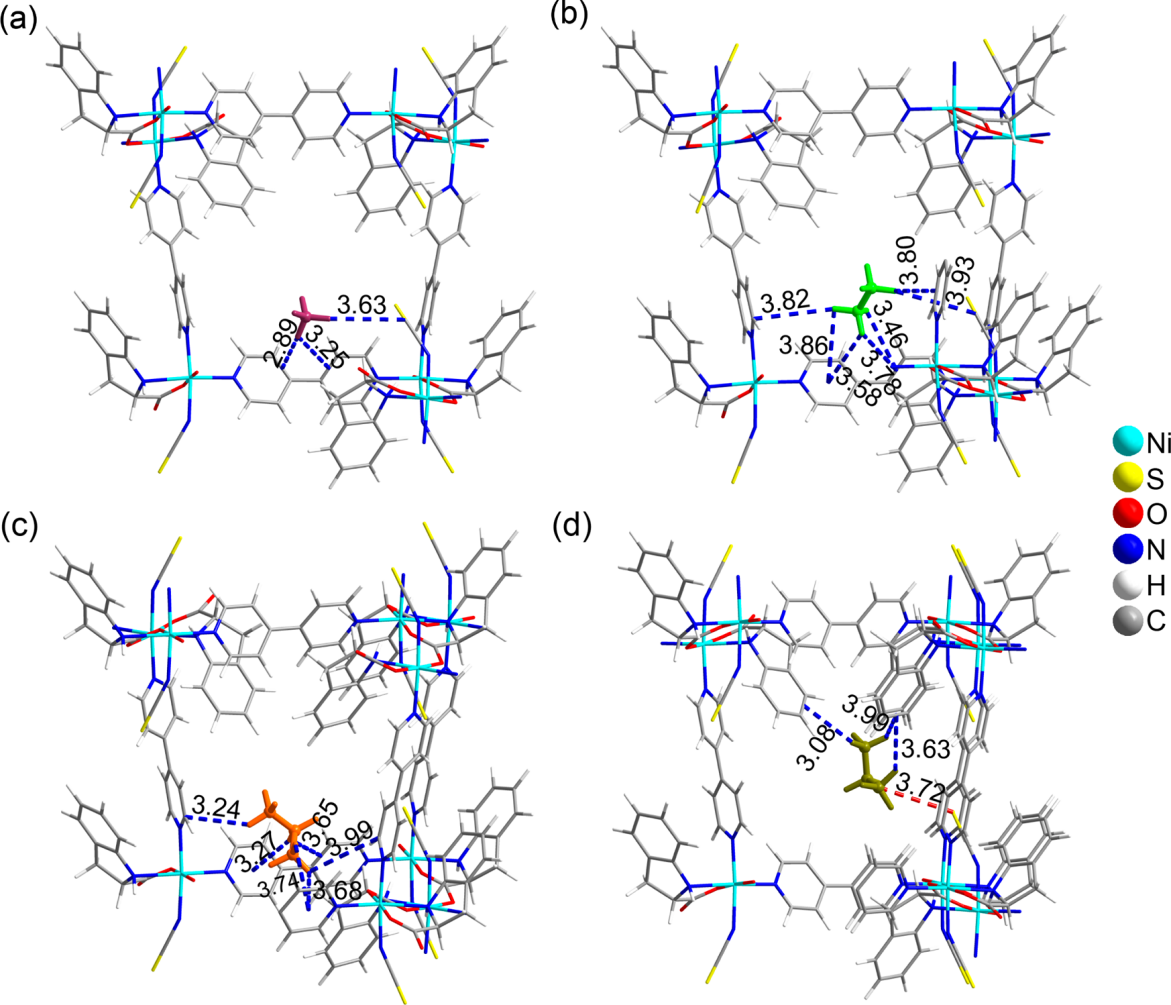
Binding sites of CH_4_ (a), C_2_H_6_ (b) and C_3_H_8_ (c, d) molecules along the ultramicroporous channel of **CMOM-7**, determined by GCMC calculations. CH_4_ and C_2_H_6_ molecules are colored mauve and light green, respectively, whereas the C_3_H_8_ molecules residing in sites *I* and *II* are presented in orange and dark yellow, respectively. C–H···π interactions are represented by blue dashed lines, whereas the H-bond is represented by a red dashed line. All distances are given Angstrom units.

Grand Canonical Monte Carlo (GCMC) simulations and first-principles density functional theory (DFT) calculations were conducted to determine the binding sites and energies in **CMOM-7**.^67^ The CH_4_ binding site was observed to have C–H···π interactions with a) the edges of bipy-1 pyridine rings (2.89–3.25 Å); and b) the SC bond of SCN^–^ (3.63 Å, Figure 5a), resulting in a CH_4_ binding energy of 14.2 kJ mol^–1^. The C_2_H_6_ binding site featured stronger interactions with **CMOM-7** through C–H···π interactions with a) bipy pyridine rings (3.46–3.86 Å) and b) the SC bonds of SCN^–^ ligands (3.93 Å, Figure 5b). The C_2_H_6_ binding energy was calculated to be 25.7 kJ mol^–1^. For C_3_H_8_, two binding sites were identified, where the second site revealed two orientations (Figures 5c,d and S31). Site-*I* interacts with the pyridine rings of bipy ligands through C–H···π interactions (3.24–3.99 Å, Figure 5c). Site-*II* interacts through the S atoms of SCN^–^ forming H-bonds (3.72 Å), and the benzene rings of *S*-IDEC (3.08–3.99 Å, Figure 5d). The C_3_H_8_ binding energies with **CMOM-7** in sites-*I* and *II* were determined to be 38.5 and 31.1 kJ mol^–1^, respectively. The modeled binding sites were found to be in agreement with site-*I* and part-B of site-*II* in the SCXRD determined crystal structure of **CMOM-7-C**_**3**_**H**_**8**_ (Figure 4). Moreover, the calculated binding energies fit the trend indicated by the experimental *Q*_st_ trends (Figure 2c).

In this work, a novel **lon** topology ultramicroporous MOF, **CMOM-7**, is reported. The ultramicroporosity and pore chemistry of **CMOM-7** was found to exhibit stronger affinity toward C_3_H_8_ vs both C_2_H_6_ and CH_4_, leading to high-purity C_3_H_8_ production from a ternary C_3_H_8_/C_2_H_6_/CH_4_ (v/v/v = 5:10:85) gas mixture. DCB experiments revealed high C_3_H_8_ uptake (2.45 mmol g^–1^), high C_3_H_8_/C_2_H_6_ selectivity (10.1) and a long breakthrough time difference, Δt (79.5 min g^–1^) between C_3_H_8_ and C_2_H_6_. SCXRD analysis of the propane binding sites in **CMOM-7** channels and complementary modeling studies of the C_3_H_8_ binding sites indicate that multiple weak intermolecular interactions are the key to the observed separation performance. That **CMOM-7** exhibited stronger intermolecular interactions with C_3_H_8_ molecules over C_2_H_6_ and CH_4_ is key to its high C_3_H_8_ selectivity even under ternary mixture compositions, in turn leading to the benchmark breakthrough time difference, Δ*t*. In this context, **CMOM-7** outperforms previously studied MOFs under equivalent experimental conditions. The approach taken herein, systematic crystal engineering of ultramicroporous coordination networks to fine-tune pore size, shape, and chemistry, suggests that more energy-efficient approaches to purification of commodity chemicals will ultimately come to fruition.

## Supporting Information Available

Experimental details, supplementary characterization results, additional figures, data analysis, and computational calculations (PDF)
AIF files (ZIP)
X-ray data for **CMOM-7**-C_3_H_8_ (CIF)
X-ray data for **CMOM-7**-DMF (CIF)
X-ray data for **CMOM-7**-H_2_O (CIF)
X-ray data for **CMOM-7**-MeOH (CIF)
X-ray data for **CMOM-7**-vac (CIF)

## Supplementary Material

tz3c01157_si_002.zip

tz3c01157_si_003.cif

tz3c01157_si_004.cif

tz3c01157_si_005.cif

tz3c01157_si_006.cif

tz3c01157_si_007.cif

## ABBREVIATIONS

NG: natural gas
CH_4_: methane
C_2_H_6_: ethane
C_2_H_4_: ethylene
C_2_H_2_: acetylene
C_3_H_8_: propane
C_3_H_6_: propylene
C3H4: methyl acetylene
SCXRD: single-crystal X-ray diffraction
MOMs: metal–organic materials
MOFs: metal–organic frameworks
PCPs: porous coordination polymers
PCNs: porous coordination networks
CMOMs: chiral metal–organic materials
*S*-IDEC: *S*-indoline-2-carboxylicate
RBB: rod building block
bipy: 4,4′-bipyridine
CCSs: chiral crystalline sponges
CSD: Cambridge, structural database
PXRD: powder X-ray diffraction
VT-PXRD: variable temperature powder X-ray diffraction
TGA: thermogravimetric analyses
DMF: N,N-dimethylformamide
SSLF: single-site Langmuir Freundlich
BET: Brunauer–Emmett–Teller
DSLF: Double-site Langmuir Freundlich
*Q*_st_: isosteric enthalpy of adsorption
IAST: ideal adsorption solution theory
DCB: dynamic column breakthrough
GC: gas chromatography
FID: flame ionization detector
TCD: thermal conductivity detector
Δt: breakthrough time difference
DVS: dynamic vapor sorption
H-bond: hydrogen bond
GCMC: Grand Canonical Monte Carlo
DFT: density field theory
