# Supplementary material for: Ultramicroporous Lonsdaleite Topology MOF with High Propane Uptake and Propane/Methane Selectivity for Propane Capture from Simulated Natural Gas
Source: ACS Mater Lett. 2023 Dec 1;6(1):56–65. doi: 10.1021/acsmaterialslett.3c01157 (PMC10762655; doi:10.1021/acsmaterialslett.3c01157)
Supplement: Supplementary file 2 — tz3c01157_si_002.zip [file tz3c01157_si_002.zip › Supporting Information for Publication/Supporting Information for Publication/Revised SI.docx]

**Ultramicroporous Lonsdaleite Topology MOF with High Propane Uptake and Propane/Methane Selectivity for Propane Capture from Simulated Natural Gas**

Chenghua Deng,† Li Zhao,‡ Mei-Yan Gao,† Shaza Darwish,† Bai-Qiao Song,† Debobroto Sensharma,† Matteo Lusi,† Yun-Lei Peng,*,‡ Soumya Mukherjee,*,† Michael J. Zaworotko*,†

†Bernal Institute, Department of Chemical Sciences, University of Limerick, Limerick V94 T9PX, Ireland.

‡Department of Applied Chemistry, College of Science, China University of Petroleum-Beijing, Beijing 102249, China.

Corresponding authors’ emails: [ylpeng@cup.edu.cn](mailto:ylpeng@cup.edu.cn); [soumya.mukherjee@ul.ie](mailto:soumya.mukherjee@ul.ie); [xtal@ul.ie](mailto:xtal@ul.ie)

**Table of Content**

[Materials and general experimental procedures 1](#_Toc148737047)

[Crystallographic analysis methods and tables 3](#_Toc148737048)

[Structural details 6](#_Toc148737049)

[Powder X-ray diffraction (PXRD) analysis 9](#_Toc148737050)

[Variable temperature powder X-ray diffraction (VT-PXRD) analysis 11](#_Toc148737051)

[Thermogravimetric analysis (TGA) 12](#_Toc148737052)

[Physical properties of methane, ethane, and propane 14](#_Toc148737053)

[Gas sorption measurements 15](#_Toc148737054)

[Dynamic column breakthrough (DCB) measurements 29](#_Toc148737055)

[CSD survey 34](#_Toc148737056)

[Water vapor sorption studies 36](#_Toc148737057)

[Comparison of gas sorption and separation performance 39](#_Toc148737058)

[Modelling studies 43](#_Toc148737059)

[References 45](#_Toc148737060)

Materials and general experimental procedures

Nickal (II) carbonate anhydrous (NiCO3, 98%) was purchased from Alfa Aesar. *S*-indoline-2-carboxylic acid (*S*-IDECH, 97.0%) was purchased from Fluorochem. Potassium thiocyanate (KSCN, ≥99.0%) was purchased from Sigma-Aldrich. *N*,*N*-dimethylformamide (DMF, HPLC grade, ≥ 99.9%), and methanol (HPLC grade, ≥ 99.9%) were purchased from Honeywell. Ethylene glycol (99%) was purchased from Thermo Scientific. All the gases, including methane (99.995%), ethane (99.95%), propane (99.5%), CO2 (99.999%), N2 (99.9992%), helium (99.996%) and hydrogen (99.9995%) were purchased from BOC Gases Ireland. Each was used directly without further purification.

Nickel (II) thiocyanate, Ni(SCN)2 was prepared as follows.1 First, NiCO3 powder (4.04 g, 14 mmol) was stirred in a 50 mL (0.56 mmol mL-1) methanol solution of KSCN for 12 h. The mixture was filtered, the solid was washed with methanol (10 mL × 3 times) and the filtrate was collected. The green solid of Ni(SCN)2 was obtained by evaporating the filtrate under reduced vacuum, using a rotary evaporator.

Synthesis of {[Ni(*S*-IDEC)(bipy)(SCN)](DMF)1.5}n (**CMOM-7-DMF**)

Ni(SCN)2 (53 mg, 0.3 mmol) was dissolved in 1.5 mL methanol, and 4,4'-bipyridine (bipy, 0.3 mmol, 47 mg) and S-indoline-2-carboxylic acid (50 mg, 0.3 mmol) was dissolved in 8.5 mL DMF. The two solutions were mixed in a vial, enclosed the cap, then move to the 85 °C oven for 20 h. Needle shaped blue crystals were obtained. Totally dried crystals were about 55 mg (0.25 mmol), about 12.6% yield based on Ni.

Preparing of {[Ni(*S*-IDEC)(bipy)(SCN)](MeOH)3}n (**CMOM-7-MeOH**)

Crystals of as-synthesized **CMOM-7** were soaked in 10 mL methanol for five days and the solvent were replaced by the flesh every day to completely remove DMF.

Preparing of {[Ni(*S*-IDEC)(bipy)(SCN)]}n (activated **CMOM-7**)

Methanol molecules in the channels of **CMOM-5-MeOH** were removed using high vacuum in Smart VacPrepTM at 60 °C for 12 h. The activated **CMOM-7** was prepared before gas sorption and gas separation studies.

Preparing of {[Ni(*S*-IDEC)(bipy)(SCN)](C3H8)1.5}n (**CMOM-7-C3H8**)

Put some activated crystals of activated **CMOM-7** in a Schlenk flask with C3H8 atmosphere. And soak the Schlenk flask in the dry ice bath for 12 h.

Preparing of {[Ni(*S*-IDEC)(bipy)(SCN)](H2O)6.5}n (**CMOM-7-H2O**)

Soaking crystals of **CMOM-7-MeOH** in deionized water for 12 h. The crystals keep the crystalline appearance (Figure S1), and SCXRD demonstrated that water molecules in **CMOM-7-H2O** occupied the whole void volume.


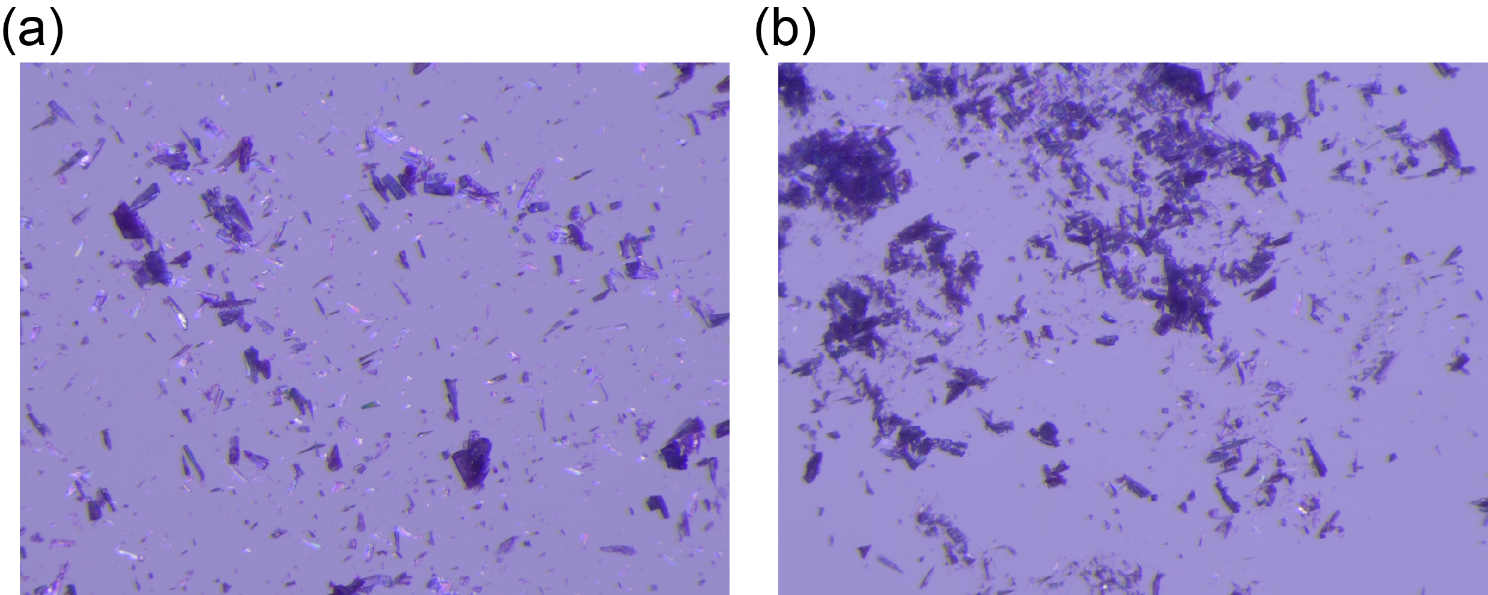


**Figure S1**. Microscopic images of the crystals for **CMOM-7-MeOH** (a), and **CMOM-7-H2O** (b).

Crystallographic analysis methods and tables

Suitable single crystals of all compounds were chosen for single crystal X-ray diffraction measurements. All crystallographic data were collected on a Bruker D8 Quest diffractometer equipped with a I*μ*S micro-focus Cu anode Cu Kα (λ = 1.54178 Å) and Photon II detector. For low temperature measurements, an open-flow nitrogen attachment from Oxford Cryosystems was used. The data was indexed in APEX4 (v2021.10-0). Integration was performed by SAINT V8.40A in APEX4. Absorption correction was performed by multi-scan method using SADABS in APEX4.2 Space group determinations were performed with the assistance of XPREP, as implemented in APEX4.

Structures were solved using the intrinsic phasing method (SHELXT) and refined on F2 using SHELXL least squares method as run in OLEX2 v1.3 program packages.3-5 All non-hydrogen atoms on the frameworks were refined anisotropically. Hydrogen atoms were added geometrically at idealized positions and refined using the riding model. The thermal parameters and geometry of the guest molecules were restricted to reasonable limits using the ISOR, SIMU, DFIX, and FLAT instructions. Crystallographic data for all compounds are summarized in the following crystallographic tables S1 and S2. Further, SQUEEZE subroutine of the PLATON software suite was used to remove scattering from the highly disordered guest molecules.6 All crystal structures were deposited to the Cambridge Crystallographic Data Centre (CCDC: 2285983 to 2285987).

**Table S1**. Crystallographic data of **CMOM-7-DMF** and **CMOM-7-MeOH**.

|  | **CMOM-7-DMF** | **CMOM-7-MeOH** |
| --- | --- | --- |
| Formula | C49H53N11Ni2O7S2 | C46H56N8Ni2O10S2 |
| Formula weight | 1089.56 | 1062.52 |
| Temperature/K | 150.0 | 150.0 |
| Crystal system | orthorhombic | orthorhombic |
| Space group | *P212121* | *P212121* |
| *a*/Å | 9.7163(5) | 9.7720(2) |
| *b*/Å | 22.7667(12) | 22.6651(5) |
| *c*/Å | 24.2743(11) | 24.3014(5) |
| *α*/° | 90 | 90 |
| *β*/° | 90 | 90 |
| *γ*/° | 90 | 90 |
| Volume/Å3 | 5369.7(5) | 5382.4(2) |
| Z | 4 | 4 |
| ρcalcg/cm3 | 1.348 | 1.311 |
| *μ*/mm-1 | 2.081 | 2.085 |
| F(000) | 2272.0 | 2224.0 |
| Crystal size/mm3 | 0.248 × 0.103 × 0.084 | 0.259 × 0.163 × 0.125 |
| Radiation | CuKα (λ = 1.54178) | CuKα (λ = 1.54178) |
| 2θ range for data collection/° | 5.322 to 133.492 | 5.332 to 133.228 |
| Index ranges | -10 ≤ h ≤ 11, -27 ≤ k ≤ 22, -28 ≤ l ≤ 23 | -11 ≤ h ≤ 11, -26 ≤ k ≤ 26, -28 ≤ l ≤ 28 |
| Reflections Collected | 53069 | 47967 |
| Independent reflections | 9510 [Rint = 0.1475, Rsigma = 0.0917] | 9483 [Rint = 0.0827, Rsigma = 0.0558] |
| Data / restraints / parameters | 9510/0/656 | 9483/67/666 |
| Goodness-of-fit on F2 | 1.001 | 1.045 |
| Final R indexes [I>=2σ(I)] | R1 = 0.0569, wR2 = 0.1352 | R1 = 0.0577, wR2 = 0.1556 |
| Final R indexes [all data] | R1 = 0.1087, wR2 = 0.1705 | R1 = 0.0650, wR2 = 0.1637 |
| Largest diff. peak/hole / e Å-3 | 0.51/-0.40 | 0.62/-0.58 |
| Flack parameter | 0.01(2) | 0.071(14) |
| CCDC number | 2285985 | 2285984 |

**Table S2**. Crystallographic data of activated **CMOM-7**, **CMOM-7-C3H8** and **CMOM-7-H2O**.

|  | activated **CMOM-7** | **CMOM-7-C3H8** | **CMOM-7-H2O** |
| --- | --- | --- | --- |
| Formula | C40H32N8Ni2O4S2 | C49H56N8Ni2O4S2 | C40H58N8Ni2O17S2 |
| Formula weight | 870.27 | 1002.55 | 1104.48 |
| Temperature/K | 150.0 | 150.0 | 150.0 |
| Crystal system | orthorhombic | orthorhombic | orthorhombic |
| Space group | *P212121* | *C2221* | *C2221* |
| *a*/Å | 9.7963(2) | 24.5233(10) | 9.68340(10) |
| *b*/Å | 22.3383(5) | 9.6304(4) | 24.2453(3) |
| *c*/Å | 24.1559(5) | 22.8429(9) | 22.7180(3) |
| *α*/° | 90 | 90 | 90 |
| *β*/° | 90 | 90 | 90 |
| *γ*/° | 90 | 90 | 90 |
| Volume/Å3 | 5286.10(19) | 5394.8(4) | 5333.66(11) |
| Z | 4 | 4 | 4 |
| ρcalcg/cm3 | 1.094 | 1.234 | 1.375 |
| *μ*/mm-1 | 1.943 | 1.965 | 2.223 |
| F(000) | 1792.0 | 2104.0 | 2312.0 |
| Crystal size/mm3 | 0.162 × 0.087 × 0.055 | 0.198 × 0.114 × 0.082 | 0.116 × 0.074 × 0.036 |
| radiation | CuKα (λ = 1.54178) | CuKα (λ = 1.54178) | CuKα (λ = 1.54178) |
| 2θ range for data collection/° | 5.388 to 136.536 | 7.21 to 137.218 | 8.266 to 144.888 |
| Index ranges | -11 ≤ h ≤ 11, -26 ≤ k ≤ 26, -29 ≤ l ≤ 28 | -29 ≤ h ≤ 28, -11 ≤ k ≤ 11, -27 ≤ l ≤ 27 | -11 ≤ h ≤ 9, -29 ≤ k ≤ 29, -28 ≤ l ≤ 26 |
| Reflections Collected | 54547 | 27442 | 22263 |
| Independent reflections | 9571 [Rint = 0.1358, Rsigma = 0.0855] | 4862 [Rint = 0.1281, Rsigma = 0.0793] | 5232 [Rint = 0.0388, Rsigma = 0.0313] |
| Data / restraints / parameters | 9751/0/505 | 4862/115/342 | 5232/8/337 |
| Goodness-of-fit on F2 | 1.004 | 1.022 | 1.093 |
| Final R indexes [I>=2σ(I)] | R1 = 0.0564, wR2 = 0.1427 | R1 = 0.0556, wR2 = 0.1351 | R1 = 0.0407, wR2 = 0.1118 |
| Final R indexes [all data] | R1 = 0.1027, wR2 = 0.1717 | R1 = 0.0709, wR2 = 0.1493 | R1 = 0.0431, wR2 = 0.1143 |
| Largest diff. peak/hole / e Å-3 | 0.64/-0.39 | 0.38/-0.49 | 0.96/-0.35 |
| Flack parameter | 0.060(19) | -0.07(5) | 0.034(9) |
| CCDC number | 2285983 | 2285987 | 2285986 |

Structural details


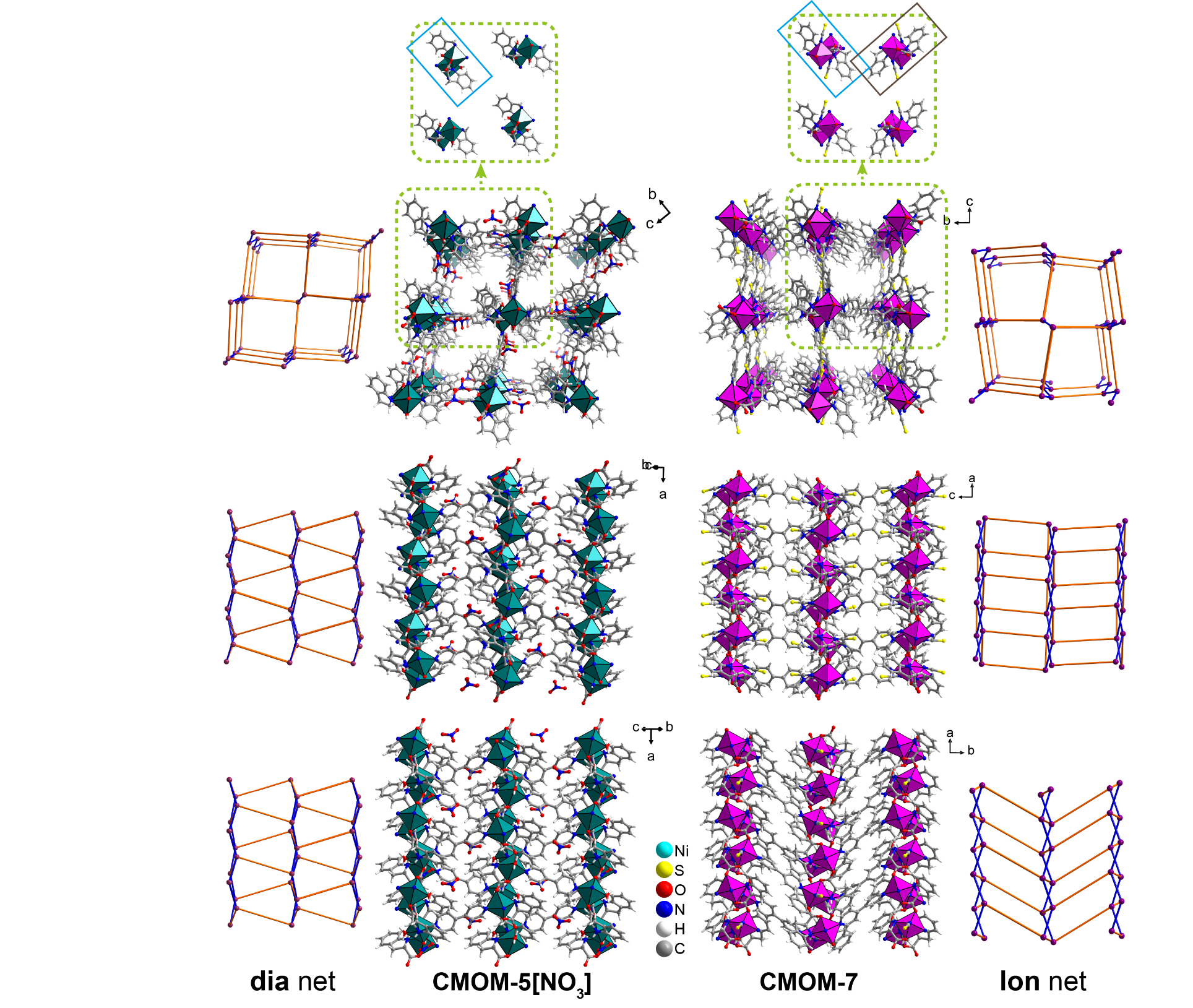


**Figure S2.** Crystal structures of **CMOM-5**, **CMOM-7** and the corresponding **dia** and **lon** nets. Solvent molecules were omitted for clarity. The prolong direction of RBBs were shown on the top, labelled by the rectangles.


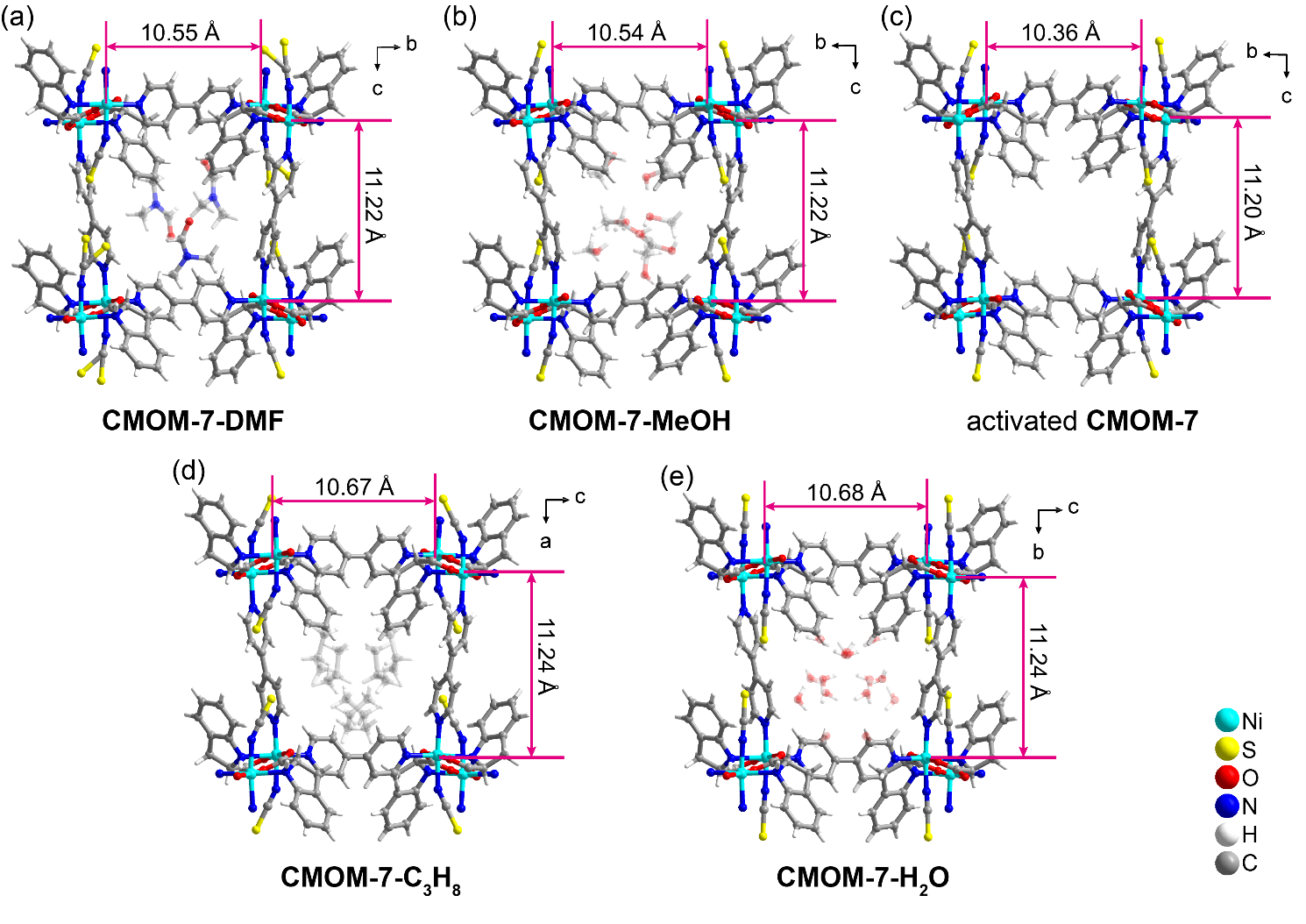


**Figure S3.** Crystal structures of **CMOM-7** with different guest molecule loaded. From (a) to (e) are corresponding to **CMOM-7-DMF**, **CMOM-7-MeOH**, activated **CMOM-7**, **CMOM-7-C3H8**, **CMOM-7-H2O**. The shortest distance between the nickel cations of the adjacent RBBs were labelled. The atoms and bonds of guest molecules were drawn in 80% transparency for clarity.


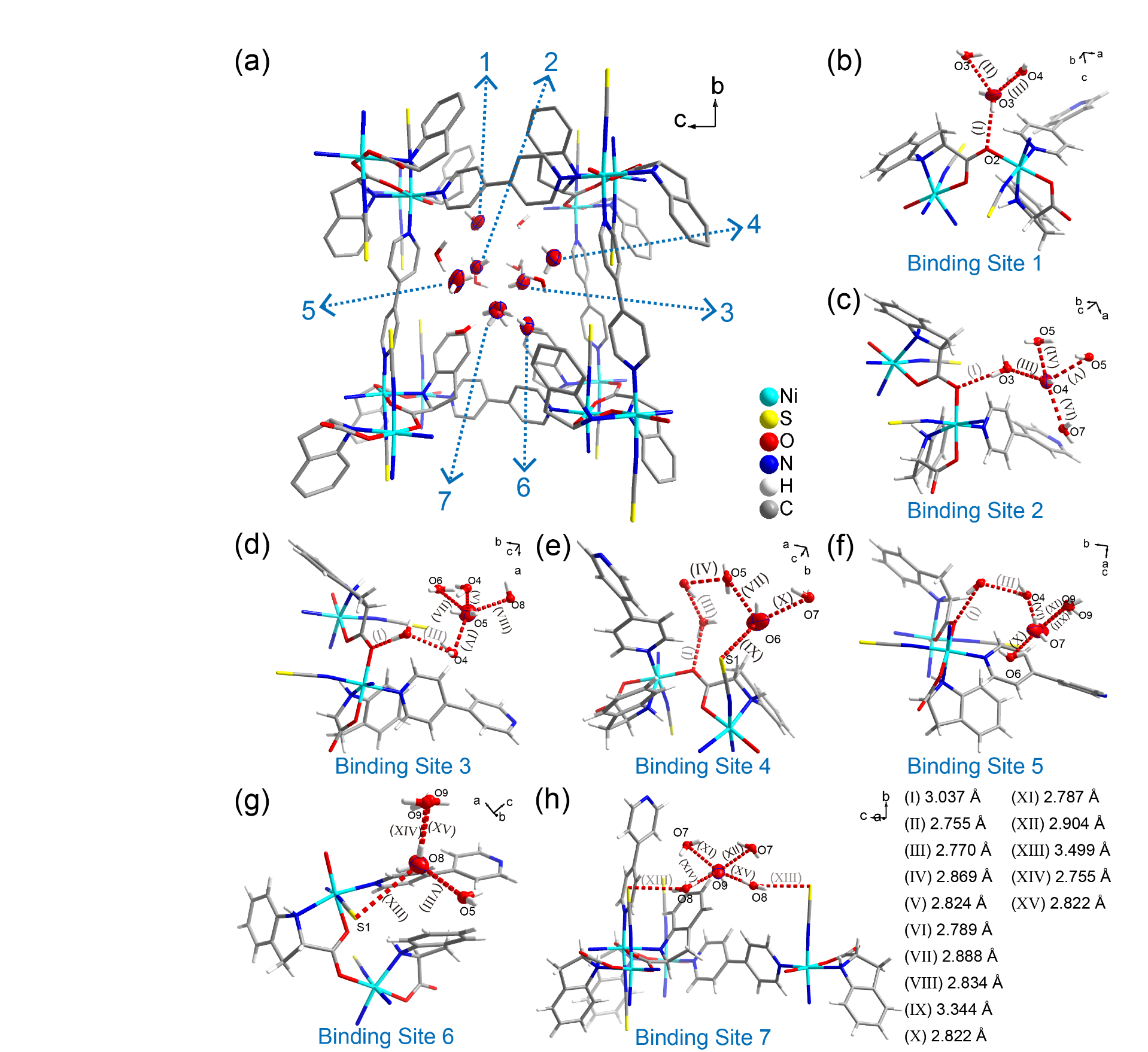


**Figure S4.** The positions of the binding sites of water molecules in **CMOM-7-H2O** (a). And the intermolecular interactions around the water molecules in each binding site (b)-(h).

Powder X-ray diffraction (PXRD) analysis

Powder X-ray Diffraction (PXRD) patterns were recorded at room temperature on a Panalytical Empyrean (Cu Kα, λ = 1.5418 Å) with a 1D PIXcel strip detector. Experiments were conducted in continuous scanning mode with the goniometer placed in the theta-theta orientation. Incident beam optics included the fixed divergences slit with anti-scatter slit PreFIX module, with a 1/8° divergence slit and a 1/4° anti-scatter slit, as well as a 10 mm fixed incident beam mask and a Soller slit (0.04 rad). Divergent beam optics included a P7.5 anti-scatter slit, a Soller slit (0.04 rad), and a Ni β filter. The data were collected in the range of 2θ = 3-50°. Raw data was then evaluated using the X'Pert HighScore Plus™ software V4.1 (PANalytical, The Netherlands).


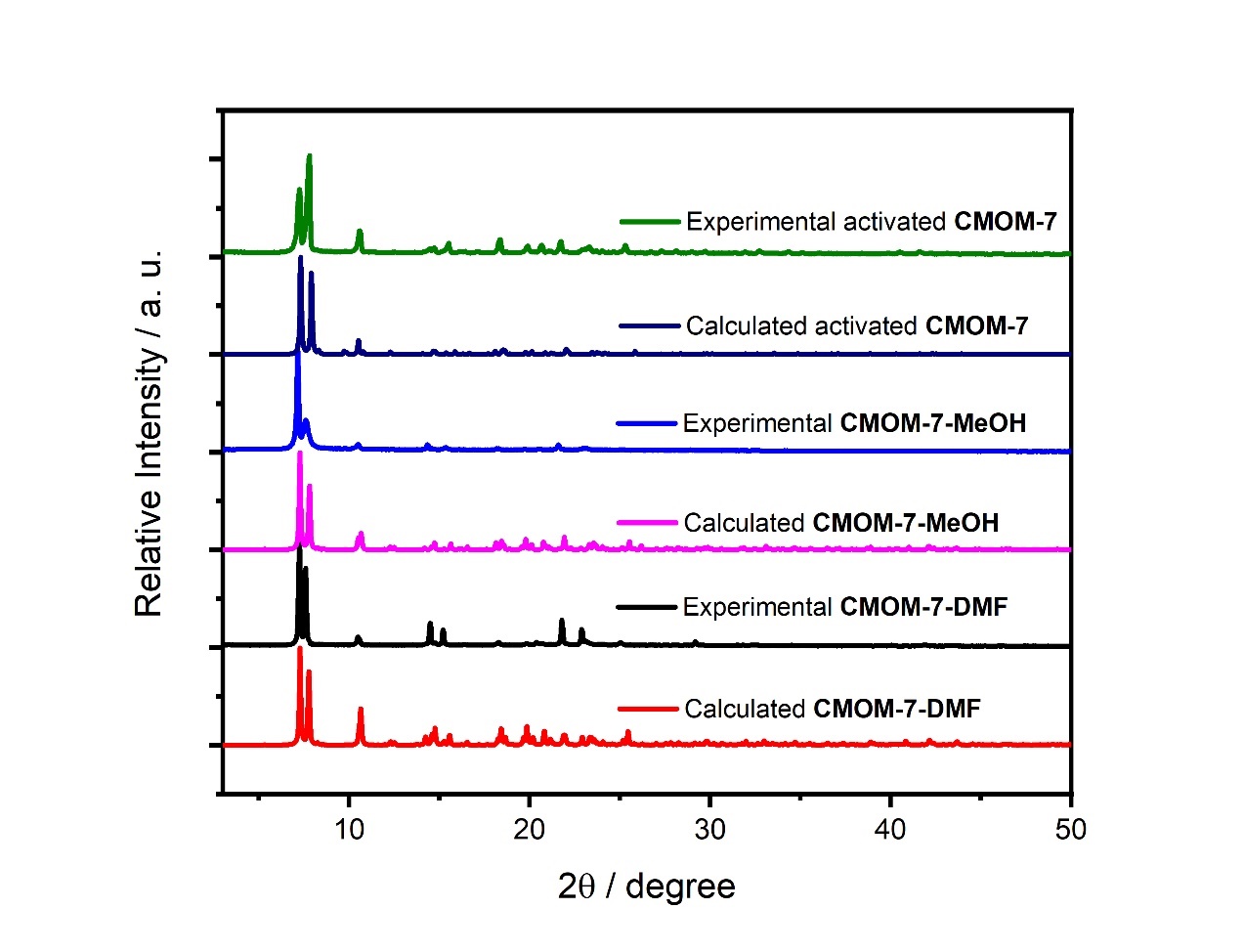


**Figure S5**. PXRD patterns of **CMOM-7-DMF**, **CMOM-7-MeOH** and activated **CMOM-7**.


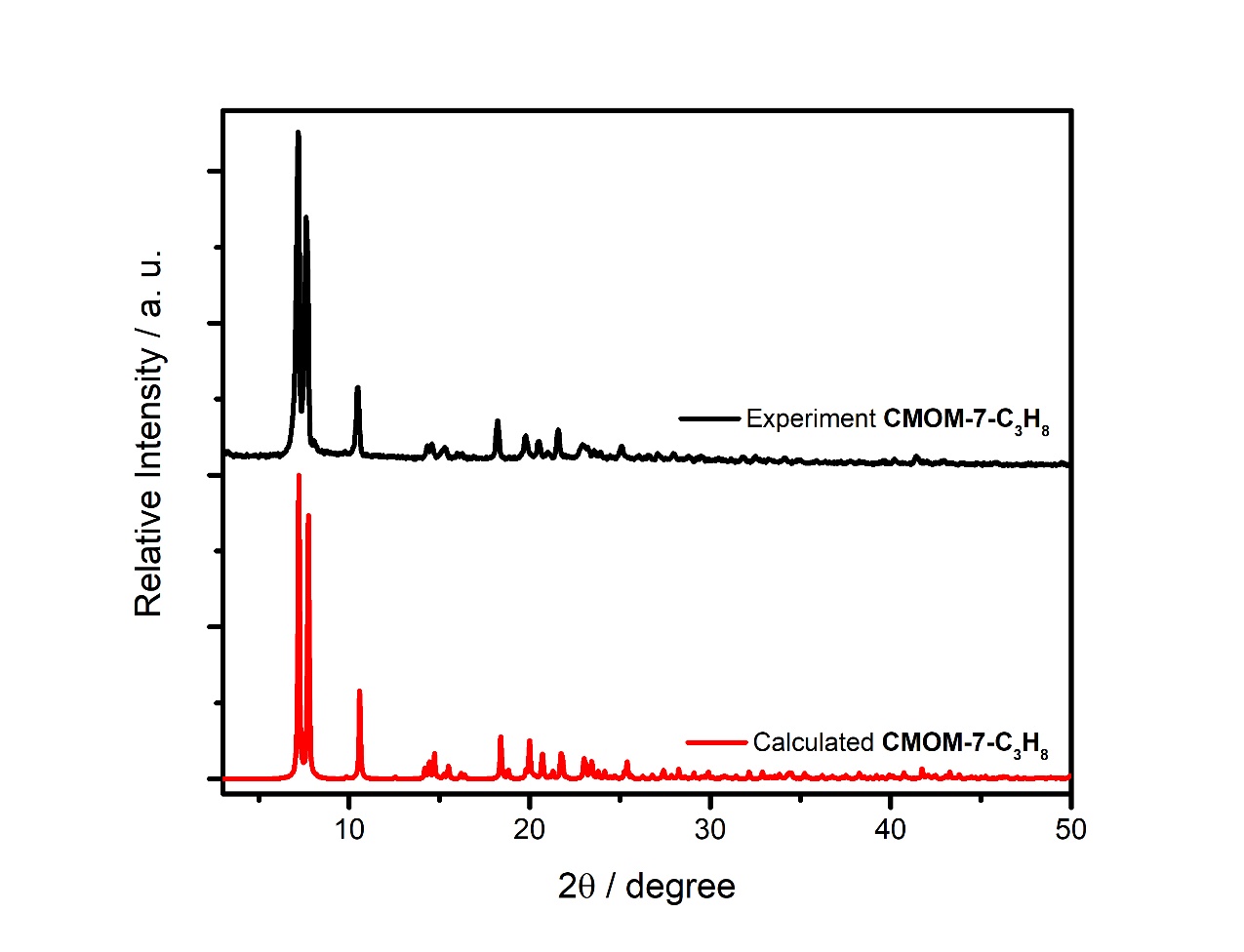


**Figure S6**. PXRD patterns of **CMOM-7-C3H8**.


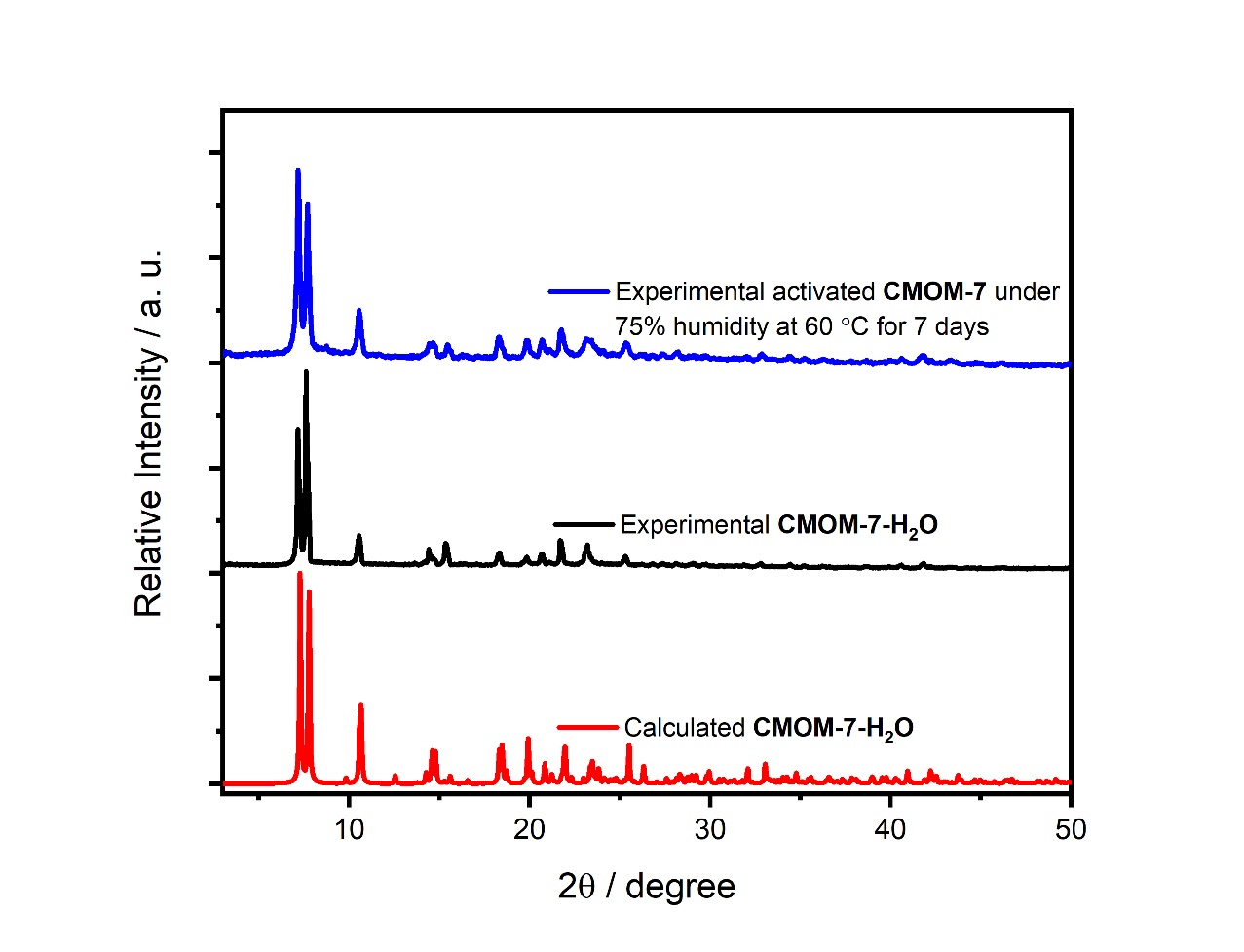


**Figure S7**. PXRD patterns ofa) **CMOM-7-H2O** calculated *vs*. experimental, plotted alongside that of the activated **CMOM-7** after keeping under 75% RH at 60 °C for 7 days.

Variable temperature powder X-ray diffraction (VT-PXRD) analysis

Diffractograms at different temperatures were recorded using a PANalytical X'Pert Pro-MPD diffractometer equipped with a PIXcel3D detector operating in scanning line detector mode with an active length of 4 utilizing 255 channels. The Anton Paar TTK 450 stage coupled with the Anton Paar TCU 110 temperature control unit was used to record the variable temperature diffractograms. The diffractometer is outfitted with an Empyrean Cu LFF (long fine-focus) HR (9430 033 7300x) tube operated at 40 kV and 40 mA and CuKα radiation (λα = 1.54056 Å) was used for diffraction experiments. Continuous scanning mode with the goniometer in the theta-theta orientation was used to collect the data. Incident beam optics included the Fixed Divergences slit, with a 1/4° divergence slit and a Soller slit (0.04 rad). Divergent beam optics included a P7.5 anti-scatter slit, a Soller slit (0.04 rad), and a Ni-β filter. In a typical experiment, 15 mg of sample was ground into a fine powder, and was loaded on a zero background sample holder made for Anton Paar TTK 450 chamber. The data was collected from 4°-40° (2θ) with a step-size of 0.0167113° and a scan time of 50 seconds per step. Crude data were analyzed using the X'Pert HighScore Plus™ software V 4.1 (PANalytical, The Netherlands). Each sample was heated up to 250 oC under N2 atmosphere.


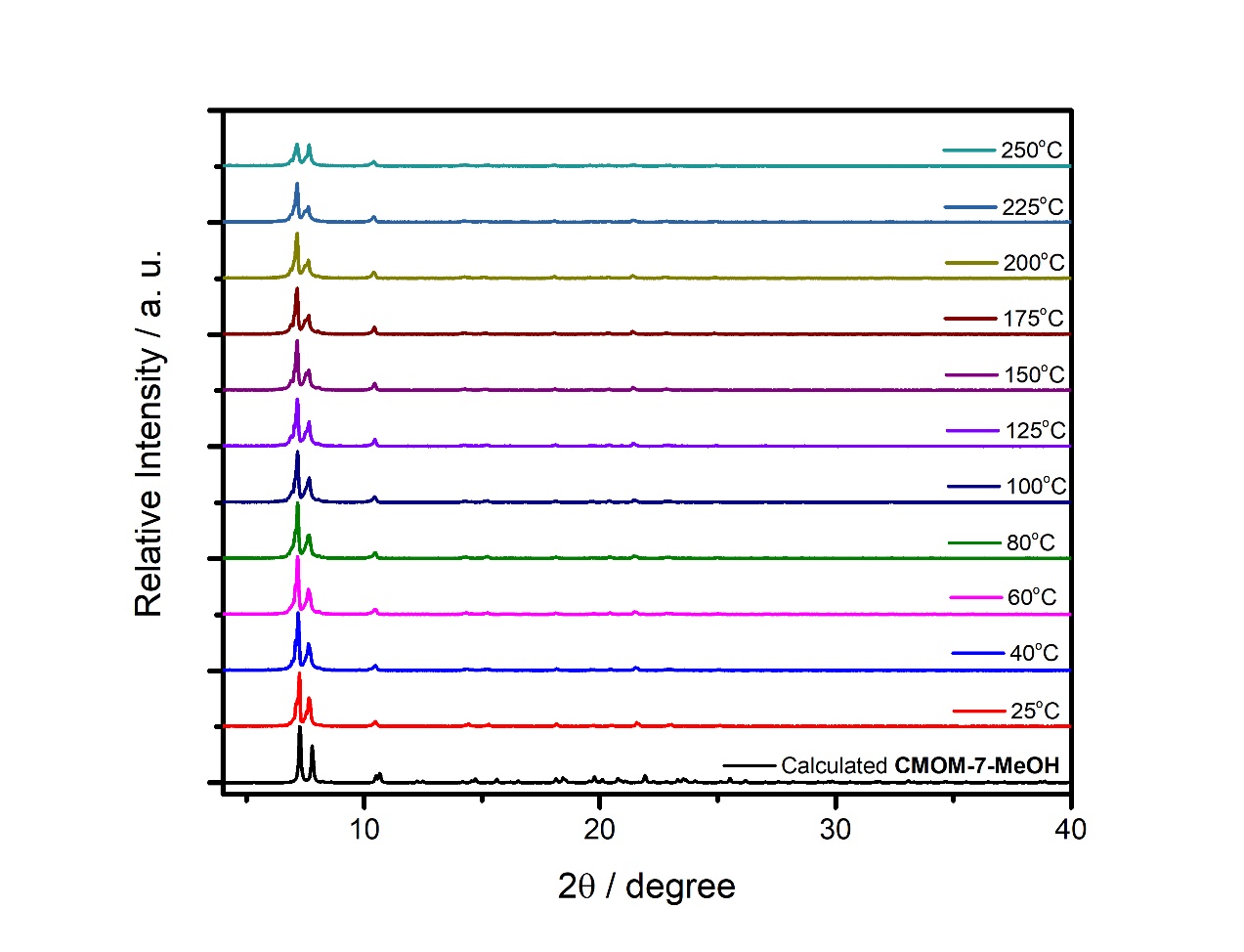


**Figure S8**. The VT-PXRD patterns of **CMOM-7-MeOH** under nitrogen flow.

Thermogravimetric analysis (TGA)

Thermogravimetric analyses (TGA) were performed under N2 (60 mL per minute) using a TA Instruments Q50 system. Samples were loaded into aluminium sample pans and heated at 10 °C min-1 from room temperature to 550 °C.


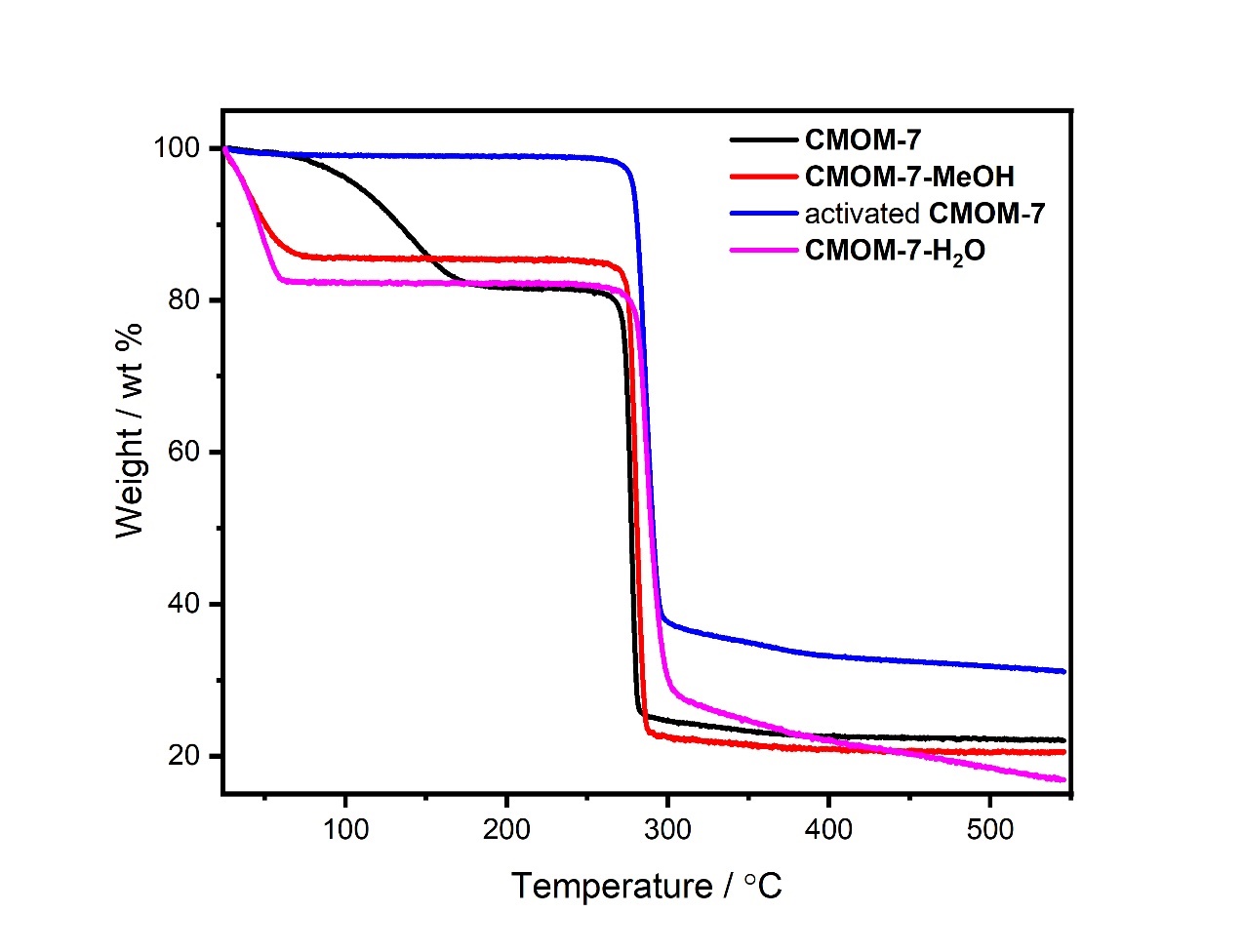


**Figure S9**. The TGA traces of **CMOM-7**, **CMOM-7-MeOH**, activated **CMOM-7** and **CMOM-7-H2O**.

Gravimetric kinetic adsorption isotherms were recorded under pure 10 cm3 min-1 CH4, C2H6, and C3H8 flows using a TA Instruments Q50 system. Gas flows were controlled by pre-callibrated Bronkhorst Mass Flow Controllers. Before the three gravimetric kinetic adsorption cycles, the crystals of **CMOM-7-MeOH** were exposed to 60 mL min-1 hydrocarbon flow for 20 minutes at room temperature, and then, the crystals were activated under 60 mL min-1 N2 flow for 30 minutes at room temperature. For each cycle, the temperature was ramped up to 80 °C (20 °C min-1) and the crystals were activated under 60 mL min-1 N2 flow at 80 °C for 30 minutes, then the temperature was ramped down to 30 °C (20 °C min-1) and the samples were balanced under 60 mL min-1 N2 flow for 30 minutes at 30 °C. After the activation and equilibrium, the sample was exposed to the 10 cm3 min-1 flows of CH4, C2H6, and C3H8 for 90 minutes, each. The kinetic adsorption profiles were evaluated using the T.A. Universal Analysis software.


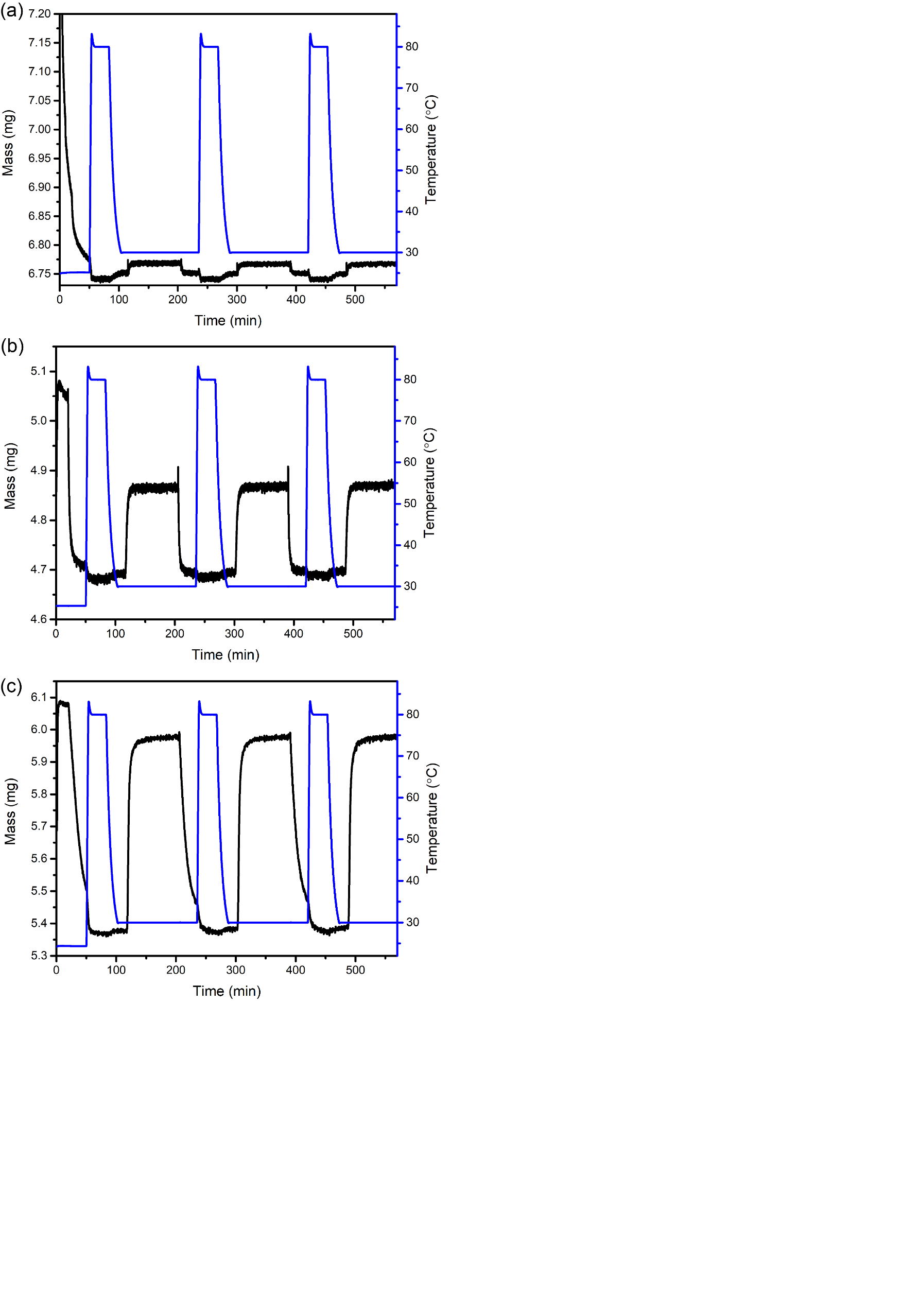


**Figure S10**. Single-component gravimetric kinetics sorption cycling experiments for **CMOM-7** between 303 K to 363 K under (a) CH4 flow, (b) C2H6 flow, and (c) C3H8 flow.

Physical properties of methane, ethane, and propane

**Table S3.** The key physical properties of methane, ethane and propane.7

|  | Methane | Ethane | Propane |
| --- | --- | --- | --- |
| Formula | CH4 | C2H6 | C3H8 |
| Molar Mass (g mol-1) | 16.04 | 30.07 | 44.10 |
| Boiling Point (K) | 111.6 | 184.6 | 231.05 |
| Dynamic Diameter (Å) | 3.76 | 4.44 | 4.3 |
| Molecular Size (Å) | 3.83 × 3.94 × 4.10 | 3.81 × 4.08 × 4.82 | 4.20 × 4.60 × 6.80 |
| Polarizability (× 10-25 cm3) | 25.93 | 44.3-44.7 | 62.9-63.7 |

Gas sorption measurements

The methanol exchanged crystals (**CMOM-7-MeOH**) were activated under high vacuum by a Micromeritics® Smart VacPrepTM at 60 °C for 12 h (activated **CMOM-7**) before gas sorption studies. The sorption isotherms for N2 at 77 K and CO2 at 195 K were measured by a Micromeritics® TriStar II Plus surface area and porosity analyzer. The 195 K environment was controlled by a 2.75 L dewar containing the bath of acetone and dry ice. The 77 K environment was controlled by a 2.75 L dewar of liquid nitrogen. The sorption isotherms for CH4, C2H6 and C3H8 were measured by a Micromeritics® 3Flex adsorption analyzer, and the temperature was controlled by a Julabo temperature controller with the ethylene glycol/water = 1:1 (v/v) mixture liquid. High pressure CO2 isotherm at 298 K was measured by a Hiden Isochema Xemis-002 instrument.


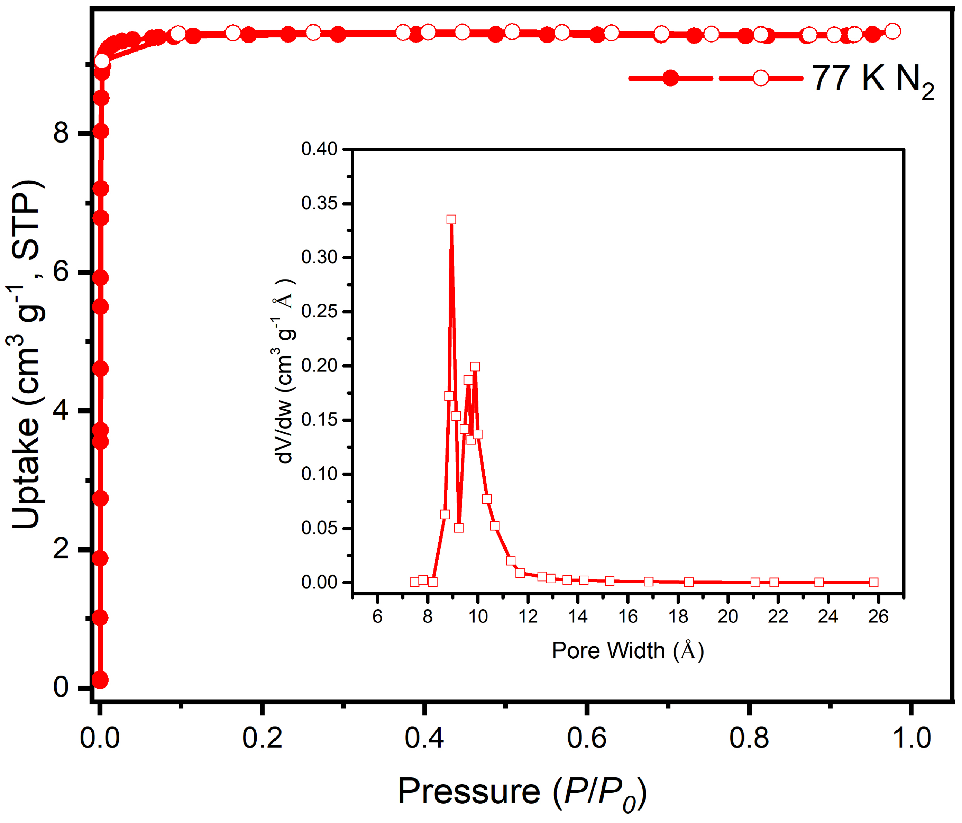


**Figure S11**. 77 K N2 sorption isotherm of **CMOM-7** (closed and open symbols denote adsorption and desorption, respectively) and the corresponding H-K pore size distribution profile (inset).


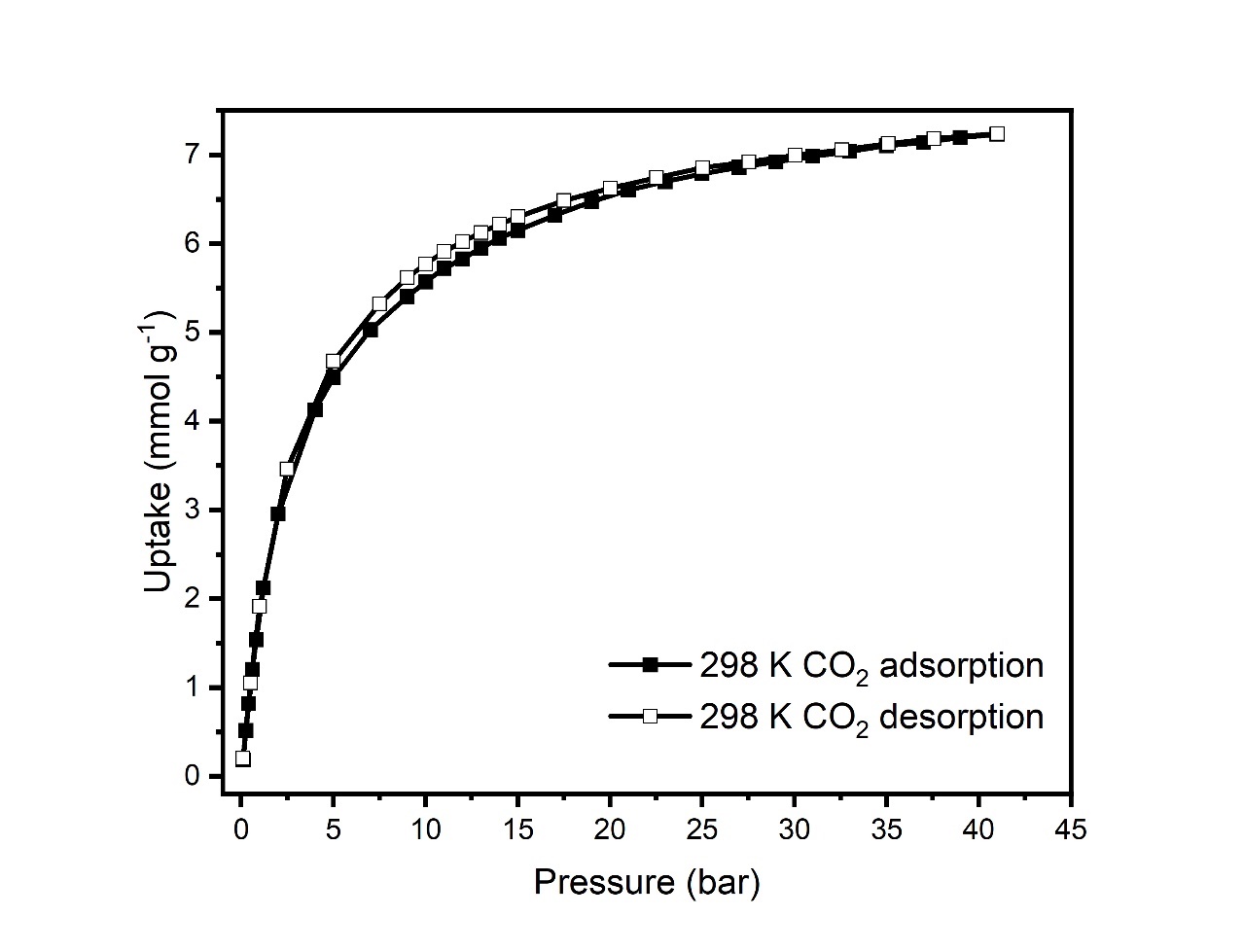


**Figure S12**. 298 K high-pressure CO2 sorption isotherm of **CMOM-7** (closed and open symbols denote adsorption and desorption, respectively).


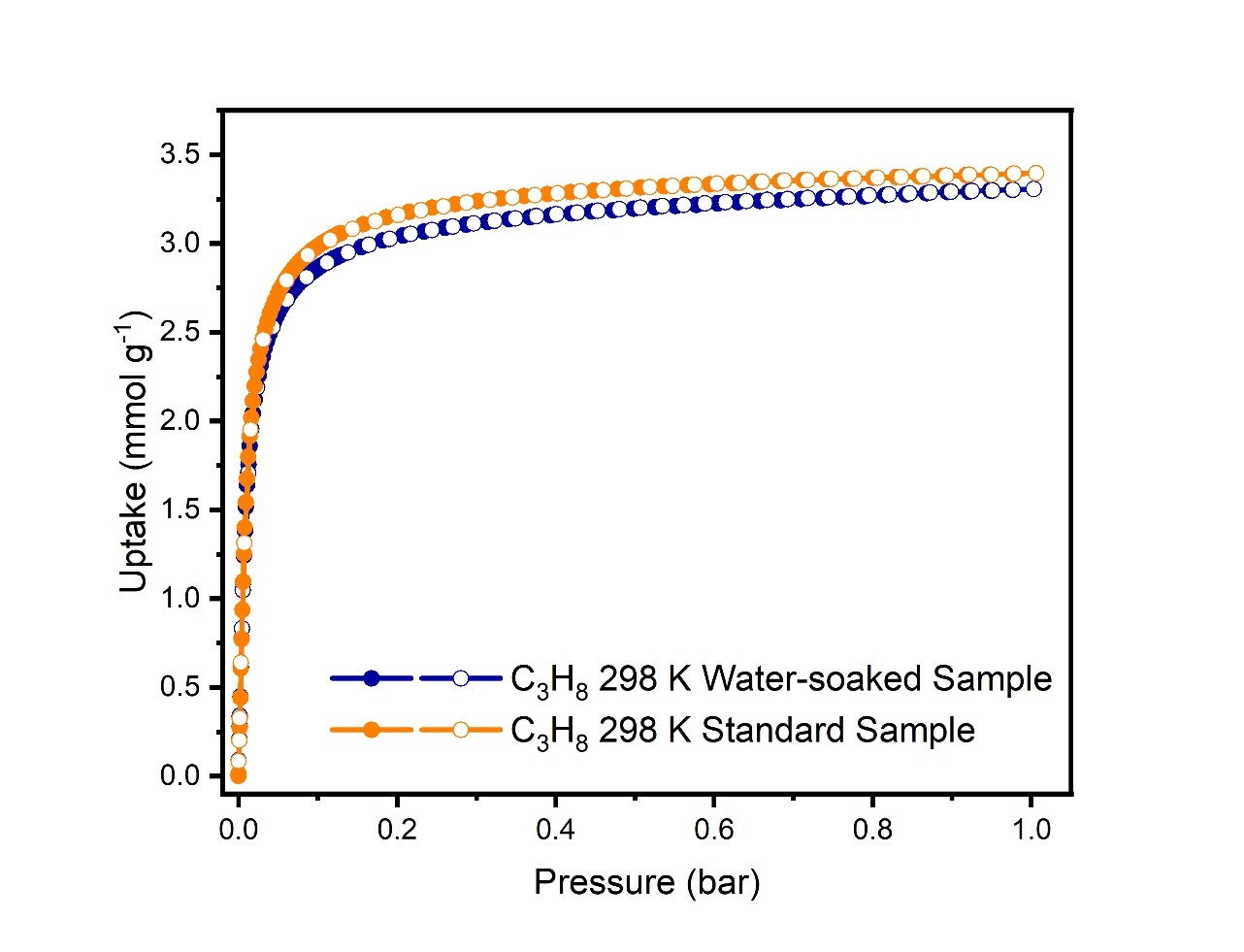


**Figure S13**. 298 K C3H8 sorption isotherms of the standard sample and water-soaked sample of **CMOM-7** (closed and open symbols denote adsorption and desorption, respectively).

The Langmuir isotherm model can be expressed by the following equation8-10:


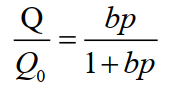


where Q (cm-3 g-1) is the amount adsorbed; Q0 (cm-3 g-1) is the saturated amount adsorbed; p (mmHg) is the equilibrium pressure; and b (mmHg-1) is the adsorption affinity. A line equation for the Langmuir equation can be written as following:


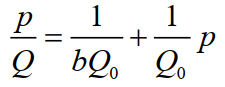


A least-squares fitting is performed on the () designated pairs where is the independent variable and p is the dependent variable. The following are calculated: a) Slope (, g cm-3, STP); b) Y-intercept (, g mmHg cm-3, STP); c) Error of the slope (g cm-3, STP); d) Error of the y-intercept (g mmHg cm-3, STP).

Using the results of the above calculations, the Langmuir surface area can be calculated as following:


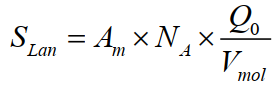


where *SLan* is the Langmuir surface area (m2 g-1); *Am* is the molecular cross-sectional area (nm2, 1 nm2 = 1 × 10-18 m2) of adsorbate (*i. e.* 0.1700 nm2 for CO2 and 0.1540 nm2 for N2); *Vmol* represents the molar volume of a gas at STP (22414 cm3 mol-1), and *NA* is the Avogadro’s number (6.02 × 1023 molecules/mol).


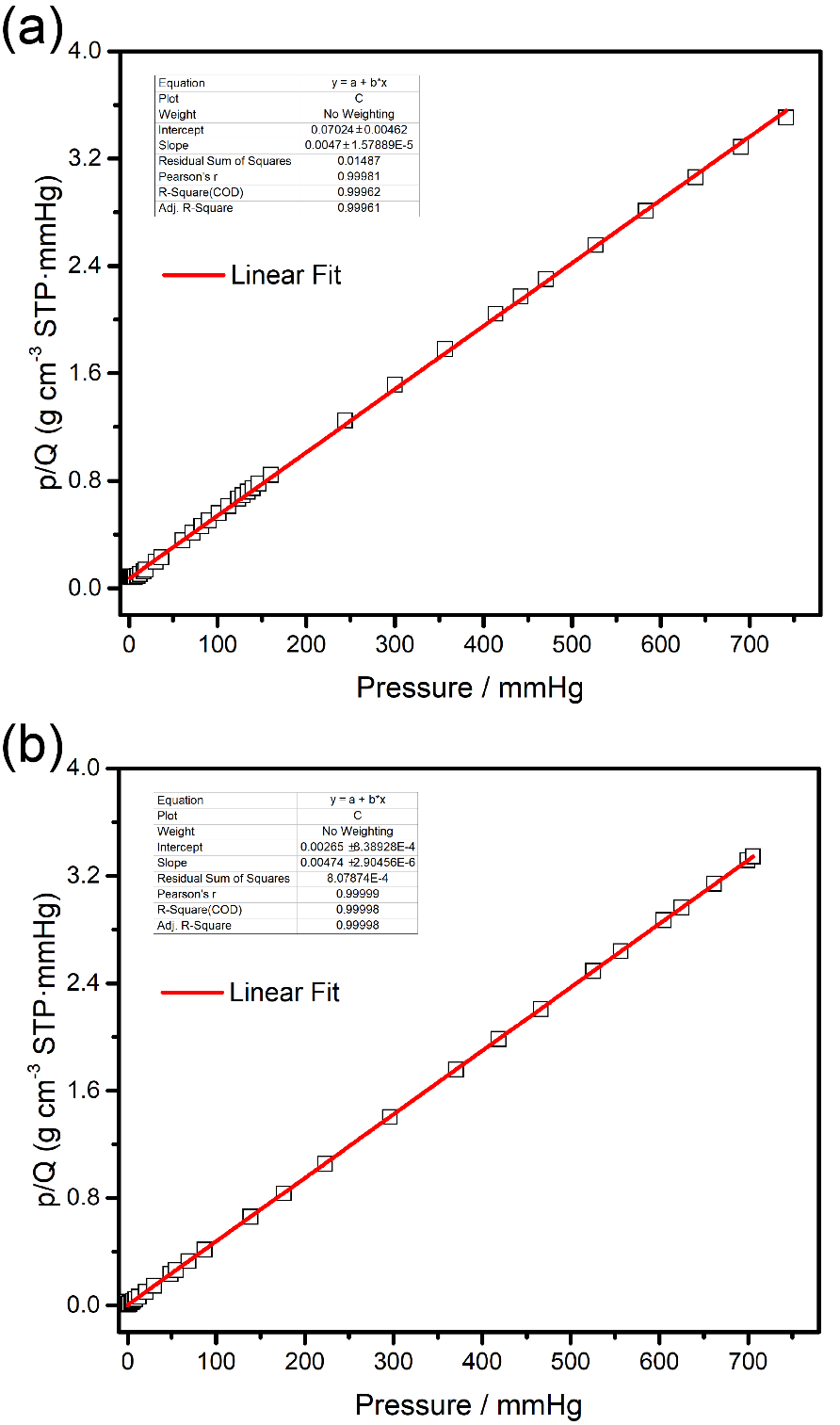


**Figure S14**. Langmuir fits from the 195 K CO2 (a) and 77 K N2 data (b). Squares- experimental data and line- fit for **CMOM-7**.

The BET (Brunauer–Emmett–Teller) isotherm model can be expressed by the following equation11:


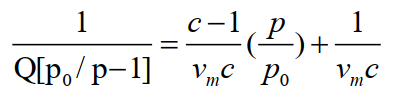


where *Q* (cm-3 g-1) is the amount adsorbed; *p* and *p0* (mmHg) are the equilibrium and the saturation pressure of adsorbates at the temperature of adsorption respectively; *vm* is the monolayer adsorbed gas quantity and *c* is the BET constant. The experimental isotherm can be plotted as a straight line with 1/(*Q*/*p0*/*p*-1) on the y-axis and *p*/*p0* on the x-axis. Based on the slope (*A*) and y-intercept (*I*) of the line, the value of *vm* and *c* could be expressed by the following equations:


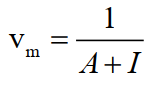


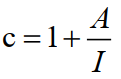


The BET-specific surface area is given by the following equations:


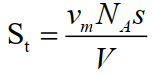


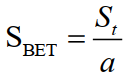


where *St* is the total surface area of the sample; *NA* is the Avogadro’s number (6.02 × 1023 molecules/mol); *s* is the molecular cross-sectional area (nm2, 1 nm2 = 1 × 10-18 m2) of adsorbate (*i. e.* 0.1700 nm2 for CO2 and 0.1540 nm2 for N2); *V* is the molar volume of adsorbed gas (22414 cm3 mol-1 at STP); *SBET* is the BET specific surface area and *a* is the mass of the sample.


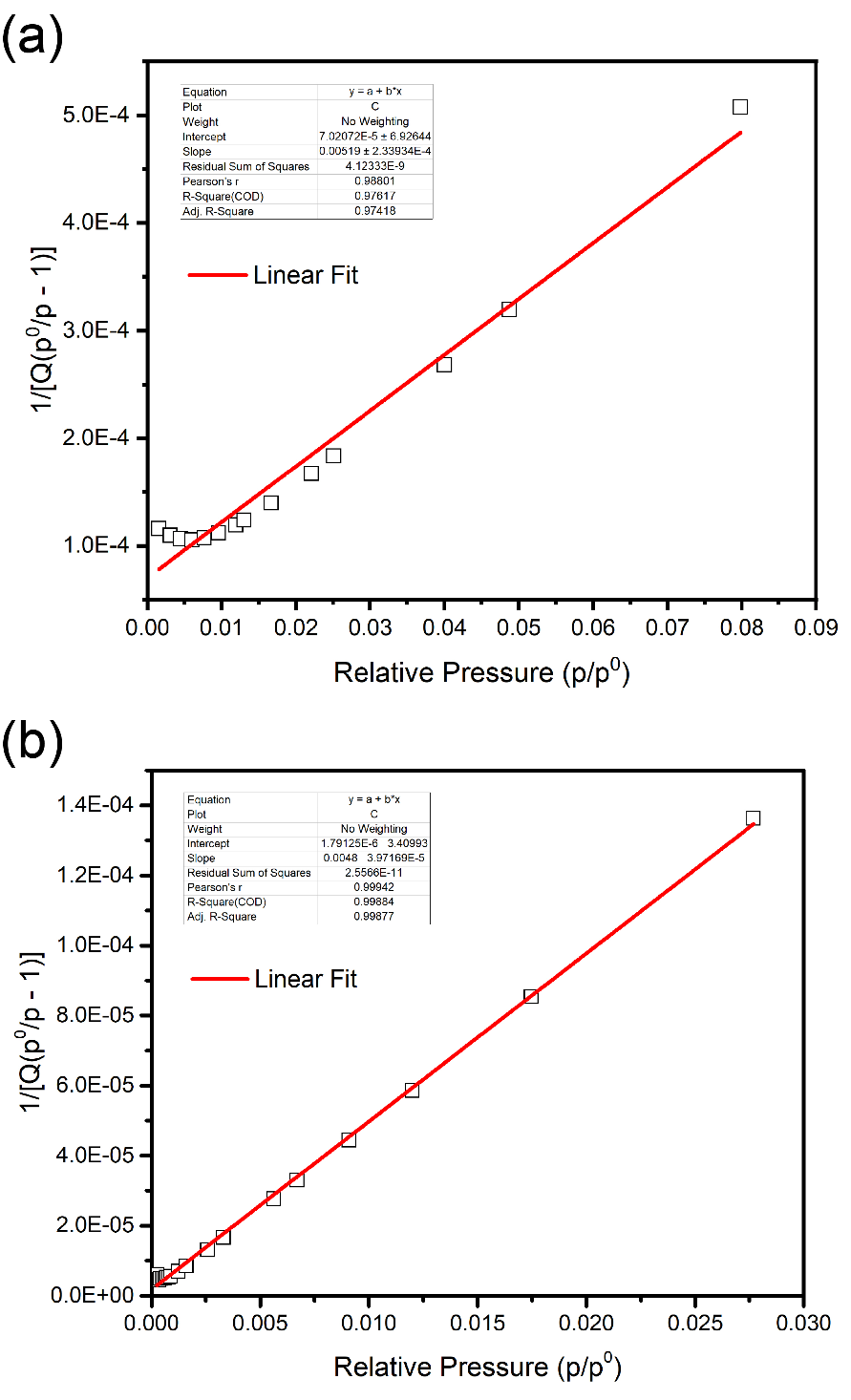


**Figure S15**. BET fits from the 195 K CO2 (a) and 77 K N2 data (b). Squares: experimental data; line: linear fit for **CMOM-7**.


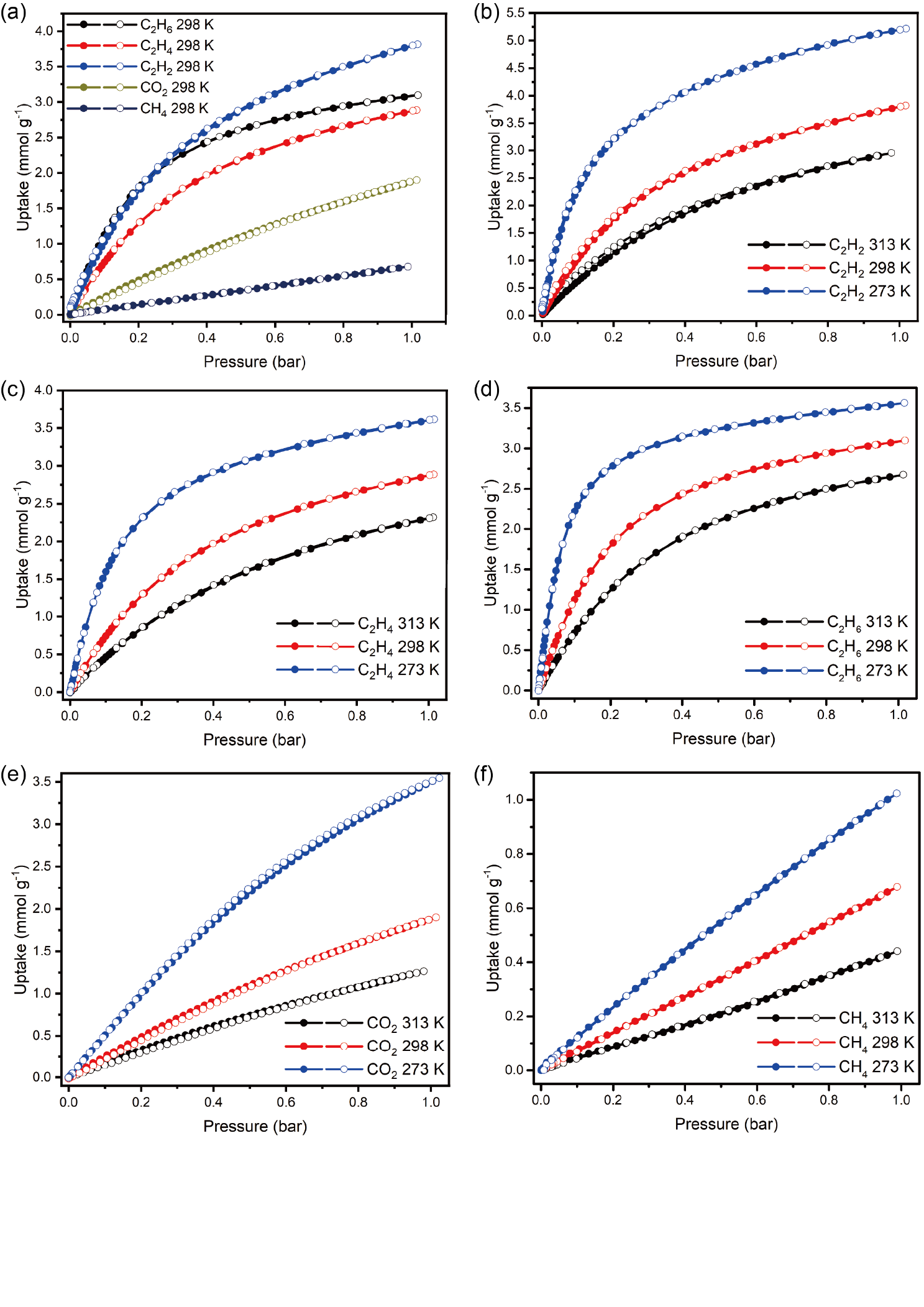


**Figure S16**. The comparison of C2H2, C2H4, C2H6, CO2, and CH4 sorption isotherms for **CMOM-7** at 298 K (a); C2H2 (b), C2H4 (c), C2H6 (d), CO2 (e) and CH4 (f) sorption isotherms of **CMOM-7** at 273 K, 298 K and 313 K (closed and open symbols denote adsorption and desorption, respectively).


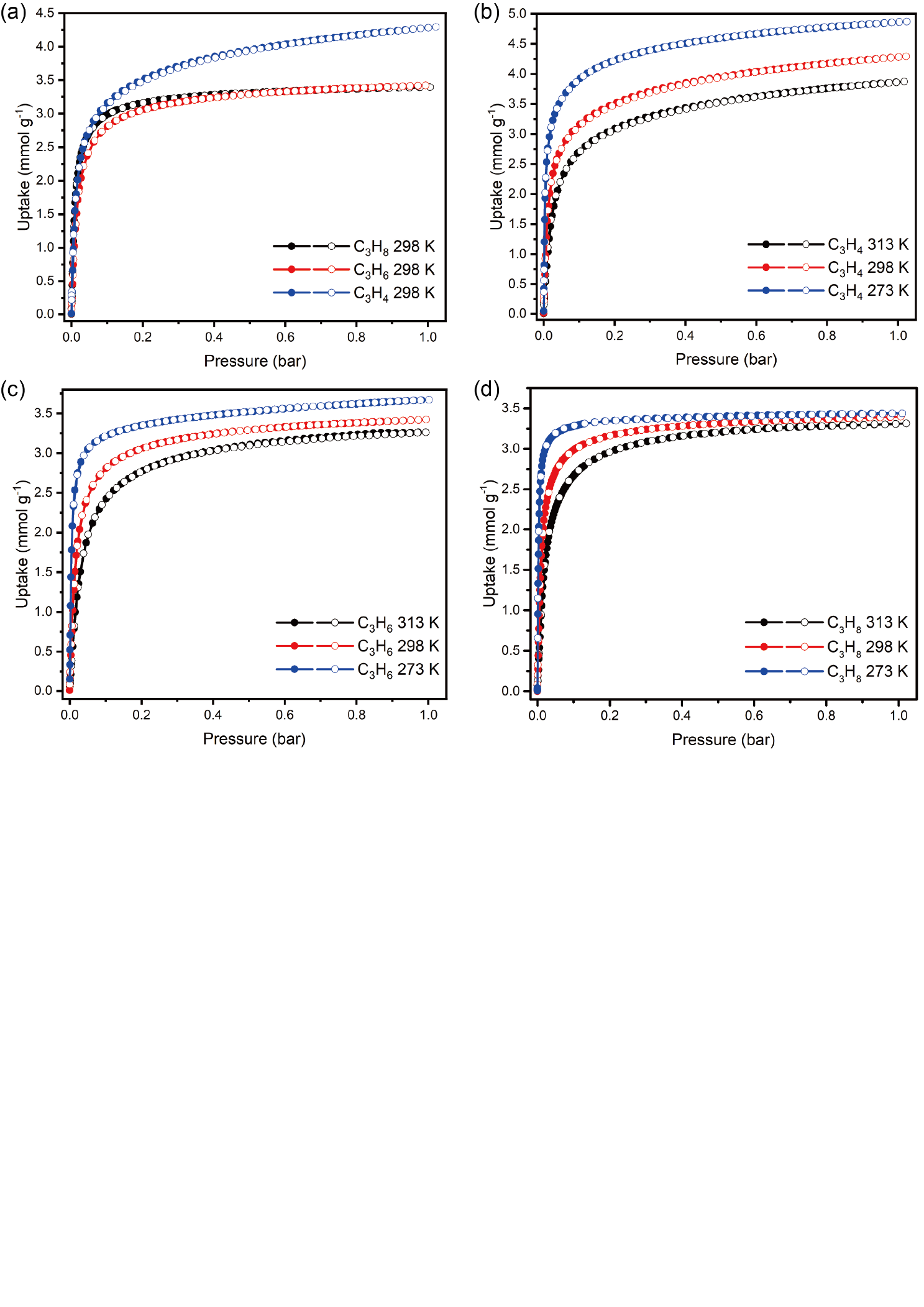


**Figure S17**. The comparison of C3H4, C3H6, and C3H8 sorption isotherms for **CMOM-7** at 298 K (a). Sorption isotherms for **CMOM-7** at 273 K, 298 K and 313 K, for adsorbates: C3H4 (b), C3H6 (c) and C3H8 (d) (closed and open symbols denote adsorption and desorption, respectively).

The sorption isotherms of CH4 and C2H6 were fitted with the Single-site Langmuir Freundlich (SSLF) equation12:


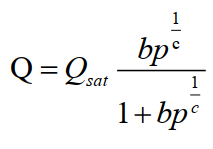


where *Q* is the gas uptake (mmol g-1), *p* is the pressure (kPa), *Qsat* is the saturation uptakes for the binding site (mmol g-1), *b* is the affinity coefficients of the binding site, and *c* is the index heterogeneity of the binding site.

The sorption isotherms of C3H8 were fitted with the Dual-site Langmuir Freundlich (DSLF) equation13:


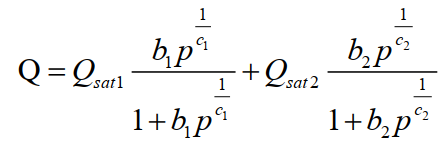


where *Q* is the gas uptake (mmol g-1), *p* is the pressure (kPa), *Qsat1* and *Qsat1* are the saturation uptakes for site 1 and site 2 (mmol g-1), *b1* and *b2* are the affinity coefficients of site 1 and site 2, and *c1* and *c2* are the index heterogeneity of site 1 and site 2.

**
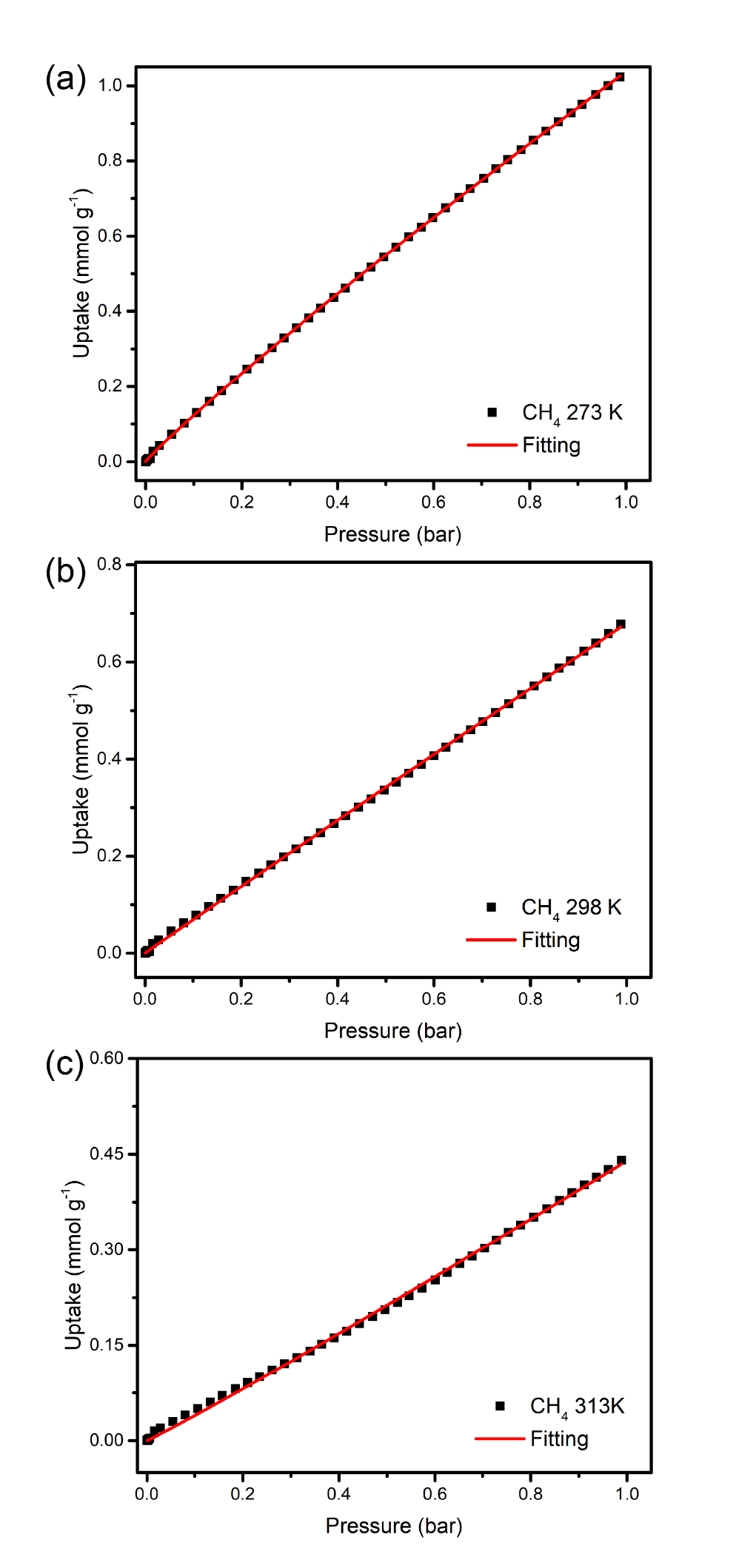
**

**Figure S18**. The SSLF fittings for CH4 adsorption isotherms at 273 K (a), 298 K (b) and 313 K (c).

**
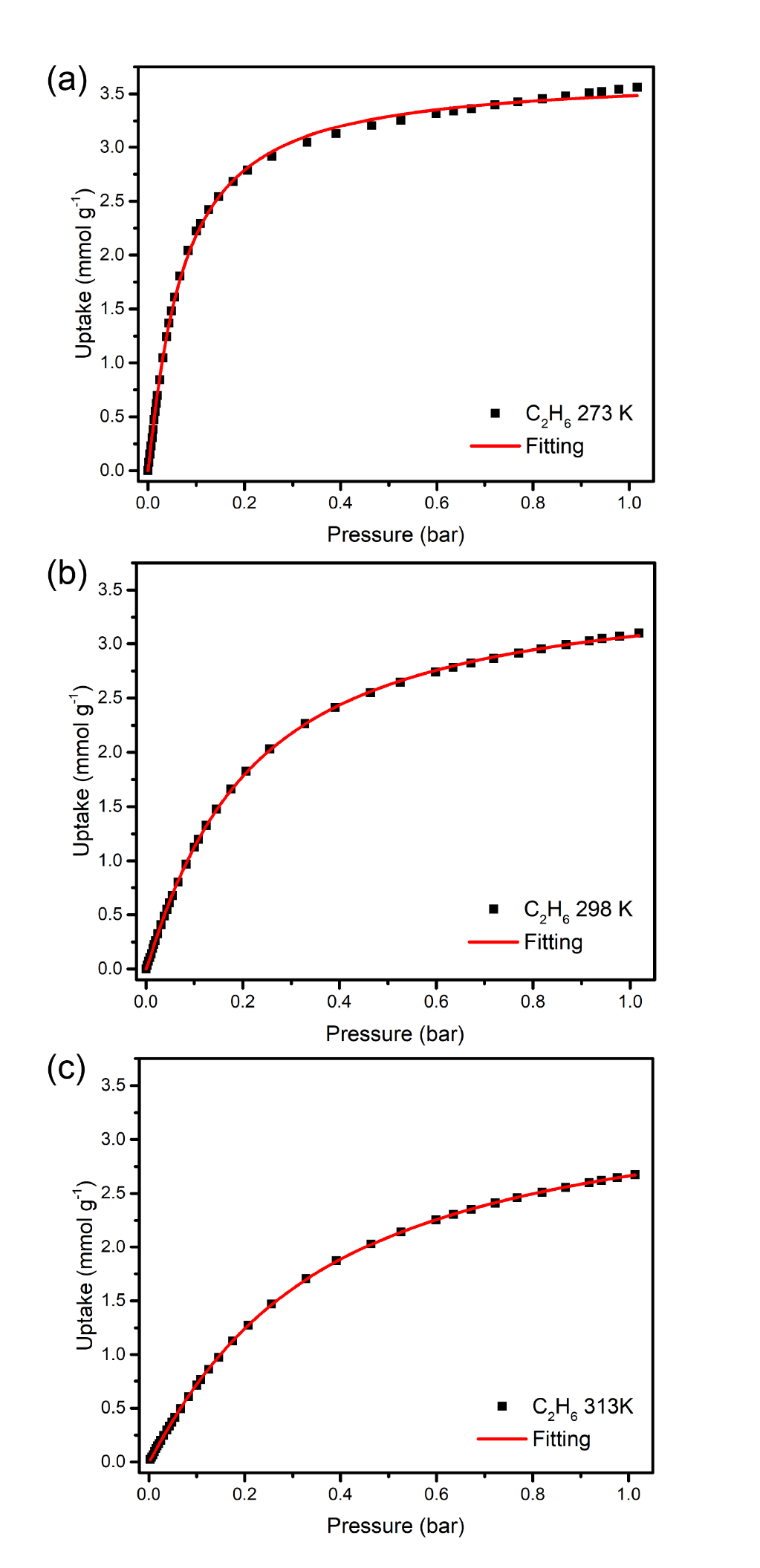
**

**Figure S19**. The SSLF fittings for C2H6 adsorption isotherms at 273 K (a), 298 K (b) and 313 K (c).

**
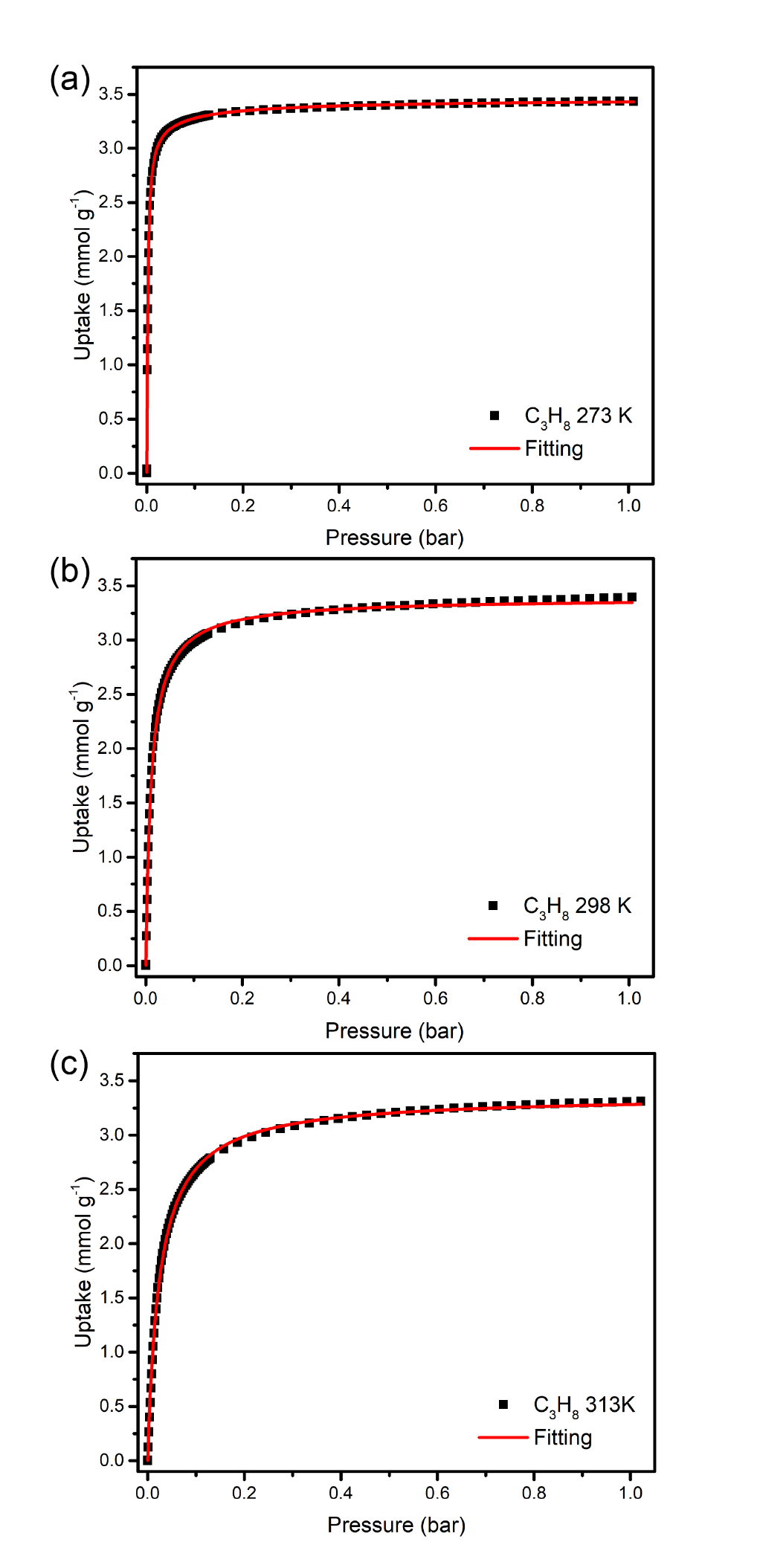
**

**Figure S20**. The DSLF fittings for C3H8 adsorption isotherms at 273 K (a), 298 K (b) and 313 K (c).

**Table S4.** The SSLF fitting parameters for pure CH4, C2H6 isotherms of **CMOM-7** at 273K, 298K and 313K.

| **CMOM-7** | *Qsat* (mmol g-1) | *b* (kPa-1) | *c* | R2 |
| --- | --- | --- | --- | --- |
| CH4 (273 K) | 37.18015 | 3.82039 × 10-4 | 1.06617 | 0.99997 |
| CH4 (298 K) | 58.83737 | 1.17025 × 10-4 | 1.00012 | 0.99979 |
| CH4 (313 K) | 69.79457 | 4.98947 × 10-5 | 0.95053 | 0.99907 |
| C2H6 (273 K) | 3.66678 | 0.11466 | 0.90260 | 0.99925 |
| C2H6 (298 K) | 3.60439 | 0.03603 | 0.90926 | 0.99992 |
| C2H6 (313 K) | 3.51048 | 0.02106 | 0.92012 | 0.99997 |

**Table S5.** The DSLF fitting parameters for C3H8 isotherms of **CMOM-7** at 273K, 298K and 313K.

| **CMOM-7** | *Qsat1* (mmol g-1) | *b1* (kPa-1) | *c1* | *Qsat2* (mmol g-1) | *b2* (kPa-1) | *c2* | R2 |
| --- | --- | --- | --- | --- | --- | --- | --- |
| C3H8 (273 K) | 0.94656 | 0.83346 | 1.42934 | 2.52869 | 8.81702 | 0.70554 | 0.99995 |
| C3H8 (298 K) | 1.95252 | 1.53110 | 0.69963 | 1.54008 | 0.39245 | 1.29478 | 0.99999 |
| C3H8 (313 K) | 2.38300 | 0.29559 | 1.15455 | 1.06789 | 0.60573 | 0.58415 | 0.99999 |

The experimental isosteric heat of adsorption (Qst) values for various gases in **CMOM-7** were determined by the DSLF fitting parameters of the adsorption isotherms at 298 K, 273 K and 313 K through the Clausius-Clapeyron equation:


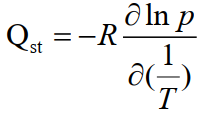


where T is the temperature (K), R is the ideal gas constant (8.314 J mol-1 K-1) and *p* is the pressure (kPa). The partial derivative term represents the slope of the plot of ln *P* versus for a number of isotherms at different temperatures at various loadings. Therefore, *Q*st could be simplified as following equation:


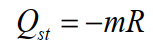


where m stands for the slope of ln *p* versus . So Qst could be calculated by the following equation for three different temperatures and their corresponding pressures:


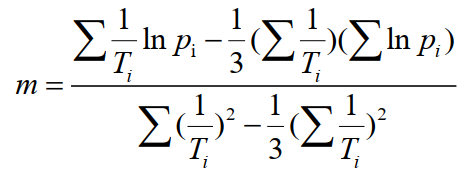


where pi values were back-calculated for a range of uptakes using the DSLF equation via an iterative technique (e.g. the Newton-Raphson method).14

Adsorption selectivity of C3H8/CH4, C2H6/CH4 or C3H8/C2H6 mixed gases were predicted from single component adsorption isotherms using Ideal Adsorbed Solution Theory (IAST).15 The adsorption selectivity (Sads) defined for separation of a binary mixture of species *i* and *j* by the following equation:


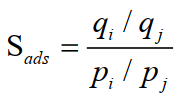


where *qi* and *qj* represent the molar loadings of component *i* and *j* that is in equilibrium with a bulk gas phase with partial pressures *pi* and *pj* in the mixture.

**
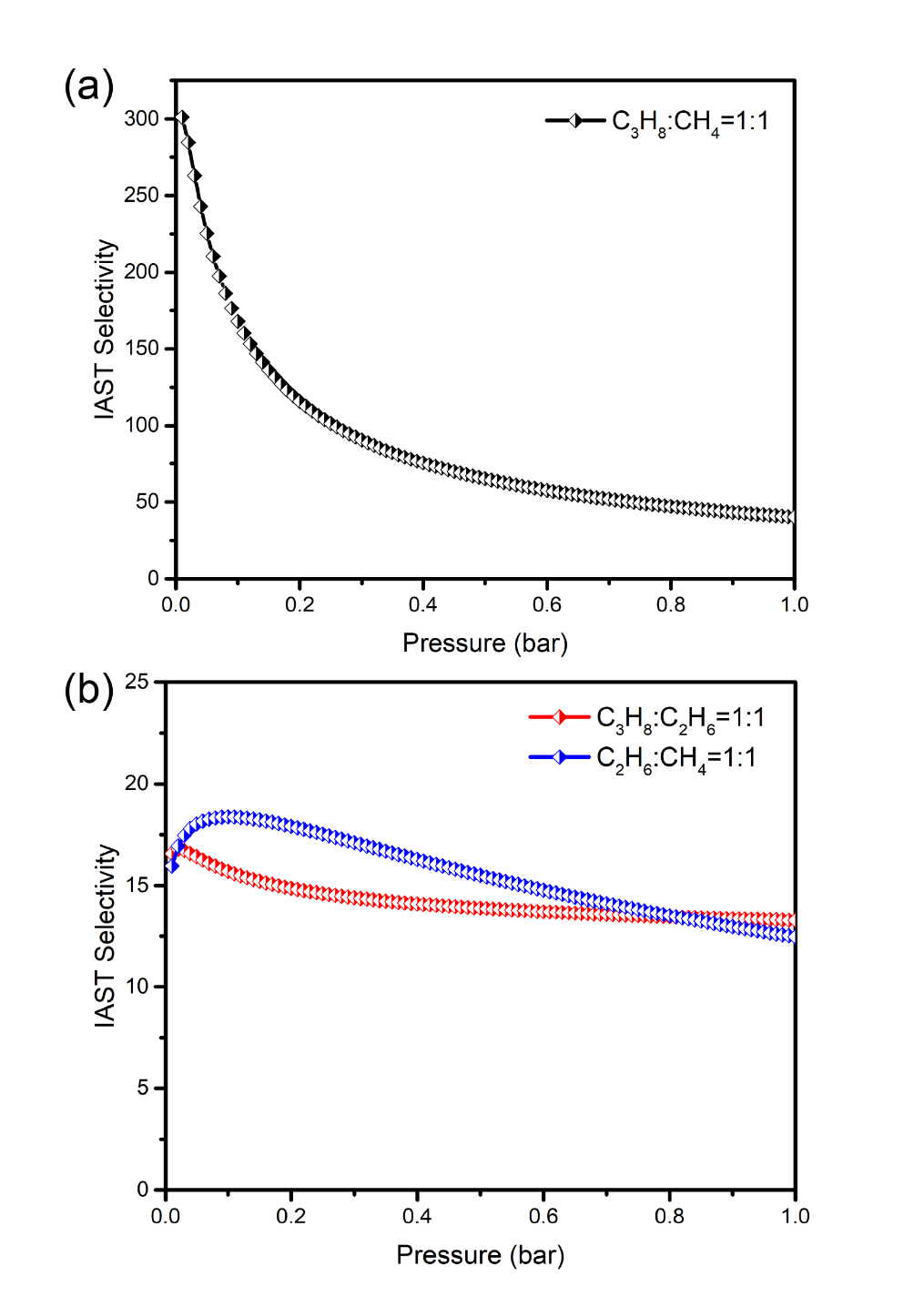
**

**Figure S21**. The IAST selectivity of equimolar binary mixture of C3H8/CH4 (a), C3H8/C2H6 (b) and C2H6/CH4 (b) at 298 K.

Dynamic column breakthrough (DCB) measurements

In the breakthrough experiments, ~0.88 g of activated **CMOM-7** was placed in a quartz tubing (Ф 6 mm x 400 mm, outer diameter = 8 mm) to form the fixed bed. First, the adsorbent bed was purged under a 20 cm3 min-1 flow of He gas at 80 °C for 1 h prior to breakthrough experiment. Upon cooling to room temperature, the gas flow was switched to the desired CH4/C2H6/C3H8 (85:10:5) or CH4/C2H6 (85:10) or CH4/C3H8 (85:5) or C2H6/C3H8 (10:5) gas mixture, maintained at the designed flow rates. The outlet composition was continuously monitored by a Shimadzu Nexis GC-2030 gas chromatography (GC) with a flame ionization detector (FID) and a thermal conductivity detector (TCD) until complete breakthrough was achieved.

Uptakes were determined from the adsorption branch of the experiments, assuming negligible pressure drop and following equation:


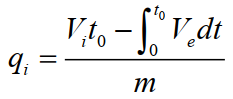


Here, *Vi* is the inlet flow rate of gas (ml min-1), *Ve* is the effluent flow rate of gas (mL min-1), *t0* is the adsorption saturation time (min) and *m* is the mass of the sorbent (g). Dead volume correction was applied by adjusting *t0* to account for time lag between inlet and outlet flows using black experiments for each inlet mixture composition.

The separation factor for the breakthrough experiment is determined using following equation:


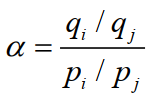


where *α* is the separation factor, *qi* and *qj* are the uptakes of gases *i* and *j*, and *pi* and *pj* are the partial pressure of gases *i* and j in the gas mixture.

Considering CH4 would elute in all the DCB experiments, the productivity of high-purify CH4 (PCH4) was calculated by the following equation:


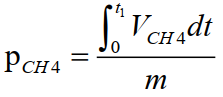


where *VCH4* is the effluent flow rate of CH4 (mL min-1), *m* is the mass of the sorbent (g), and *t1* is the breakthrough time of the second elute gas.


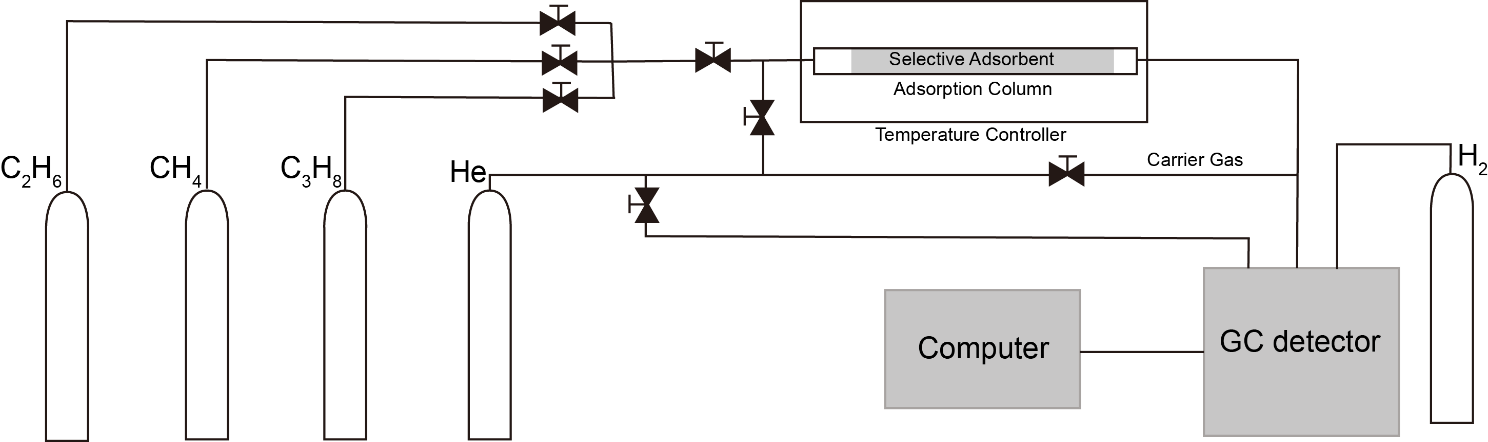


**Figure S22**. Schematic illustration of the apparatus for the breakthrough experiments.


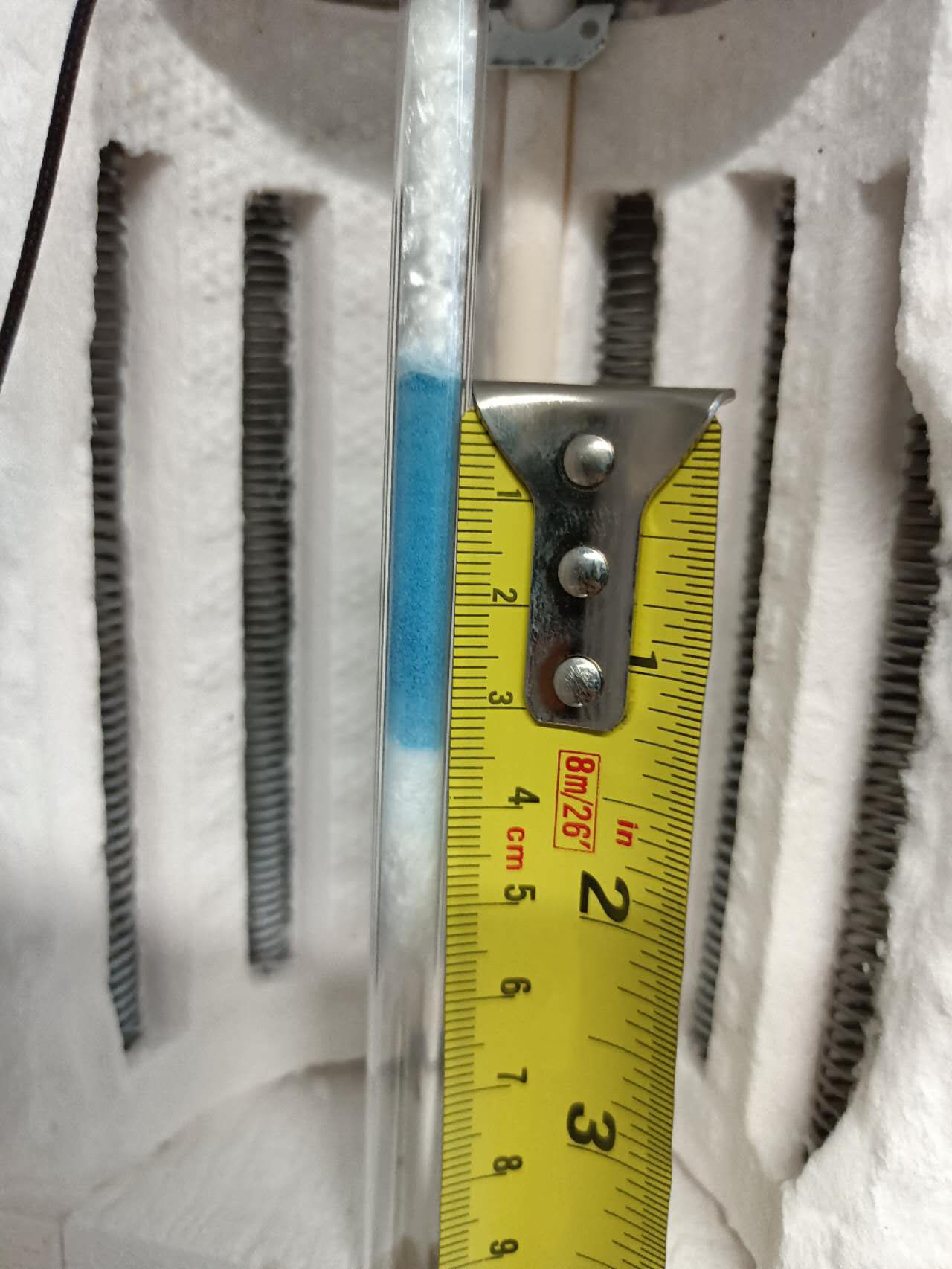


**Figure S23**. The quartz tubing with the sample of activated **CMOM-7** in the breakthrough equipment.


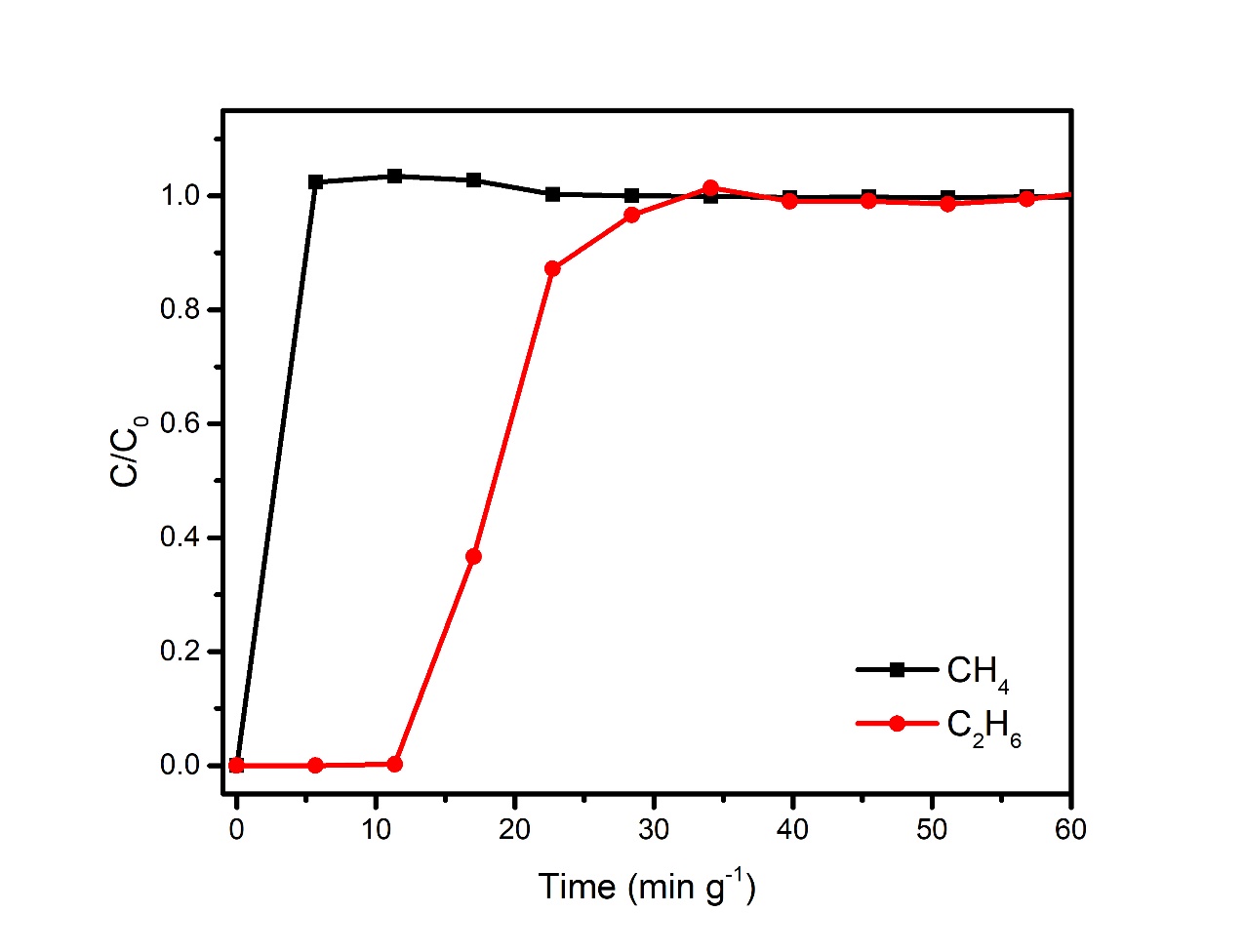


**Figure S24**. Experimental breakthrough curves of CH4/C2H6 (85/10, v/v) mixture for **CMOM-7** at 298 K, and 1 bar (flow rate: 9.5 mL min-1).


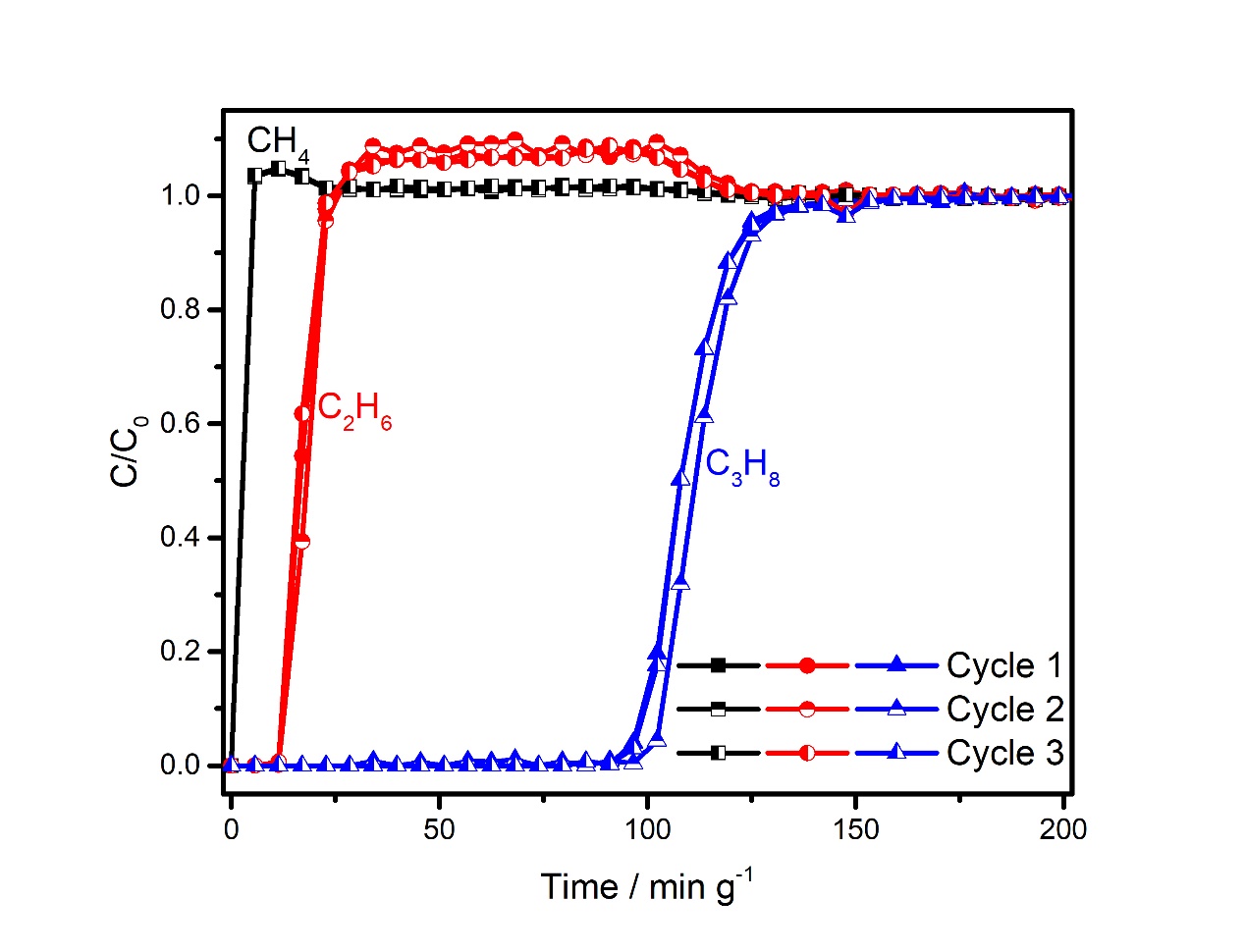


**Figure S25**. Three cycles of experimental breakthrough curves of CH4/C2H6/C3H8 (85/10/5, v/v/v) mixture for **CMOM-7** at 298 K, and 1 bar (flow rate: 10 mL min-1).


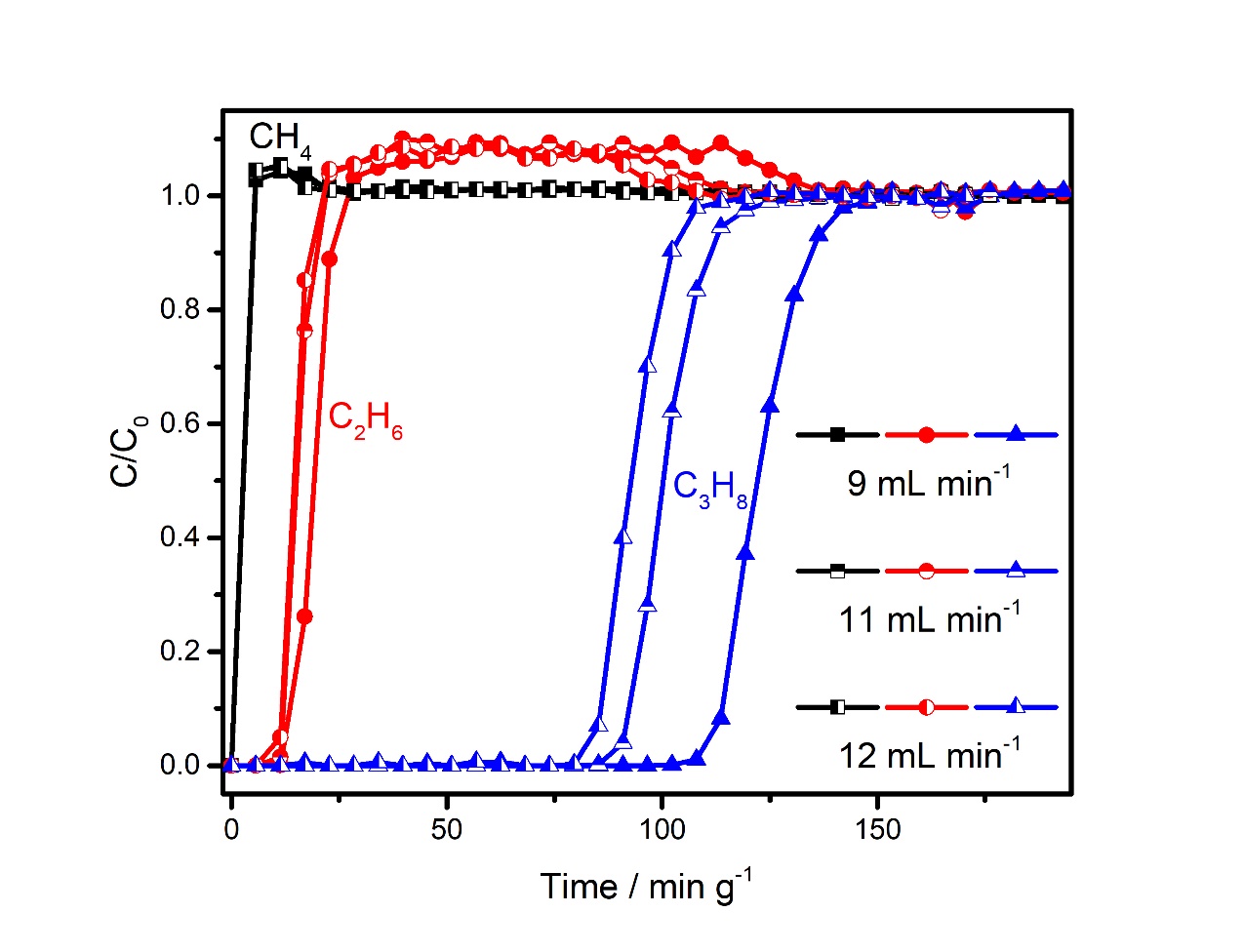


**Figure S26**. The experimental breakthrough curves of CH4/C2H6/C3H8 (85/10/5, v/v/v) mixture for **CMOM-7** at 298 K, and 1 bar with different flow rates (9 mL min-1, 11 mL min-1 and 12 mL min-1).

Under the different flow rates (9, 11, and 12 mL min-1), CH4 was found to elute out immediately, so, the corresponding dynamic capacities cannot be measured. Under 9 mL min-1 flow rate, C2H6 and C3H8 were detected to elute out at 11.4 min g-1 and 102.3 min g-1, respectively, which correspond to the dynamic capacities of C2H6 and C3H8 as 10.4 and 55.4 cm3 g-1, respectively. Under 11 and 12 mL min-1 flow rates, C2H6 was detected to elute out at 5.7 min g-1, and the corresponding dynamic capacities were 9.4 and 10.9 cm3 g-1. C3H8 was detected to elute out at 85.2 min g-1 and 79.5 min g-1 under 11 and 12 mL min-1 flow rates, respectively, and the corresponding dynamic uptakes were 55.7 and 56.0 cm3 g-1. Sum of the breakthrough times under 9, 11, and 12 mL min-1 were 113.7, 90.9 and 85.2 min g-1, and the corresponding total dynamic uptakes (for all the adsorbed sorbates) were 65.8 cm3 g-1, 65.1 cm3 g-1 and 66.9 cm3 g-1. More details of the results of DCB experiments were shown in Table S6.

**Table S6.** A summary of the details of DCB experiments.

| Ratio | Flow rate  (mL min-1) | Breakthrough Time  (min g-1) | | | Productivity of CH4  (cm3 g-1) | Dynamic Uptake  (cm3 g-1)* | | Selectivity* |
| --- | --- | --- | --- | --- | --- | --- | --- | --- |
|  |  | CH4 | C2H6 | C3H8 |  | C2H6 | C3H8 | C3H8/C2H6 |
| CH4/C2H6/C3H8 = 85:10:5 | 10 (cycle 1) | immediately | 11.4 | 90.9 | 75.2 | 10.8 | 54.8 | 10.1 |
|  | 10 (cycle 2) | immediately | 11.4 | 96.6 | 75.2 | 10.6 | 56.3 | 10.6 |
|  | 10 (cycle 3) | immediately | 11.4 | 90.9 | 75.4 | 10.9 | 54.9 | 10.1 |
|  | 9 | immediately | 11.4 | 102.3 | 67.3 | 10.4 | 55.4 | 10.7 |
|  | 11 | immediately | 5.7 | 85.2 | 27.7 | 9.4 | 55.7 | 11.9 |
|  | 12 | immediately | 5.7 | 79.5 | 30.3 | 10.9 | 56.0 | 10.3 |
| CH4/C3H8 = 85:5 | 9 | immediately | - | 108.0 | 904.2 | - | 62.2 | - |
| CH4/C2H6 = 85:10 | 9.5 | immediately | 11.4 | - | 74.4 | 18.8 | - | - |
| C2H6/C3H8 = 10:5 | 1.5 | - | 34.1 | 119.3 | - | 3.9 | 72.7 | 37.3 |

*For the reason that CH4 elute immediately in the DCB experiments, the dynamic uptakes of CH4 cannot be accurately calculated by integration from the DCB curves, and CH4 related selectivity cannot be accurately calculated.

CSD survey

CSD survey was conducted for finding C3H8 molecules crystallographically identified within MOM structures through the ConQuest software (version 2022.3.0). CSD database version 5.43 (November 2021) and the updates Mar 2022, Jun 2022, Sep 2022 and Nov 2022 were employed.16

**
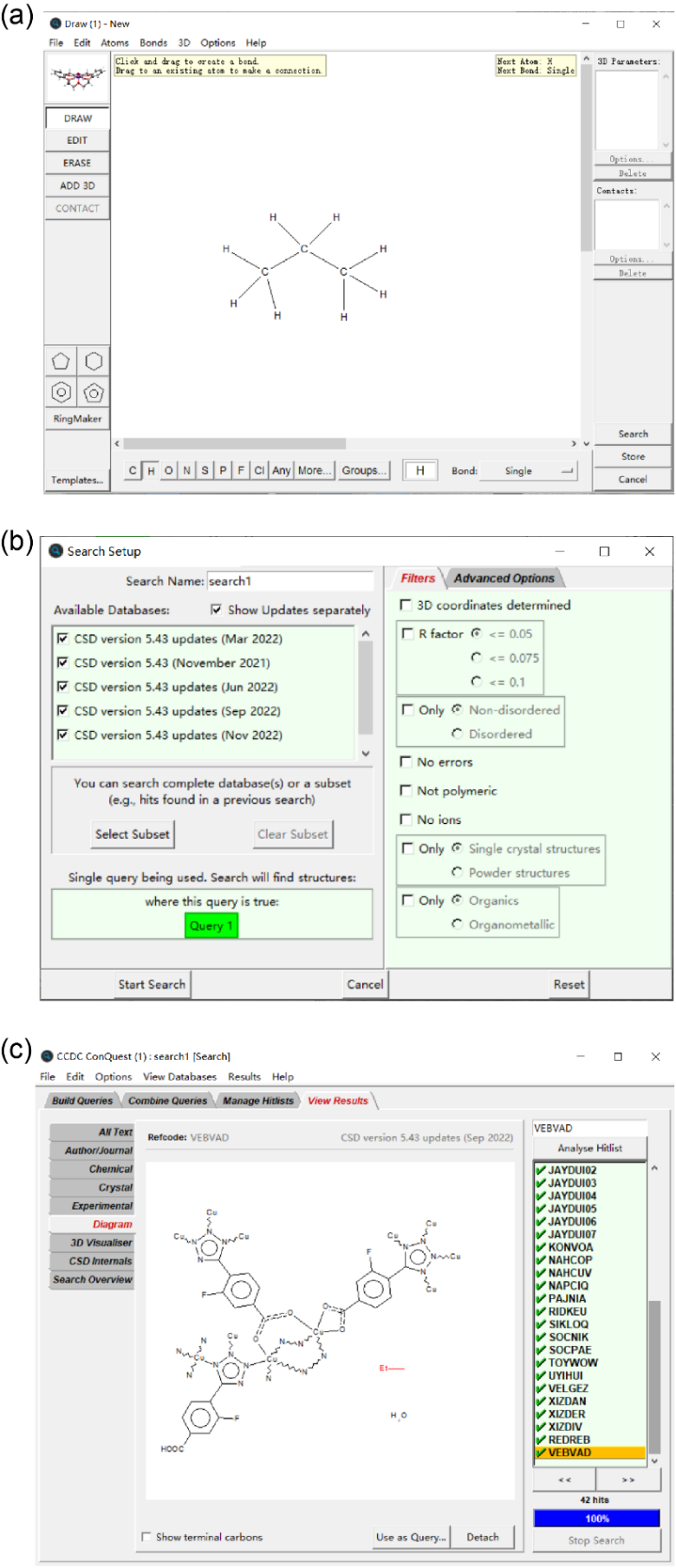
**

**Figure S27**. CSD database mining results on C3H8 loaded MOM crystals. (a) The input image. (b) Search setup details. (c) CSD survey results.

**Table S7**. A summary on C3H8 loaded MOMs in CSD research result.

| Refcode | MOM name and/or formula | Number of C3H8 Binding Site | Occupancy | Reference |
| --- | --- | --- | --- | --- |
| AXOWES | [Co2(ndc)2(bipy)]n[a] | 1 | 0.25 | 17 |
| IVELUS | [Mn(ina)2][b] | 1 | 0.5 | 18 |
| IWEKIH | MFM-520 | 1 | 0.125 | 19 |
| SOCNIK | MAF-23 | 1 | 0.5 | 20 |
| SOCPAE | MAF-23-O | disordered | - |
| TOYWOW | NKU-FlexMOF-1 | 1 | 0.5 | 21 |
| UYIHUI | JNU-3a | 1 | 1 | 22 |
| VELGEZ | [Cu2(phen)2(O3P(CH2)3PO3)(V2O5)][c] | 1 | 1 | 23 |
| XIZDAN | ZIF-8 | disordered | - | 24 |
| XIZDER |
| XIZDIV |
| REDREB | NbOFFIVE-1-Ni | 1 | 0.808 | 25 |
| VEBVAD | SNNU-Ba68 | 3 | 1; 1; 1 | 26 |

[a] ndc = 2,6-napthalene dicarboxylate; [b] ina = isonicotinate; [c] phen = 1,10-phenantroline.

Water vapor sorption studies

Dynamic water vapor sorption (DVS) studies for the methanol exchanged sample, **CMOM-7-MeOH**, were performed on a DVS Adventure Water Vapor Sorption Analyzer (Surface Measurement System) which gravimetrically measures the uptake and loss of vapor using air as a carrier gas. Pure water was used as the adsorbate for these measurements and temperature was maintained at 298 K by enclosing the system in a temperature-controlled incubator. Prior to measurement, the sample was in-situ activated at 80 °C, 0 relative humidity (RH). The mass of the sample was determined by comparison to an empty reference pan and recorded by a high-resolution microbalance with a precision of 0.1 µg. Sorption isotherm was measured from 0 to 95% RH stepwise with a convergence equilibrium criterion dm/dt = 0.01 %/min. The minimum and maximum equilibration times for each step were 10 and 360 min, respectively.


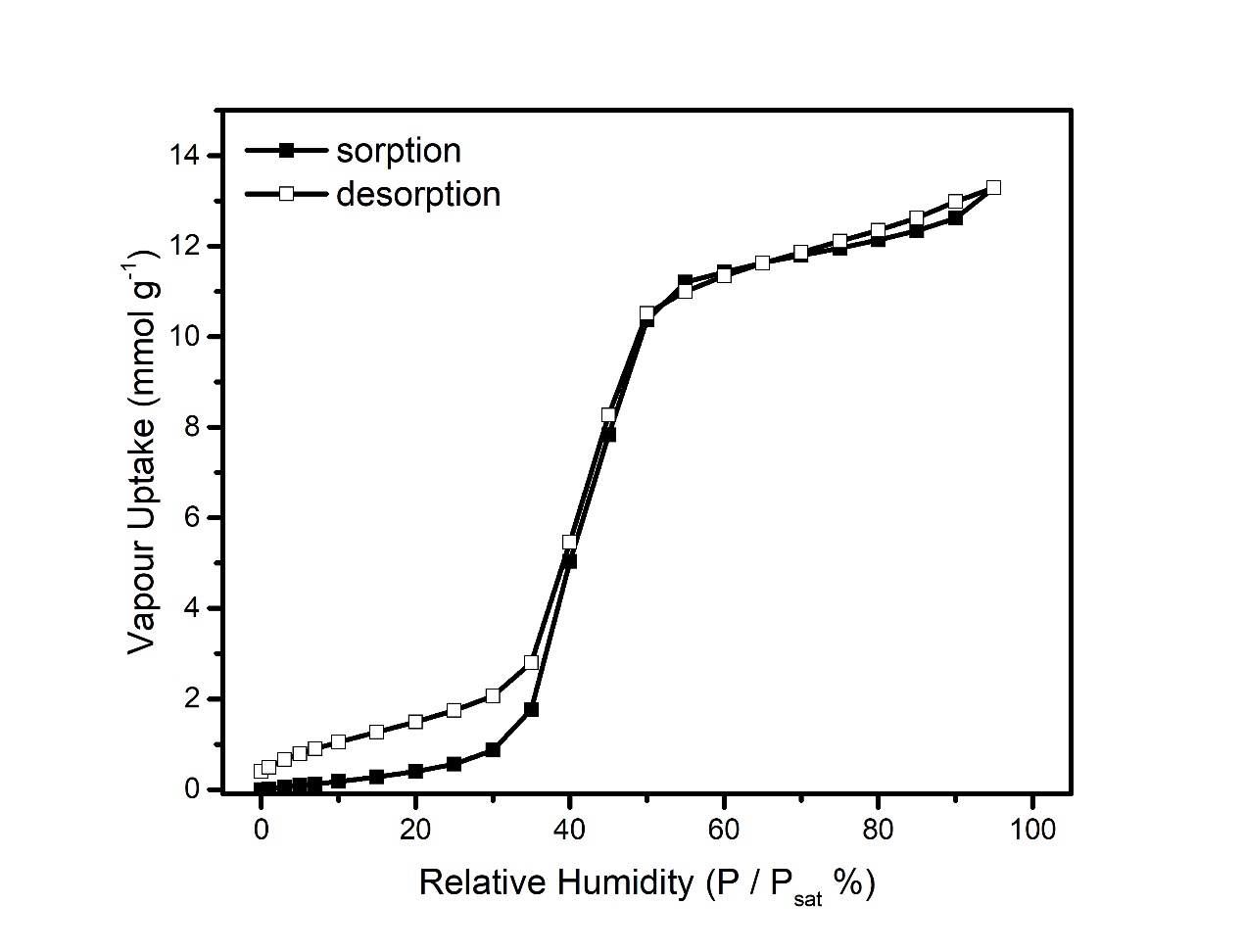


**Figure S28**. Water vapor sorption isotherm for **CMOM-7** at 298 K (closed and open symbols denote adsorption and desorption, respectively).

The kinetics and recyclability tests were performed at 25 °C on Surface Measurement Systems DVS Intrinsic using air as a carrier gas to gravimetrically measure the uptake and loss of vapor. The mass of the sample was determined by comparison to an empty reference pan and recorded by a high-resolution microbalance with a precision of 0.1 µg. Kinetics was measured between two points 0 and 60% RH with a convergence equilibrium criterion dm/dt =0.01 %/min.

The recyclability test was done by performing 100 cycles, each cycle consisting of 25 min adsorption step (60%RH) and 15 min desorption step (0%RH).


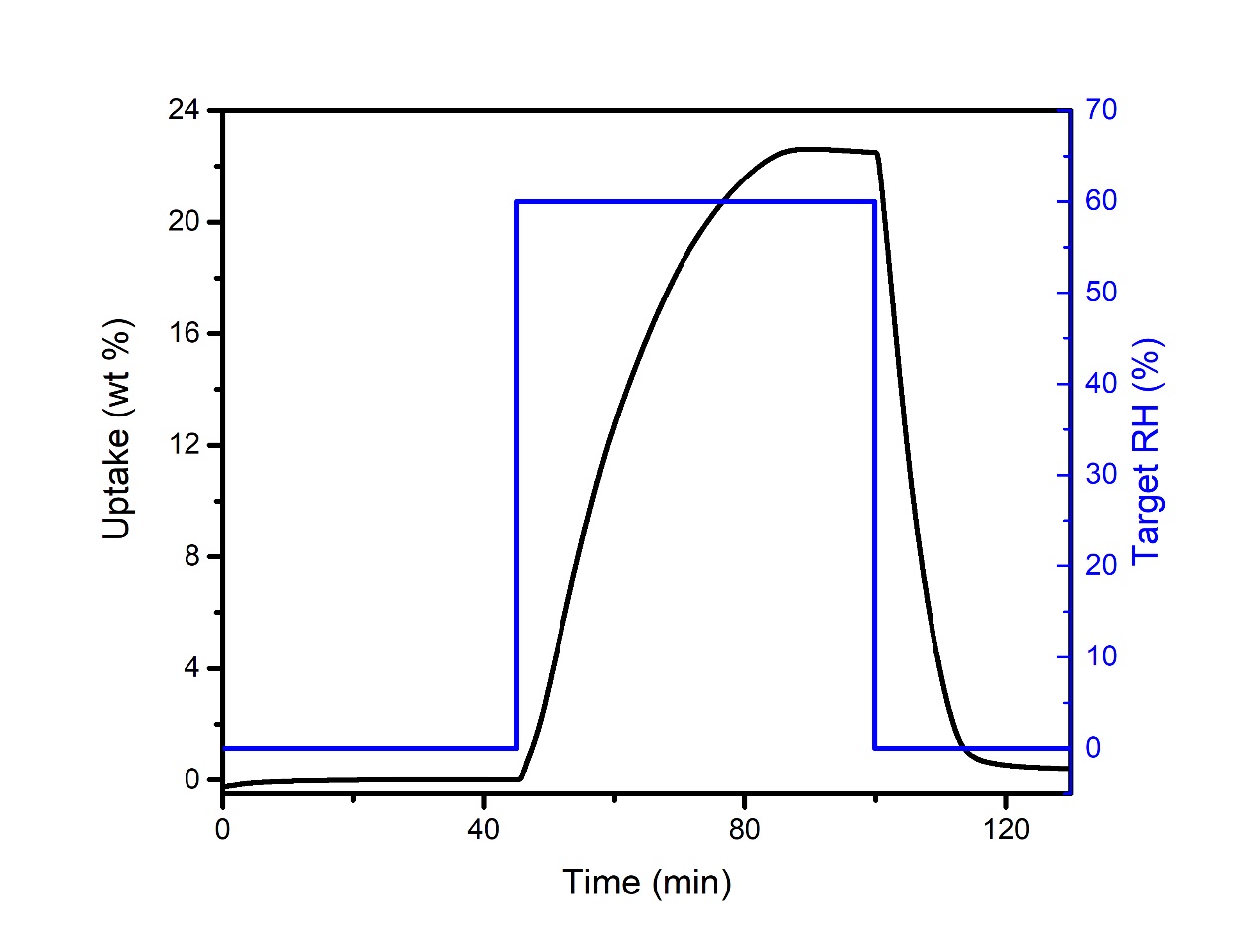


**Figure S29**. Water vapor adsorption kinetics plot for **CMOM-7** at 298 K.


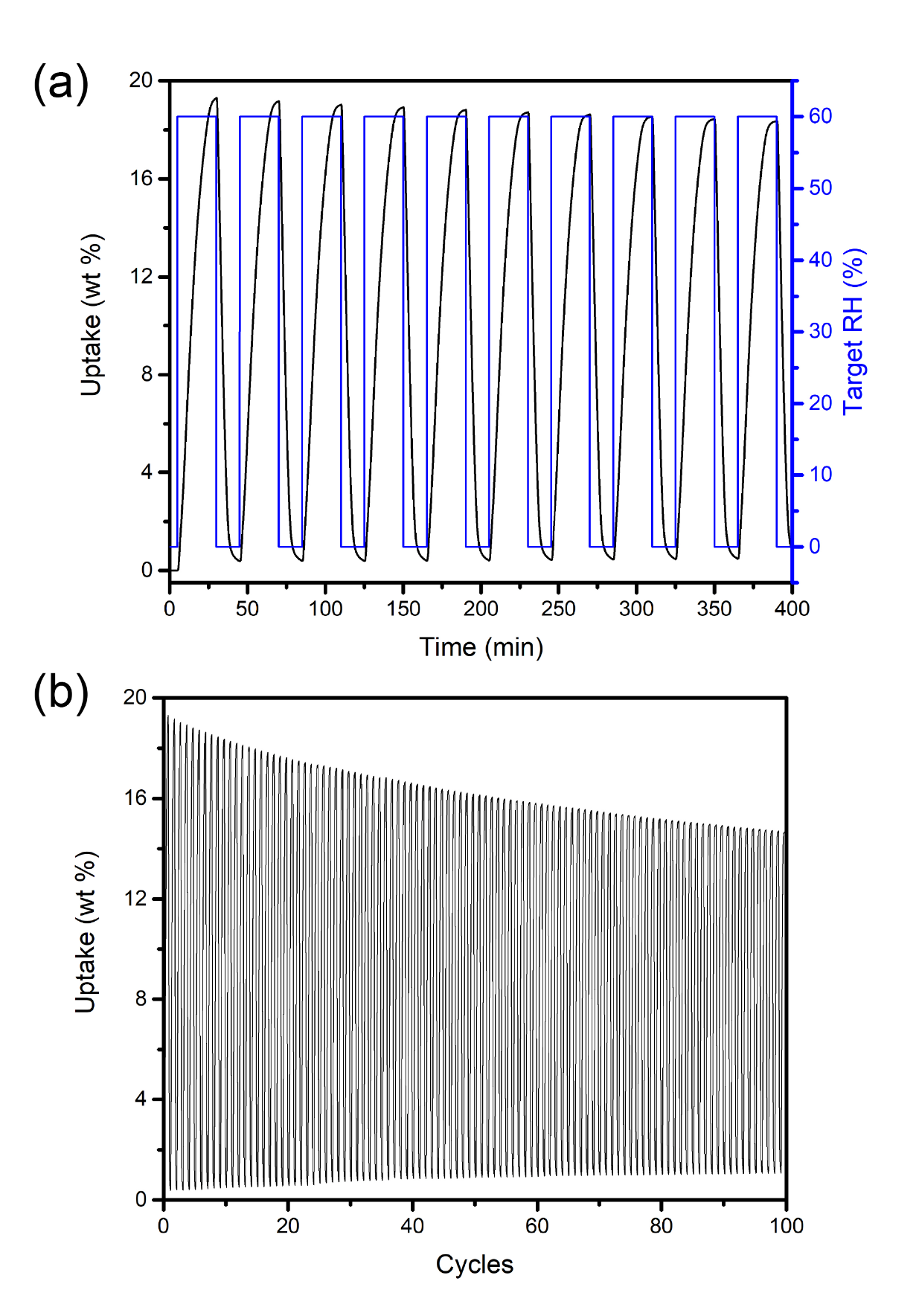


**Figure S30**. Water vapor adsorption-desorption cycles for **CMOM-7** at 298 K. (a) The plot for the first 10 consecutive cycles. (b) The plot covering the full 100 consecutive cycles.

Comparison of gas sorption and separation performance

**Table S8.** Comparison of CH4, C2H6, and C3H8 uptakes, *Qs*t at zero loading and IAST selectivities for binary gas mixtures of CH4, C2H4 and C3H8 with some reported materials at 298 K and 1 bar.

| **Materials** | Uptake (mmol g-1) | | | Qst (kJ mol-1) | | | IAST Selectivity[a] | | | Reference |
| --- | --- | --- | --- | --- | --- | --- | --- | --- | --- | --- |
|  | C3H8 | C2H6 | CH4 | C3H8 | C2H6 | CH4 | C3H8/CH4 | C3H8/C2H6 | C2H6/CH4 |  |
| **CMOM-7** | 3.40 | 3.10 | 0.68 | 31.2 | 27.9 | 20.9 | 151,  40[b] | 13.6,  13.3[b] | 17.1,  12.5[b] | **This work** |
| **MOF-303** | 4.74 | 4.96 | 0.86 | 34 | 24 | 19 | 5114 | NG | 26 | 27 |
| **MIL-160** | 5.08 | 4.65 | 0.94 | 35 | 28 | 19 | 174 | NG | 20 |  |
| **MIL-100(Fe)** | 6.78 | 2.22 | 0.36 | ~25 | ~22.5 | ~17.5 | 33.3 | NG | 6 | 28 |
| **MIL-101-Cr** | 3.35 | 1.59 | 0.49 | 29.2 | 22.2 | NG | 84.3 | NG | 22.5 | 29 |
| **MIL-101-Fe** | 3.29 | 1.25 | 0.45 | 34.5 | 25.6 | NG | 24.9 | NG | 15.4 |  |
| **MIL-101-Fe-NH2** | 3.32 | 1.35 | 0.46 | 28.3 | 24.6 | NG | 42.5 | NG | 11.6 |  |
| **Ni(TMBDC)(DABCO)0.5** | 5.54 | 5.81 | ~1.2 | 59 | 36 | 14 | 274 | NG | 29 | 30 |
| **ECUT-Th-10** | 2.89 | 1.72 | 0.42 | 33.6 | 27.3 | 26.4 | 54.5[b] | 9.31[b] | NG | 31 |
| **UiO-66** | 4 | 2.3 | 0.5 | NG | NG | NG | 32[b] | 2[b] | NG |  |
| **Ni(4-DPDS)2CrO4** | 1.09 | 1.49 | 0.95 | 65.2 | 41.6 | 28.4 | 131.6[b] | 0.17[b] | 33.9[b] | 32 |
| **C-PVDC-800** | 5.21 | 5.28 | ~1.55 | 78.1 | 35.6 | 19.5 | 3387[b] | NG | 75[b] | 33 |
| **HOF-ZJU-201a** | 2.61 | 3.16 | 1.73 | 51 | 43.3 | 17.1 | 119[b] | NG | 45[b] | 34 |
| **HOF-ZJU-202a** | 1.85 | 2.53 | 1.5 | 53.3 | 33.7 | 11.4 | 40[b] | NG | 36[b] |  |
| **SNNU-Bai68** | ~3.35 | ~3.05 | ~1.04 | 38.6 | 33.6 | 20.4 | 254.2[c];  240.2[d];  232.3[e] | 9.8[f];  9.2[g] | 22.4[h] | 26 |
| **0.3Gly@HKUST-1** | 7.8 | 6.47 | ~1.05 | ~32 | ~27.1 | ~21.4 | 173[i] | NG | 12.6[j] | 35 |
| **HKUST-1** | ~7.05 | ~5.5 | ~1.04 | ~29 | ~25 | ~21.4 | 143[i] | NG | 12.5[j] |  |
| **BSF-4** | ~1.52 | ~1.42 | ~0.42 | 43.7 | 34.8 | 22 | 138[k] | NG | ~19[h] | 36 |
| **BSF-9** | ~1.36 | ~1.29 | ~0.42 | 32 | 34.1 | 22.6 | ~70[k] | NG | 25[h] |  |
| **MOF-801** | 2.98 | 2.26 | 0.53 | 48 | 43 | 19 | 255[l] | NG | 28[j] | 37 |
| **ZUL-C1** | 2.72 | 2.95 | ~1.05 | 54 | 33 | 23 | 158 | NG | 28 | 38 |
| **ZUL-C2** | 2.52 | 2.82 | ~1.35 | 71 | 45 | 23 | 741 | NG | 82 |  |
| **Cu-MOF** | ~5.98 | ~3.12 | ~0.33 | 40.7 | 15.6 | 7.4 | 203.6 | NG | 9.3 | 39 |
| **MIL-142A** | 5.32 | 3.82 | 0.54 | 26.6 | 25.5 | 13.7 | 1300[b] | NG | 13.7[b] | 40 |
| **BSF-1** | 1.94 | 1.57 | 0.47 | 47 | 32.9 | 25.5 | 353[b] | NG | 23[b] | 41 |
| **BSF-2** | 1.77 | 1.22 | 0.24 | 39.7 | 32.8 | 23.5 | 681 | NG | 25 | 42 |
| **JUC-100** | 6.07 | 4.11 | 0.46 | NG | 26.1 | 27.1 | 80[b] | NG | 11[b] | 43 |
| **JUC-103** | 5.45 | 3.82 | 0.52 | NG | 22.6 | 23.5 | 65[b] | NG | 9[b] |  |
| **JUC-106** | 5.09 | 3.49 | 0.36 | NG | 24.2 | 26.1 | 80[b] | NG | 13[b] |  |
| **A-AC-3** | 11.34 | 7.09 | 1.38 | ~35 | ~26 | ~19 | 76.6[i] | NG | 16.9[j] | 44 |
| **A-AC-4** | 11.76 | 6.59 | 1.18 | ~34 | ~22.5 | ~18 | 88.8[i] | NG | 15.1[j] |  |
| **CTGU-15** | 12.13 | 2.13 | 0.40 | 21.7 | 21.3 | 19 | 170.7[b] | NG | 5.2[b] | 45 |
| **LIFM-W2** | 2.33 | 1.27 | 0.15 | 47.1 | 28.2 | 33.9 | 141 | NG | 14 | 46 |
| **W2-mty** | 1.91 | 1.1 | 0.1 | NG | NG | NG | NG | NG | NG |  |
| **UiO-67** | ~8.1 | ~3 | ~0.35 | 47.5 | 32.3 | ~22 | 73.7 | ~9.5 | 8.1 | 47 |
| **MFM-202a[m]** | 6.76 | 4.21 | 0.45 | 27 | 18 | 14 | 87[b] | ~7[b] | ~10[b] | 48 |
| **USTA-35a[n]** | 2.97 | 2.43 | 0.43 | ~41.5 | ~30 | ~17 | ~80[b] | NG | ~14[b] | 49 |
| **PCN-224** | 8.25 | 2.93 | 0.48 | 34 | 26 | 15 | ~609[b] | NG | ~12[b] | 50 |
| **Fe2(dobdc)[o]** | 6.1 | 5.5 | 0.8 | 33 | 25 | 20 | NG | NG | ~10.2[b] | 51 |
| **FIJ-C4** | 3.19 | 2.96 | 0.82 | ~38 | ~32 | ~22 | 293.4[b] | NG | 39.7[b] | 52 |
| **FJI-C1** | 6.33 | 3.9 | ~0.4 | 26.8 | 21.7 | 11.4 | 417[b] | NG | 22[b] | 53 |
| **JLU-Liu7** | 5.04 | 4.78 | 0.76 | 28.5 | 34.8 | 20.9 | 128.5[b] | NG | 50.4[b] | 54 |
| **JLU-Liu22** | 4.15 | 3.3 | 0.71 | 30.3 | 30.5 | 11 | 271.5[b] | NG | 14.4[b] | 55 |
| **JLU-Liu37** | 7.95 | 4.42 | 0.45 | 29.2 | ~19 | 17.5 | 206[b] | NG | 11[b] | 56 |
| **JLU-Liu38** | 8.39 | 4.96 | 0.36 | ~18 | ~19 | ~29 | 98[b] | NG | 15[b] |  |
| **Zr-OBBA** | ~0.80 | ~0.49 | ~0.16 | NG | NG | 19.1 | 105.6[b] | NG | ~13.1[b] | 57 |
| **Zr-SDBA** | ~2.32 | ~1.96 | ~0.58 | NG | NG | 19.3 | ~101[b] | NG | ~15[b] |  |
| **JLU-Liu45** | ~3.66 | ~3.79 | ~0.69 | NG | NG | 21.1 | ~50[b] | NG | ~30[b] |  |
| **Zn-BPZ-SA** | 2.73 | 2.97 | 0.64 | 32.7 | 26.4 | ~18.8 | 65.7,  40.6[b] | NG | 10.9,  10.5[b] | 58 |
| **NIIC-20-Et** | ~5.60 | ~2.38 | ~0.43 | 41.9 | 29.8 | 25.6 | 1110[p], 1234[q] | 29.0[p] | 20.7[b], 51.0[r] | 59 |
| **NIIC-20-Pr** | ~5.55 | ~2.41 | ~0.57 | 42.2 | 34.2 | 32.4 | 931[p], 874[q] | 28.0[p] | 22.2[b], 57.0[r] |  |
| **NIIC-20-Bu** | ~4.24 | ~2.46 | ~0.59 | 37.5 | 24.1 | 30.2 | 345[p], 261[q] | 10.4[p] | 16.7[b], 37.0[r] |  |
| **NIIC-20-Pe** | ~3.47 | ~2.24 | ~0.54 | 38.0 | 25.4 | 30.3 | 387[p], 362[q] | 10.4[p] | 14.6[b], 30.0[r] |  |
| **NIIC-20-Gl** | ~4.46 | ~2.07 | ~0.47 | 40.6 | 31.5 | 30.9 | 861[p], 1024[q] | 25.2[p] | 24.2[b], 65.0[r] |  |
| **Ni2(L)2(HCOO)2⋅4H2O[s]** | ~3.56 | ~4.56 | ~0.68 | ~80 | 20.24 | 20.71 | 638.9[b] | 3.5[b] | 61.0[b] | 60 |
| **SNNU-Bai76** | ~2.63 | ~2.19 | ~0.76 | 44.4 | 29.9 | ~19 | 315.8[e] | NG | 31.2[h] | 61 |
| **SNNU-Bai77** | ~2.14 | ~2.19 | ~0.71 | 42.5 | 29.7 | ~20 | 569.6[e] | NG | 42.1[h] |  |
| **SNNU-Bai78** | ~2.68 | ~2.72 | ~0.98 | 52.5 | 34.7 | ~18 | 301.9[e] | NG | 40.0[h] |  |
| **{(Me2NH2)[Ni3(µ3-OH)(CF3-BPDC-CF3)3(tpt)]}n** | ~5.09 | ~2.78 | ~0.46 | 29.2 | 20.2 | 7.74 | 60.1 | NG | 7.1 | 62 |
| **CFA-1-NiCl2-2.3** | 8.60 | 5.00 | 0.72 | 32.3 | 28.0 | 16.7 | 382.7 | NG | 15.2 | 63 |
| **CFA-1** | 6.52 | 3.90 | 0.72 | 33.1 | 29.3 | 18.3 | ~225.1 | NG | ~11.7 |  |
| **Fe-pyz** | ~3.06 | ~3.06 | ~1.00 | ~32 | ~52 | ~16 | 89 | NG | 23 | 64 |
| **Co-pyz** | ~3.19 | ~3.08 | ~1.12 | ~50 | ~41 | ~21 | 78 | NG | 22 |  |
| **Ni-pyz** | ~3.02 | ~2.68 | ~0.83 | ~69 | ~37 | ~22 | 53 | NG | 18 |  |

[a] The ratios of C3H8/CH4, C3H8/C2H6 and C2H6/CH4 are 5:85, 5:10 and 10:85, respectively, at 1 bar. [b] The equimolar binary mixture. [c] C3H8/CH4 = 1:99. [d] C3H8/CH4 = 3:97. [e] C3H8/CH4 = 5:95. [f] C3H8/C2H6 = 1:9. [g] C3H8/C2H6 = 3:7. [h] C3H8/C2H6 = 1:9. [i] C3H8/CH4 = 2:8. [j] C2H6/CH4 = 2:8. [k] C3H8/CH4 = 5:95. [l] C3H8/CH4 = 1:9. [m] 293 K data. [n] 296 K data. [o] 318 K data, H4dobdc = 2,5-dihydroxyterephthalic acid. [p] The equimolar binary mixture, zero coverage. [q] C3H8/C2H6 = 1:8, zero coverage. [r] C2H6/CH4 = 1:8, zero coverage. [s] L = 3-hydroxy-4-(4H-1,2,4-triazol-4-yl)benzoate.

**Table S9.** A summary of the C3H8/C2H6/CH4 ternary DCB reports.

| **Materials** | Flow rate  (mL min-1) | Sample Mass (g) | Geometric details of the sample (Ф a mm × b mm) | Breakthrough Time  (min g-1) [a] | | | Reference |
| --- | --- | --- | --- | --- | --- | --- | --- |
|  |  |  |  | CH4 | C2H6 | C3H8 |  |
| **CMOM-7** | 9 | 0.775 | 6 × 400 | Immediately | 11.4 | 102.3 | **This work** |
|  | 10 |  |  | Immediately | 11.4 | 90.9 |  |
|  | 11 |  |  | Immediately | 5.7 | 85.2 |  |
|  | 12 |  |  | immediately | 5.7 | 79.5 |  |
| **MOF-303** | 2 | 0.2 | NG | 15 | 120 | 760 | 27 |
| **MIL-160** | 2 | 0.2 | NG | ~30 | ~130 | 388 |  |
| **MIL-100(Fe)** | 5 | 0.2 | 5 × 150 | ~10 | ~30 | ~105 | 28 |
| **MIL-101-Cr** | 2 | 0.5 | 6 × 150 | ~3 | ~40 | ~125 | 29 |
| **MIL-101-Fe** | 3 | 0.5 | 6 × 150 | ~4 | ~20 | ~69 |  |
| **MIL-101-Fe-NH2** | 3 | 0.5 | 6 × 150 | ~4 | ~20 | ~60 |  |
| **Ni(TMBDC)(DABCO)0.5** | 5 | 0.3 | 3 × 275 | 13 | 80 | 267 | 30 |
| **ECUT-Th-10[b]** | 2 | 0.5 | 4.6 × 150 | 10 | 20 | 80 | 31 |
| **Ni(4-DPDS)2CrO4** | 1.5 | 0.1 | 4.6 × 100 | 260 | 1200 | 1000 | 32 |
| **C-PVDC-800** | 2 | 1.0 | 4.6 × 100 | 17 | 205 | 670 | 33 |
| **HOF-ZJU-201a** | 1.34 | 0.3434 | 4.6 × 50 | 55 | 277 | ~670 | 34 |
| **HOF-ZJU-202a** | 2.11 | 0.4008 | 4.6 × 50 | 52 | 175 | ~362 |  |
| **SNNU-Bai68** | 10 | 0.585 | 4 × 150 | ~19[c] | ~43[c] | ~188[c] | 26 |
|  |  |  |  | ~19[d] | ~43[d] | ~108[d] |  |
|  |  |  |  | ~19 | ~43 | ~92 |  |
|  |  |  |  | ~19[e] | ~43[e] | ~169[e] |  |
|  |  |  |  | ~19[f] | ~43[f] | ~111[f] |  |
|  |  |  |  | ~19[g] | ~43[g] | ~87[g] |  |
| **0.3Gly@HKUST-1** | 10 | 0.3 | 5 × 75 | ~3 | ~17 | ~83 | 35 |
| **HKUST-1** | 10 | 0.3 | 5 × 75 | immediately | ~13 | ~80 |  |
| **MOF-801** | 10 | 1.4 | 10 × 90 | 0.21 | 3.92 | 19.64 | 37 |
| **ZUL-C1** | 1 | 0.2466 | 4.6 × 30 | immediately | ~120 | 535 | 38 |
| **ZUL-C2** | 1 | 0.2875 | 4.6 × 30 | immediately | ~310 | 742 |  |
| **Cu-MOF** | 8 | 0.1308 | 6 × 100 | immediately | ~1.3 | ~3.3 | 39 |
| **MIL-142A** | 10 | 0.2 | NG | immediately | ~5 | ~60 | 40 |
| **BSF-1** | 1 | 0.505 | 4.6 × 100 | ~19.8 | ~99 | ~277 | 41 |
|  | 10 | 0.505 | 4.6 × 100 | immediately | ~9.9 | ~40 |  |
|  | 13.5 | 2.13 | 10 × 100 | immediately | ~6.6 | ~23 |  |
| **BSF-2** | 4 | 0.2808 | 4.6 × 50 | immediately | 25 | 77 | 42 |
| **A-AC-3**[i] | 10 | 0.2 | 5 × 70 | ~5 | ~70 | ~165 | 44 |
| **A-AC-4**[i] | 10 | 0.2 | 5 × 70 | ~5 | ~60 | ~170 |  |
| **CTGU-15** | 2 | 0.3 | 6 × 270 | 7.7 | 20 | 59.3 | 45 |
| **LIFM-W2** | 3 | 1 | 6 × 70 | 3 | 12 | 72 | 46 |
| **W2-mty** | 3 | 1 | 6 × 70 | 6 | 30 | 108 |  |
| **Zn-BPZ-SA** | NG | 0.8 | 4.2 × 80 | immediately | 16.2 | 118 | 58 |
| **NIIC-20-Pr[h]** | 20 | 0.3 | 2 × 160 | ~25 | ~120 | ~250 | 59 |
| **Ni2(L)2(HCOO)2⋅4H2O** | 5 | 0.82 | 6 × 70 | 2.6 | 35 | 190 | 60 |
| **SNNU-Bai76** | 10 | 0.6196 | 4 × 150 | immediately | 20.7 | 53.1 | 61 |
| **SNNU-Bai77** | 10 | 0.6103 | 4 × 150 | immediately | 14.4 | 47.2 |  |
| **SNNU-Bai78** | 10 | 0.9147 | 4 × 150 | immediately | 23.2 | 63.5 |  |
| **{(Me2NH2)[Ni3(µ3-OH)(CF3-BPDC-CF3)3(tpt)]}n** | 4 |  |  | 2.97 | 23.2 | 58.1 | 62 |
| **CFA-1-NiCl2-2.3** | 2 | 0.250 | 50 × 150 | immediately | ~36 | ~272 | 63 |
| **Fe-pyz** | 3 | 0.379 | 4 × 300 | 22 | 97 | 216 | 64 |
| **Co-pyz** | 3 | 0.518 | 4 × 300 | 16 | 103 | 197 |  |
| **Ni-pyz** | 3 | 0.369 | 4 × 300 | 11 | 89 | 144 |  |

[a] CH4/C2H6/C3H8 = 85:10:5. [b] CH4/C2H6/C3H8 = 1:1:1. [c] CH4/C2H6/C3H8 = 90:9:1. [d] CH4/C2H6/C3H8 = 88:9:3. [e] CH4/C2H6/C3H8 = 90:9:1 with 74% RH. [f] CH4/C2H6/C3H8 = 88:9:3 with 74% RH. [g] CH4/C2H6/C3H8 = 85:10:5 with 74% RH. [h] CH4/C2H6/C3H8 = 1:1:8. [i] activated carbons.

Modelling studies

All theoretical calculations were performed using Accelrys Materials Studio 7.0 software.65 Simulations in **CMOM-7** were performed on the single X-ray crystallographic structure.The crystal structures of **CMOM-7** were chosen for related simulations without further geometry optimization. Grand Canonical Monte Carlo (GCMC) simulations of **CMOM-7** were performed using the adsorption and locate module. During the simulation, the framework is considered rigid. Partial charges for atoms of **CMOM-7** were derived from QEq method and QEq_neutral1.0 parameter.66 The simulations were carried out at 298 K, adopting the locate task, Metropolis method in Sorption module and the universal force field (UFF).67 The partial charges on the atoms of gas molecules were also derived from QEq method. The interaction energy between hydrocarbon molecules and framework were computed through the Coulomb and Lennard-Jones 6-12 (LJ) potentials.68 The cutoff radius was chosen as 12.5 Å for the LJ potential and the long-range electrostatic interactions were handled using the atom-based summation method. The loading steps and the equilibration steps were 1 × 106, the production steps were 5 × 107. The locate task simulated the beneficial adsorption sites with a single guest molecule. The static binding energy was calculated using first-principles density funtional theory (DFT) in the CASTEP code.69 The generalized gradient approximation (GGA) with the Perdew-Burke-Ernzerhof (PBE) functional and on-the-fly generated ultrasoft pseudopotentials were used.70-71 Grimme (G06) semiempirical methods to describe the long-range van der Waals interactions.72 A cutoff energy of 450 eV and a 1 × 1 × 1 k-point mesh was found to be enough for the total energy to converge within 1 × 10−5 eV·atom−1. The binding energy between the framework and gas molecule was calculated using:


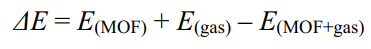


in which *E(MOF+gas)* is the total energy of MOF and the adsorbed gas molecule, and *E(MOF)* and *E(gas)* are the individual energies of the framework and gas molecule, respectively.


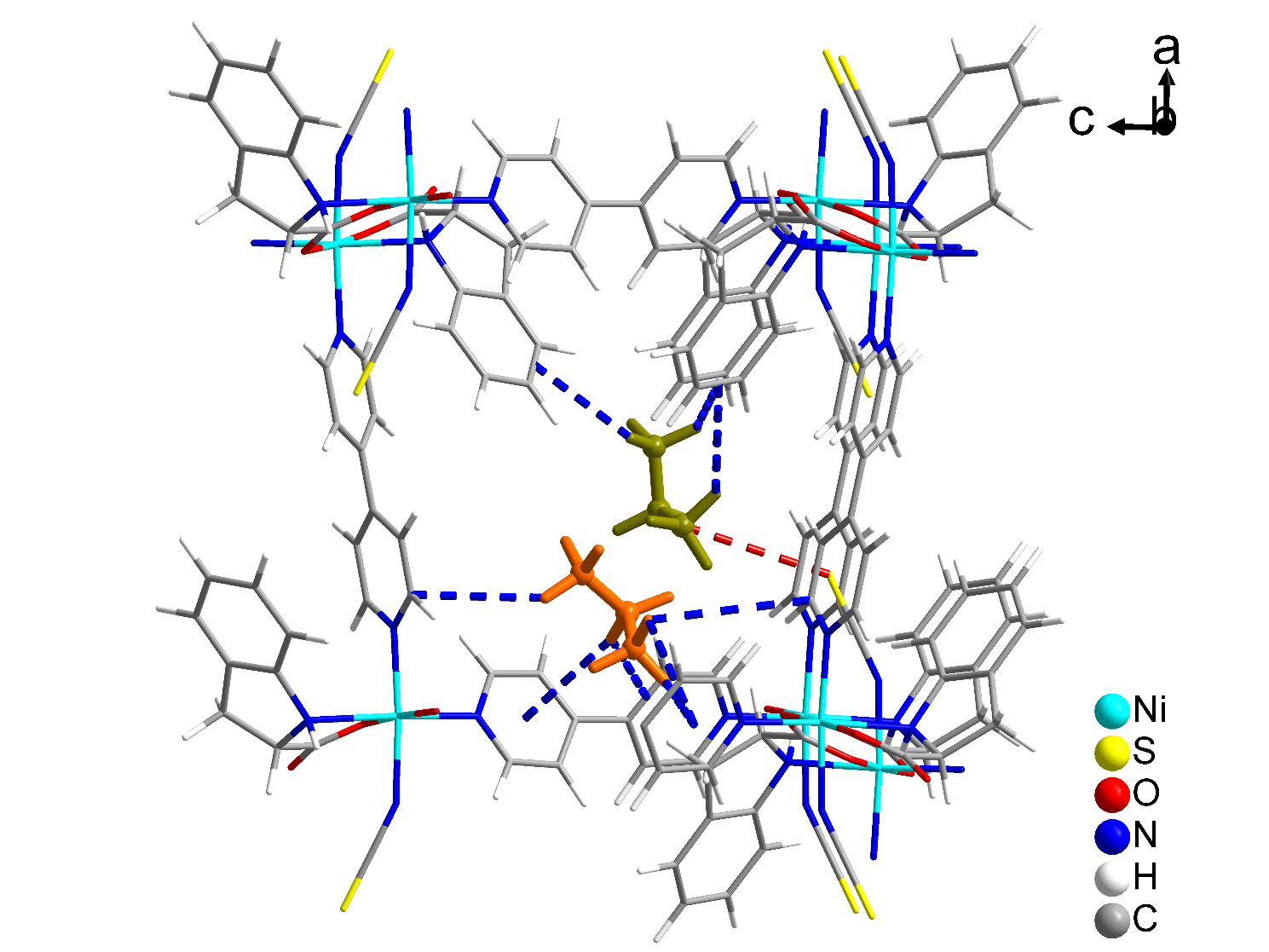


**Figure S31**. Binding sites of C3H8 molecules along the ultramicroporous channels of **CMOM-7**, determined by GCMC calculations. Atoms of C3H8 molecule in site-*I* was labelled in orange, and the atoms of C3H8 molecule in site-*II* was labelled in dark yellow. C-H···π interactions are labelled using blue dashed lines, and the H-bond was labelled by a red dashed line.

References

1. Singh, P. P.; Singh, V. P.; Khan, M. U.; Singh, D. Reaction of Amylmercury Thiocyanate with Thio and Selenocyanates of Co(II), Ni(II) and Zn(II). *Synth. React. Inorg. M.* **1984**, *14* (1), 111-128. DOI: 10.1080/00945718408057516.
2. Krause, L.; Herbst-Irmer, R.; Sheldrick, G. M.; Stalke, D. Comparison of Silver and Molybdenum Microfocus X-ray Sources for Single-Crystal Structure Determination. *J. Appl. Cryst.* **2015**, *48*, 3-10. DOI: 10.1107/S1600576714022985.
3. Sheldrick, G. M. SHELXT – Integrated space-group and crystalstructure determination. *Acta Crystallogr., Sect. A: Found. Adv.* **2015**, *71*, 3-8. doi: 10.1107/S2053273314026370.
4. Sheldrick, G. M. Crystal structure refinement with SHELXL. *Acta Crystallogr., Sect. C: Struct. Chem.* **2015**, *71*, 3-8. DOI: 10.1107/S2053229614024218.
5. Dolomanov, O. V.; Bourhis, L. J.; Gildea, R. J.; Howard, J. A. K.; Puschmann, H. OLEX2: a Complete Structure Solution, Refinement and Analysis Program. *J. Appl. Cryst.* **2009**, *42*, 339-341. DOI: 10.1107/S0021889808042726.
6. Spek A. L. PLATON SQUEEZE: A Tool for the Calculation of the Disordered Solvent Contribution to the Calculated Structure Factors. *Acta Crystallogr., Sect. C: Struct. Chem.* **2015**, *71*, 9-18. DOI: 10.1107/S2053229614024929.
7. Li, J.-R.; Kuppler, R. J.; Zhou, H.-C. Selective Gas Adsorption and Separation in Metal–organic Frameworks. *Chem. Soc. Rev.* **2009**, *38* (5), 1477. DOI: 10.1039/b802426j.
8. I. Langmuir, THE DISSOCIATION OF HYDROGEN INTO ATOMS. III. THE MECHANISM OF THE REACTION.. *J. Am. Chem. Soc.* **1916**, *38*, 1145–1156. DOI: 10.1021/ja02263a001.
9. I. Langmuir, THE ADSORPTION OF GASES ON PLANE SURFACES OF GLASS, MICA AND PLATINUM.. *J. Am. Chem. Soc.* **1918**, *40*, 1361–1403. DOI: 10.1021/ja02242a004.
10. I. Langmuir, The Evaporation, Condensation and Reflection of Molecules and the Mechanism of Adsorption. *Phys. Rev.* **1916**, *8*, 149–176. DOI: 10.1103/physrev.8.149.
11. Brunauer S., Emmett P. H., Teller, E. Adsorption of gases in mutimolecular layers. *J. Am. Chem. Soc.* **1938**, *60*, 309-319. DOI: 10.1021/ja01269a023.
12. Yang, R. T. Gas Separation by Adsorption Processes, Imperial College Press, 1986.
13. Pan, H.; Ritter, J. A.; Balbuena, P. B. *Langmuir* **1998**, *14*, 6323–6327. DOI: 10.1021/la9803373
14. Whittaker, E. T.; Robinson, G. The Calculus of Observations: A Treatise on Numerical Mathematics, 4th Ed., **1967**, pp 84–87.
15. Walton K.; Sholl D. Predicting multicomponent adsorption: 50 years of the ideal adsorbed solution theory. AIChE J., **2015**, *61*, 2757-2762. DOI: 10.1002/aic.14878.
16. Groom, C. R.; Bruno, I. J.; Lightfoot, M. P.; Ward, S. C. The Cambridge Structural Database. *Acta Crystallogr., Sect. B.: Struct. Sci., Cryst. Eng. Mater.* **2016,** *72*, 171-179. DOI: 10.1107/S2052520616003954.
17. Heerden, D. P. v.; Smith, V. J.; Aggarwal, H.; Barbour, L. J. High Pressure In Situ Single-Crystal X-Ray Diffraction Reveals Turnstile Linker Rotation Upon Room-Temperature Stepped Uptake of Alkanes. *Angew. Chem. Int. Ed.* **2021**, *60* (24), 13430-13435. DOI: 10.1002/anie.202102327.
18. Banerjee, D.; Wang, H.; Plonka, A. M.; Emge, T. J.; Parise, J. B.; Li, J. Direct Structural Identification of Gas Induced Gate-Opening Coupled with Commensurate Adsorption in a Microporous Metal–Organic Framework. *Chem. Eur. J.* **2016**, *22* (33), 11816-11825. DOI: 10.1002/chem.201601784.
19. Li, J.; Han, X.; Kang, X.; Chen, Y.; Xu, S.; Smith, G. L.; Tillotson, E.; Cheng, Y.; McPherson, L. J. M.; Teat, S. J.; Rudić, S.; Ramirez-Cuesta, A. J.; Haigh, S. J.; Schröder, M. Yang, S. Purification of Propylene and Ethylene by a Robust Metal–Organic Framework Mediated by Host–Guest Interactions. *Angew. Chem. Int. Ed.* **2020**, *60* (28), 15541-15547. DOI: 10.1002/anie.202103936.
20. Wang, Y.; Huang, N.-Y.; Zhang, X.-W.; He, H.; Huang, R.-K.; Ye, Z.-M.; Li, Y.; Zhou, D.-D.; Liao, P.-Q.; Chen, X.-M.; Zhang, J.-P. Selective Aerobic Oxidation of a Metal–Organic Framework Boosts Thermodynamic and Kinetic Propylene/Propane Selectivity. *Angew. Chem. Int. Ed.* **2019**, *58* (23), 7692-7696. DOI: 10.1002/anie.201902209.
21. Yu, M.-H.; Space, B.; Franz, D.; Zhou, W.; He, C.; Li, L.; Krishna, R.; Chang, Z.; Li, W.; Hu, T.-L.; Bu, X.-H. Enhanced Gas Uptake in a Microporous Metal–Organic Framework via a Sorbate Induced-Fit Mechanism. *J. Am. Chem. Soc.* **2019**, *141* (44), 17703–17712. DOI: 10.1021/jacs.9b07807.
22. Zeng, H.; Xie, M.; Wang, T.; Wei, R.-J.; Xie, X.-J.; Zhao, Y.; Lu, W.; Li, D. Orthogonal-Array Dynamic Molecular Sieving of Propylene/Propane Mixtures. *Nature*, **2021**, *595* (7868), 542-548. DOI: 10.1038/s41586-021-03627-8.
23. Ushak, S.; Spodine, E.; Fur, E. L.; Venegas-Yazigi, D.; Pivan, J.-Y.; Schnelle, W.; Cardoso-Gil, R.; Kniep, R. Two New Hybrid Organic/Inorganic Copper(II)−Oxovanadate(V) Diphosphonates: [Cu2(phen)2(O3PCH2PO3)(V2O5)(H2O)]·H2O and [Cu2(phen)2(O3P(CH2)3PO3)(V2O5)]·C3H8. Synthesis, Structure, and Magnetic Properties. *Inorg. Chem.* **2006**, *45* (14), 5393–5398. DOI: 10.1021/ic060152g.
24. Shekhah, O.; Swaidan, R.; Belmabkhout, Y.; Plessis, M. d.; Jacobs, T.; Barbour, L. J.; Pinnau, I.; Eddaoudi, M. The Liquid Phase Epitaxy Approach for the Successful Construction of Ultra-thin and Defect-Free ZIF-8 Membranes: Pure and Mixed Gas Transport Study. *Chem. Commun.* **2014**, *50* (17), 2089-8092. DOI: 10.1039/C3CC47495J.
25. Antypov, D.; Shkurenko, A.; Bhatt, P. M.; Belmabkhout, Y.; Adil, K.; Cadiau, A.; Suyetin, M.; Eddaoudi, M.; Rosseinsky, M. J.; Dyer, M. S. Differential guest location by host dynamics enhances propylene/propane separation in a metal-organic framework. *Nat. Commun.* **2020**, *11*, 6099. DOI: 10.1038/s41467-020-19207-9.
26. Cheng, H.; Wang, Q.; Meng, L.; Sheng, P.; Zhang, Z.; Ding, M.; Gao, Y.; Bai, J. Formation of a N/O/F-Rich and Rooflike Cluster-Based Highly Stable Cu(I/II)-MOF for Promising Pipeline Natural Gas Upgrading by the Recovery of Individual C3H8 and C2H6 Gases. *ACS Appl. Mater. Interfaces* **2021**, *13* (34), 40713–40723. DOI: 10.1021/acsami.1c11971.
27. Xian, S.; Peng, J.; Pandey, H.; Thonhauser, T.; Wang, H.; Li, J. Robust Metal–Organic Frameworks with High Industrial Applicability in Efficient Recovery C3H8 and C2H6 from Natural Gas Upgrading. *Engineering* **2023**, 23, 56-63. DOI: 10.1016/j.eng.2022.07.017.
28. Yuan, B.; Wang, X.; Zhou, X.; Xiao, J.; Li, Z. Novel Room-Temperature Synthesis of MIL-100(Fe) and its Excellent Adsorption Performances for Separation of Light Hydrocarbons. *Chem. Eng. J.* **2019**, *355*, 679-686. DOI: 10.1016/j.cej.2018.08.201.
29. Qin, L.-Z.; Xiong, X.-H.; Wang, S.-H.; Zhang, L.; Meng, L.-L.; Yan, L.; Fan, Y.-N.; Yan, T.-A.; Liu, D.-H.; Wei, Z.-W.; Su. C.-Y. MIL-101-Cr/Fe/Fe-NH2 for Efficient Separation of CH4 and C3H8 from Simulated Natural Gas. *ACS Appl. Mater. Interfaces* **2022**, *14* (40), 45444–45450. DOI: 10.1021/acsami.2c13446.
30. Wu, Y.; Liu, Z.; Peng, J.; Wang, X.; Zhou, X.; Li, Z. Enhancing Selective Adsorption in a Robust Pillared-Layer Metal–Organic Framework via Channel Methylation for the Recovery of C2–C3 from Natural Gas. *ACS Appl. Mater. Interfaces* **2020**, *12* (46), 51499-51505. DOI: 10.1021/acsami.0c15267
31. Wang, L.; Zhang, W.; Ding, J.; Gong, L.; Krishna, R.; Ran, Y.; Chen, L.; Luo, F. Th-MOF Showing Six-Fold Imide-Sealed Pockets for Middle-Size-Separation of Propane from Natural Gas. *Nano Res.* **2023**, *16*, 3287-3293. DOI: 10.1007/s12274-022-4915-0.
32. Zheng, F.; Chen, R.; Zhang, Z.; Yang, Q.; Yang, Y.; Ren. Q.; Bao, Z. Cooperative Control of Intralayer and Interlayer Space in MOFs Enables Selective Capture of Intermediate-sized Molecules. *Cell Rep. Phys. Sci.* **2022**, *3* (6), 100903. DOI: 10.1016/j.xcrp.2022.100903.
33. Chen, F.; Guo, K.; Huang, X.; Zhang, Z.; Yang, Q.; Yang, Y.; Ren, Q.; Bao, Z. Extraction of Propane and Ethane from Natural Gas on Ultramicroporous Carbon Adsorbent with Record Selectivity. *Sci. China Mater.* **2023**, *66* (1), 319-326. DOI: 10.1007/s40843-022-2096-8.
34. Liu, Y.; Xu, Q.; Chen, L.; Song, C.; Yang, Q.; Zhang, Z.; Lu, D.; Yang, Y.; Ren, Q.; Bao, Z. Hydrogen-Bonded Metal-Nucleobase Frameworks for Highly Selective Capture of Ethane/Propane from Methane and Methane/Nitrogen Separation. *Nano Res.* **2022**, *15*, 7695-7702. DOI: 10.1007/s12274-022-4352-0.
35. Wu, Y.; Sun, Y.; Xiao, J.; Wang, X.; Li, Z. Glycine-Modified HKUST-1 with Simultaneously Enhanced Moisture Stability and Improved Adsorption for Light Hydrocarbons Separation. *ACS Sustainable Chem. Eng.* **2019**, *7* (1), 1557–1563. DOI: 10.1021/acssuschemeng.8b05321.
36. Sun, W.; Hu, J.; Duttwyler, S.; Wang, L.; Krishna, R.; Zhang, Y. Highly Selective Gas Separation by Two Isostructural Boron Cluster Pillared MOFs. *Sep. Purif. Technol.* **2022**, *283*, 120220. DOI: 10.1016/j.seppur.2021.120220.
37. Liu, H.; Li, B.; Zhao, Y.; Kong, C.; Zhou, C.; Lin, Y.; Tian, Z.; Chen, L. Investigation on a Zr-based Metal–Organic Framework (MOF-801) for the High-Performance Separation of Light Alkanes. *Chem. Commun.* **2021**, *57* (96), 13008-13011. DOI: 10.1039/D1CC05306J.
38. Zhou, J.; Ke, T.; Steinke, F.; Stock, N.; Zhang, Z.; Bao, Z.; He, X.; Ren, Q.; Yang, Q. Tunable Confined Aliphatic Pore Environment in Robust Metal–Organic Frameworks for Efficient Separation of Gases with a Similar Structure. *J. Am. Chem. Soc.***2022**, *144* (31), 14322–14329. DOI: 10.1021/jacs.2c05448.
39. Wang, S.-M.; Yang, Q.-Y. A Copper-Based Metal-Organic Framework for Upgrading Natural Gas through the Recovery of C2H6 and C3H8. *GreenChE*. **2023**, *4* (1), 81-87. DOI: 10.1016/j.gce.2022.04.006.
40. Yuan, Y.; Wu, H.; Xu, Y.; Lv, D.; Tu, S.; Wu, Y.; Li, Z.; Xia, Q. Selective Extraction of Methane from C1/C2/C3 on Moisture-Resistant MIL-142A with Interpenetrated Networks. *Chem. Eng. J.* **2020**, *395*, 125057. DOI: 10.1016/j.cej.2020.125057.
41. Zhang, Y.; Yang, L.; Wang, L.; Duttwyler, S.; Xing, H. A Microporous Metal-Organic Framework Supramolecularly Assembled from a CuII Dodecaborate Cluster Complex for Selective Gas Separation. *Angew. Chem. Int. Ed.* **2019**, *58* (24), 8145-8150. DOI: 10.1002/anie.201903600.
42. Zhang, Y.; Yang, Li.; Wang, L.; Cui, X.; Xing, H. Pillar Iodination in Functional Boron Cage Hybrid Supramolecular Frameworks for High Performance Separation of Light Hydrocarbons. *J. Mater. Chem. A* **2019**, *7* (48), 27560-27566. DOI: 10.1039/C9TA09928J.
43. Jia, J.; Wang, L.; Sun, F.; Jing, X.; Bian, Z.; Gao, L., Krishna, R.; Zhu, G. The Adsorption and Simulated Separation of Light Hydrocarbons in Isoreticular Metal–Organic Frameworks Based on Dendritic Ligands with Different Aliphatic Side Chains. *Chem. Eur. J.* **2014**, *20* (29), 9073-9080. DOI: 10.1002/chem.201304962.
44. Liang, W.; Xiao, H.; Lv, D.; Xiao, J.; Li, Z. Novel Asphalt-based Carbon Adsorbents with Super-high Adsorption Capacity and Excellent Selectivity for Separation for Light Hydrocarbons. *Sep. Purif. Technol.* **2018**, *190*, 60-67. DOI: 10.1016/j.seppur.2017.08.052.
45. Lv, D.; Liu, Z.; Xu, F.; Wu, H.; Yuan, W.; Yan, J.; Xi, H.; Chen, X.; Xia, Q. A Ni-based Metal-Organic Framework with Super-high C3H8 Uptake for Adsorptive Separation of Light Alkanes. *Sep. Purif. Technol.* **2021**, *266*, 118198. DOI: 10.1016/j.seppur.2020.118198.
46. Wang, W.; Xiong, X.-H.; Zhu, N.-X.; Zeng, Z.; Wei, Z.-W.; Pan, M.; Fenske, D.; Jiang, J.-J.; Su, C.-Y. A Rare Flexible Metal–Organic Framework Based on a Tailorable Mn8-Cluster Showing Smart Responsiveness to Aromatic Guests and Capacity for Gas Separation. *Angew. Chem. Int. Ed.* **2022**, *61* (26), e202201766. DOI: 10.1002/anie.202201766.
47. Zhang, Y.; Xiao, H.; Zhou, X.; Wang, X.; Li, Z. Selective Adsorption Performances of UiO-67 for Separation of Light Hydrocarbons C1, C2, and C3. *Ind. Eng. Chem. Res.* **2017**, *56* (30), 8689−8696. DOI: 10.1021/acs.iecr.6b04066.
48. Gao, S.; Morris, C. G.; Lu, Z.; Yan, Y.; Godfrey, H. G. W.; Murray, C.; Tang, C. C.; Thomas, K. Mark; Yang, S.; Schröder. Selective Hysteretic Sorption of Light Hydrocarbons in a Flexible Metal–Organic Framework Material. *Chem. Mater.* **2016**, *28* (7), 2331–2340. DOI: 10.1021/acs.chemmater.6b00443.
49. He, Y.; Zhang, Z.; Xiang, S.; Fronczek, F. R.; Krishna, R.; Chen, B. A Robust Doubly Interpenetrated Metal–Organic Framework Constructed from a Novel Aromatic Tricarboxylate for Highly Selective Separation of Small Hydrocarbons. *Chem. Commun.* **2012**, *48* (52), 6493-6495. DOI: 10.1039/C2CC31792C.
50. Shi, R.; Lv, D.; Chen, Y.; Wu, H.; Liu, B.; Xia, Q.; Li, Z. Highly Selective Adsorption Separation of Light Hydrocarbons with a Porphyrinic Zirconium Metal-Organic Framework PCN-224. *Sep. Purif. Technol.* **2018**, *207*, 262-268. DOI: 10.1016/j.seppur.2018.06.064.
51. Bloch, E. D.; Queen, W. L.; Krishna, R.; Zadrozny, J. M.; Brown, C. M.; Long, J. R. Hydrocarbon Separations in a Metal-Organic Framework with Open Iron(II) Coordination Sites. *Science* **2012**, *335* (6076), 1606-1610. DOI: 10.1126/science.1217544.
52. Li, L.; Wang, X.; Liang, J.; Huang, Y.; Li, H.; Lin, Z.; Cao, R. Water-Stable Anionic Metal–Organic Framework for Highly Selective Separation of Methane from Natural Gas and Pyrolysis Gas. *ACS Appl. Mater. Interfaces* **2016**, *8* (15), 9777–9781. DOI: 10.1021/acsami.6b00706.
53. Huang, Y.; Lin, Z.; Fu, H.; Wang, F.; Shen, M.; Wang, X.; Cao, R. Porous Anionic Indium–Organic Framework with Enhanced Gas and Vapor Adsorption and Separation Ability. *ChemSusChem*, **2014**, *7* (9), 2647-2653. DOI: 10.1002/cssc.201402206.
54. Luo, J.; Wang, J.; Cao, Y.; Yao, S.; Zhang, L.; Huo, Q.; Liu, Y. Assembly of an Indium–Porphyrin Framework JLU-Liu7: a Mesoporous Metal–Organic Framework with High Gas Adsorption and Separation of Light Hydrocarbons. *Inorg. Chem. Front.* **2017**, *4* (1), 139-143. DOI: 10.1039/C6QI00440G.
55. Wang, D.; Liu, B.; Yao, S.; Wang, T.; Li, G.; Huo, Q.; Liu, Y. A Polyhedral Metal–Organic Framework based on the Supermolecular Building Block Strategy Exhibiting High Performance for Carbon Dioxide Capture and Separation of Light Hydrocarbons. *Chem. Commun.* **2015**, *51* (83), 15287-15289. DOI: 10.1039/C5CC06162H.
56. Li, J.; Luo, X.; Zhao, N.; Zhang, L.; Huo, Q.; Liu, Y. Two Finite Binuclear [M2(μ2-OH)(COO)2] (M = Co, Ni) Based Highly Porous Metal–Organic Frameworks with High Performance for Gas Sorption and Separation. *Inorg. Chem.* **2017**, *56* (7), 4141–4147. DOI: 10.1021/acs.inorgchem.7b00156.
57. Gu, J.; Sun, X.; Kan, L.; Qiao, J.; Li, G.; Liu, Y. Structural Regulation and Light Hydrocarbon Adsorption/Separation of Three Zirconium–Organic Frameworks Based on Different V-Shaped Ligands. *ACS Appl. Mater. Interfaces* **2021**, *13* (35), 41680–41687. DOI: 10.1021/acsami.1c11224.
58. Wang, G.-D.; Krishna, R.; Li, Y.-Z.; Ma, Y.-Y.; Hou, L.; Wang, Y.-Y.; Zhu, Z. Rational Construction of Ultrahigh Thermal Stable MOF for Efficient Separation of MTO Products and Natural Gas. *ACS Materials Lett.* **2023**, 5 (4), 1091–1099. DOI: 10.1021/acsmaterialslett.3c00096.
59. Lysova, A. A.; Kovalenko, K. A.; Nizovtsev, A. S.; Dybtsev, D. N.; Fedin, V. P. Efficient separation of methane, ethane and propane on mesoporous metal-organic frameworks. *Chem. Eng. J.* **2023**, *453*, 139642. DOI: 10.1016/j.cej.2022.139642.
60. Zhang, X.-X.; Guo, X.-Z.; Chen, S.-S.; Kang, H.-W.; Zhao, Y.; Gao, J.-X.; Xiong, G.-Z.; Hou, L. A stable microporous framework with multiple accessible adsorption sites for high capacity adsorption and efficient separation of light hydrocarbons. *Chem. Eng. J.* **2023**, *466*, 143170. DOI: 10.1016/j.cej.2023.143170.
61. Cheng, H.; Wang, Q.; Bai, J. Ligand‐functional Groups Induced Tuning Mofs’ 2D into 1D Pore Channels for Pipeline Natural Gas Purification. *Chem. Eur. J.* **2023**, *29* (4), e202202047. DOI: 10.1002/chem.202202047.
62. Zhou, K.-A.; Huang, L.; Fu, X.-P.; Zhang, L.-L.; Wang, Y.-L.; Liu, Q.-Y. Fluorinated metal-organic framework for methane purification from a ternary CH4/C2H6/C3H8 mixture. *Chin. J. Struct. Chem.* Just accepted, https://doi.org/10.1016/j.cjsc.2023.100172
63. Peng, J.; Zhong, J.; Liu, Z.; Xi, H.; Yan, J.; Xu, F.; Chen, X.; Wang, X.; Lv, D.; Li, Z.. Multivariate Metal–organic Frameworks Prepared by Simultaneous Metal/ligand Exchange for Enhanced C2–C3 Selective Recovery from Natural Gas. *ACS Appl. Mater. Interfaces* **2023**, *15* (35), 41466–41475. DOI: 10.1021/acsami.3c06663.
64. Zhao, L.; Liu, P.; Deng, C.; Wang, T.; Wang, S.; Tian, Y.-J.; Zou, J.-S.; Wu, X.-C.; Zhang, Y.; Peng, Y.-L.; Zhang, Z.; Zaworotko, M. J. Robust Ultra-microporous Metal-organic Frameworks for Highly Efficient Natural Gas Purification. *Nano Res.* **2023**, *16* (10), 12338–12344. DOI: 10.1007/s12274-023-6072-5.
65. Accelrys, Materials Studio Getting Started, release 7.0, Accelrys Software, Inc., San Diego, CA, **2014**.
66. Rappe, A. K.; Goddard, W. A. Charge Equilibration for Molecular Dynamics Simulations. *J. Phys. Chem.* **1991**, *95* (8), 3358–3363. DOI: 10.1021/j100161a070.
67. Rappe, A. K.; Casewit, C. J.; Colwell, K. S.; Goddard, W. A.; Skiff, W. M. UFF, a Full Periodic Table Force Field for Molecular Mechanics and Molecular Dynamics Simulations. *J. Am. Chem. Soc.* **1992**, *114* (25), 10024–10035. DOI: 10.1021/ja00051a040.
68. Jones, J. E. On the Determination of Molecular Fields. —II. From the Equation of State of a Gas. *Proc. Roy. Soc. A* **1924**, *106* (738), 463–477. DOI: 10.1098/rspa.1924.0082.
69. Segall, M. D.; Lindan, P. J. D.; Probert, M. J.; Pickard, C. J.; Hasnip, P. J.; Clark, S. J.; Payne, M. C. J. Phys.: Condens. Matter **2002**, *14* (11), 2717-2744. DOI: 10.1088/0953-8984/14/11/301.
70. Perdew, J. P.; Burke, K.; Ernzerhof, M. Generalized Gradient Approximation Made Simple. *Phys. Rev. Lett.* **1996**, *77* (18), 3865–3868. DOI: 10.1103/physrevlett.77.3865.
71. Vanderbilt, D. Soft Self-consistent Pseudopotentials in a Generalized Eigenvalue Formalism. *Phys. Rev. B* **1990**, *41* (11), 7892–7895. DOI: 10.1103/physrevb.41.7892.
72. Grimme, S. Semiempirical Gga-type Density Functional Constructed with a Long-range Dispersion Correction. *J. Comput. Chem.* **2006**, *27* (15), 1787–1799. DOI: 10.1002/jcc.20495.
